# Supplementary material for: Efficacy and safety of dolutegravir plus emtricitabine versus standard ART for the maintenance of HIV-1 suppression: 48-week results of the factorial, randomized, non-inferiority SIMPL’HIV trial
Source: PLoS Med. 2020 Nov 10;17(11):e1003421. doi: 10.1371/journal.pmed.1003421 (PMC7654764; doi:10.1371/journal.pmed.1003421)
Supplement: S1 Table — ITT analysis. (DOCX) [file pmed.1003421.s002.docx]

|  | **PCM N = 95** | **SM N = 92** | **Risk difference**  **(95% CI)** | ***p*-value** |
| --- | --- | --- | --- | --- |
| **HIV-RNA <100 copies/ml throughout 48 weeks (± 21 days)** | 90 (94.7%) | 86 (93.5%) | +1.3%  (-5.5%; +8.0%) | 0.819 |
| **FDA snapshot (success is considered as HIV-RNA <50 cp/ml in the snapshot time window)** | 85 (89.5%) | 85 (92.4%) | -2.9%  (-11.0%; +5.4%) | 0.837 |

PCM: patient-centered monitoring; SM: standard monitoring; HIV-RNA: human immunodeficiency virus-ribonucleic acid; ITT: intention-to-treat; FDA: Food and Drug Administration.

**S1 Table: Proportion of participants with HIV-RNA <100 copies/ml throughout 48 weeks and FDA snapshot of proportion of patients with HIV-RNA <50 cp/ml at week 48 by monitoring arms. ITT analysis.**
