# Supplementary material for: Efficacy and safety of dolutegravir plus emtricitabine versus standard ART for the maintenance of HIV-1 suppression: 48-week results of the factorial, randomized, non-inferiority SIMPL’HIV trial
Source: PLoS Med. 2020 Nov 10;17(11):e1003421. doi: 10.1371/journal.pmed.1003421 (PMC7654764; doi:10.1371/journal.pmed.1003421)
Supplement: S2 Table — (DOCX) [file pmed.1003421.s003.docx]

VF: virological failure; RAM: resistance associated mutations, NA, not available, amplification not possible;DTG: dolutegravir, cART: combined antiretroviral therapy, ABC: abacavir, 3TC: lamivudine, AVG: eltegravir, COB: cobiscitat, FTC: emtricitabine, TAF: tenofovir alafenamide.

**S2 Table: Participants experiencing virological failure (according to the outcome definition)**

| **Treatment arm** | **Time of VF** | **HIV-RNA cp/mL at VF** | **HIV-RNA cp/mL**  **Re-test(s)**  2-6 weeks after | **RAM**  **at baseline** | **RAM**  **at VF (RNA)** | **Modification of treatment** | **Plasmatic level of DTG (ng/ml)** |  |
| --- | --- | --- | --- | --- | --- | --- | --- | --- |
| **According to the Primary Outcome definition: HIV-RNA ≥100 cp/mL through 48 weeks : Confirmed (twice) HIV RNA > 100 cp/ml** | | | | | | | | |
| **cART (ABC+3TC+DTG)** | Week 6 | 930 | 370 / < 20 | 41L, 69D | 41L, 69D | No | 0 |  |
| **According to the Secondary Outcome definition: HIV-RNA ≥50 cp/mL at Week 48 week** | | | | | | | | |
| **cART**  **(EVG+COBI+FTC+TAF)** | Week 48 | 80 | < 20 | No | Amplification not possible | No | X |  |
| **DTG+FTC** | Week 48 | 67 | 65 / 80 | No | No RAM | No | 4792 |  |
| **HIV RNA> 200 cp/ml (followed by HIV RNA> 50 copies/ml)** | | | | | | | |  |
| **DTG+FTC** | **Week 6** | **780** | **< 20** | **No** | **amplification not possible** | No | Not done |  |
| **DTG+FTC** | **Week 48** | **2878** | **(36 /< 20)** | **No** | **No** | No | Not done |  |
| **DTG+FTC** | **Week 12** | **3153** | **< 20** | **No** | **Ongoing** | No | Not done |  |
| **cART**  **(ABC+3TC+DTG)** | **Week 24** | **892** | **< 20** | **No** | **No** | No | Not done |  |
| **cART(ABC+3TC+NVP)** | **Week 36** | **270** | **< 20** | **No** | **Ongoing** | No | Not done |  |
