# Supplementary material for: Efficacy and safety of dolutegravir plus emtricitabine versus standard ART for the maintenance of HIV-1 suppression: 48-week results of the factorial, randomized, non-inferiority SIMPL’HIV trial
Source: PLoS Med. 2020 Nov 10;17(11):e1003421. doi: 10.1371/journal.pmed.1003421 (PMC7654764; doi:10.1371/journal.pmed.1003421)
Supplement: S3 Table — ITT analysis. (DOCX) [file pmed.1003421.s004.docx]

| **Outcome** | **PCM N = 95** | **SM N = 92** | **Risk difference (95% CI)** | ***p*-value** |
| --- | --- | --- | --- | --- |
| **Proportion of patients with at least one adverse event throughout 48 weeks** | n = 95,  62 (65.3%) | n = 92,  60 (65.2%) | -0.1%  [-13.8%;+13.6%] | 0.988 |
| **Proportion of patients with at least one serious adverse event (SAE) throughout 48 weeks** | n = 95,  10 (10.5%) | n = 92,  11 (12.0%) | -1.3% [-10.2%;+7.7%] | 0.781 |

cART: combined antiretroviral therapy, DTG: dolutegravir, FTC: emtricitabine, PCM: patient-centered monitoring, SM: standard monitoring, ITT: intention-to-treat, SAE: serious adverse event, CI: confidence interval.

**S3 Table**: **Proportions of reported adverse events by monitoring arms. ITT analysis.**
