# Supplementary material for: Efficacy and safety of dolutegravir plus emtricitabine versus standard ART for the maintenance of HIV-1 suppression: 48-week results of the factorial, randomized, non-inferiority SIMPL’HIV trial
Source: PLoS Med. 2020 Nov 10;17(11):e1003421. doi: 10.1371/journal.pmed.1003421 (PMC7654764; doi:10.1371/journal.pmed.1003421)
Supplement: S4 Table — (DOCX) [file pmed.1003421.s005.docx]

| **Treatment arm** | **Severe adverse event** | **Resolved at week 48** |
| --- | --- | --- |
| cART | Symptomatic ovarian cyst | Yes |
| cART | Acute retinal detachment | Yes |
| cART | Hospitalization due to relapse of retinal detachment; | Yes |
| cART | Removal silicon oil after retinal detachment | Yes |
| DTG+FTC | Acute confusion | Yes |
| cART | Acute appendicitis | Yes |
| cART | Pyelonephritis | Yes |
| cART | Dyspnoea | Yes |
| cART | Hospitalisation for inguinal hernia | Yes |
| cART | Elective hospitalisation for orthopedic implant removal | Yes |
| cART | Multiple bone fractures | Yes |
| DTG+FTC | Paraesthesia and painful neuropathy | Yes |
| cART | Streptococcus bacteraemia | Yes |
| cART | Acute appendicitis | Yes |
| cART | Elective hospitalisation for excision cervical lymph node | Yes |
| DTG+FTC | Suicidal Ideation | Yes |
| cART | Elective hospitalisation for hip replacement | Yes |
| DTG+FTC | Perineal abscess | Yes |
| cART | Pyelonephritis | Yes |
| cART | Epididymitis | Yes |
| DTG+FTC | Extra-peritoneal repair for inguinal hernia both sides | Yes |
| DTG+FTC | Perianal abscess | Yes |
| cART | *Haemophilus influenza* pneumonia | Yes |
| cART | Erysipelas | Yes |
| cART | Surgery for lumbar hernia | Yes |
| cART | Urinary sepsis with acute renal failure | Yes |
| cART | Transurethral resection of the prostate | Yes |

cART: combined antiretroviral therapy, DTG: dolutegravir, FTC: emtricitabine

**S4 Table**: **List of reported serious adverse events through 48 weeks.**
