# Supplementary material for: Efficacy and safety of dolutegravir plus emtricitabine versus standard ART for the maintenance of HIV-1 suppression: 48-week results of the factorial, randomized, non-inferiority SIMPL’HIV trial
Source: PLoS Med. 2020 Nov 10;17(11):e1003421. doi: 10.1371/journal.pmed.1003421 (PMC7654764; doi:10.1371/journal.pmed.1003421)
Supplement: S2 Text — (PDF) [file pmed.1003421.s007.pdf]

Clinical Study Protocol

Evaluation of a simplified strategy for the long-term management of HIV infection: a non-inferiority, randomized, controlled, open-label clinical trial

The Simpl'HIV trial

Study Type:	Clinical trial with Investigational Medicinal Product (IMP)
Study Categorisation:	A
Study Registration:	www.clinicaltrials.gov (registration number: NCT03160105)<br>FOPH portal (registration number: NCT03160105)
Study Identifier:	SIMPL'HIV
Sponsor, Sponsor-Investigator	Prof. Alexandra Calmy
Principal Investigator:	Dr Delphine Sculier and Dr Gilles Wandeler
Investigational Product:	Dolutegravir (Tivicay®) 50 mg + Emtricitabine (Emtriva®) 200 mg
Protocol Version and Date:	Version 1.0, 21 DEC 2016<br>Version 2.0, 24 FEB 2017<br>Version 3.0, 27 JUN 2017<br>Version 4.0, 02 JAN 2018<br>Version 5.0, 18 JUN 2018<br>Version 5.1, 30 JUL 2019<br>Add if applicable, the Amendment number, from (date), replaces version number from (date)

CONFIDENTIAL

The information contained in this document is confidential and the property of the sponsor/investigator. The information may not - in full or in part - be transmitted, reproduced, published, or disclosed to others than the applicable Competent Ethics Committee(s) and Regulatory Authority(ies) without prior written authorisation from the sponsor except to the extent necessary to obtain informed consent from those who will participate in the study.

Signature Page(s)

Study number www.clinicaltrials.gov (registration number: NCT03160105)

Study Title **Evaluation of a simplified strategy for the long-term management of HIV infection: a non-inferiority, randomized, controlled, open-label clinical trial**

The Sponsor-Investigator and Principal Investigators have approved the protocol version [5.1 of 30.07.2019)], and confirm hereby to conduct the study according to the protocol, current version of the World Medical Association Declaration of Helsinki, ICH-GCP guidelines or ISO 14155 norm if applicable and the local legally applicable requirements.

Sponsor-Investigator: Pre Alexandra Calmy

Place/Date	Signature

Principal Investigator: Dr Delphine Sculier

Place/Date	Signature

Principal Investigator: Dr Gilles Wandeler

Place/Date	Signature

I have read and understood this trial protocol and agree to conduct the trial as set out in this study protocol, the current version of the World Medical Association Declaration of Helsinki, ICH-GCP guidelines or ISO 14155 norm and the local legally applicable requirements.

Site	Service of Infectious Diseases, Lugano Regional Hospital, Via Tesserete 46, CH-6903 Lugano
Principal investigator	Prof Enos Bernasconi

Place/Date   Signature

I have read and understood this trial protocol and agree to conduct the trial as set out in this study protocol, the current version of the World Medical Association Declaration of Helsinki, ICH-GCP guidelines or ISO 14155 norm and the local legally applicable requirements.

Site	Department of Infectious Diseases and Hospital Epidemiology, University Hospital of Zurich, Rämistrasse 100, 8091 Zürich
Principal investigator	Dr Dominique Braun

________________________ ________________________________

Place/Date Signature

I have read and understood this trial protocol and agree to conduct the trial as set out in this study protocol, the current version of the World Medical Association Declaration of Helsinki, ICH-GCP guidelines or ISO 14155 norm and the local legally applicable requirements.

Site	Infectious Diseases Service, Lausanne University Hospital, Rue du Bugnon, 46, 1011 Lausanne
Principal investigator	Dr Matthias Cavassini

Place/Date Signature

I have read and understood this trial protocol and agree to conduct the trial as set out in this study protocol, the current version of the World Medical Association Declaration of Helsinki, ICH-GCP guidelines or ISO 14155 norm and the local legally applicable requirements.

Site	Department of Infectious Diseases and Hospital Epidemiology, University Hospital of Basel, Petersgraben 4, 4051 Basel
Principal investigator	Dr Marcel Stoeckle

Place/Date	Signature

I have read and understood this trial protocol and agree to conduct the trial as set out in this study protocol, the current version of the World Medical Association Declaration of Helsinki, ICH-GCP guidelines or ISO 14155 norm and the local legally applicable requirements.

Site	Division of Infectious Diseases and Hospital Epidemiology, Kantonspital St.Gallen, Rorschacher Str. 95, 9007 St. Gallen
Principal investigator	Dr. Patrick Schmid

Place/Date	Signature

TABLE OF CONTENTS

STUDY SYNOPSIS **13**
STUDY SUMMARY IN LOCAL LANGUAGE **17**
ABBREVIATIONS **19**
STUDY SCHEDULE **21**
1. STUDY ADMINISTRATIVE STRUCTURE **23**
1.1 Sponsor, Sponsor-Investigator 23
1.2 Principal Investigator(s) 23
1.3 Methodologists and Statistician ("Biostatistician") 24
1.4 Laboratory 24
1.5 Monitoring institution 24
1.6 Data Safety Monitoring Committee 25
1.7.1. Study Scientific Committee: 25
1.7.2. Trial Team Coordinators at the coordinating centre Geneva 25
2. ETHICAL AND REGULATORY ASPECTS **27**
2.1 Study registration 27
2.2 Categorisation of study 27
2.3 Competent Ethics Committee (CEC) 27
2.4 Competent Authorities (CA) 27
2.5 Ethical Conduct of the Study 27
2.6 Declaration of interest 27
2.7 Patient Information and Informed Consent 28
2.8 Participant privacy and confidentiality 28
2.9 Early termination of the study 28
2.10 Protocol amendments 28
3. BACKGROUND AND RATIONALE **29**
3.1 Background and Rationale 29
3.2 Investigational Product (treatment) and Indication 31
3.3 Preclinical Evidence 32
3.4 Clinical Evidence to Date 33
3.5 Dose Rationale 34
3.6 Explanation for choice of comparator 34
3.7 Risks / Benefits 34
3.8 Justification of choice of study population 36
4. STUDY OBJECTIVES **37**
4.1 Overall Objective 37
4.2 Primary Objective 37
4.3 Secondary Objectives 37
4.4 Safety Objectives 37
5. STUDY OUTCOMES **38**
5.1 Primary Outcomes 38
5.2 Secondary Outcomes 38
5.3 Other Outcomes of Interest 38
5.4 Safety Outcomes 38
6. STUDY DESIGN **39**
6.1 General study design and justification of design 39

Patient-centered monitoring 41
6.2 Methods of minimising bias 41
6.2.1 Randomisation 41
6.2.2 Blinding procedures 41
6.2.3 Other methods of minimising bias 42
6.3 Unblinding Procedures (Code break) 42
7. STUDY POPULATION 43
7.1 Eligibility criteria 43
7.2 Recruitment and screening 44
7.2.1 Recruitment strategies 44
7.2.2 Feasibility of recruitment 44
7.3 Assignment to study groups 44
7.3.1 Allocation sequence 44
7.3.2 Concealment mechanism 44
7.3.3 Implementation 44
7.4 Criteria for withdrawal / discontinuation of participants 45
8. STUDY INTERVENTION 46
8.1 Identity of Investigational Products (treatment) 46
8.1.1 Experimental Intervention (treatment) 46
8.1.2 Control Intervention (standard/routine/comparator treatment) 48
8.1.3 Packaging, Labelling and Supply (re-supply) 48
8.1.4 Storage Conditions 48
8.2 Administration of experimental and control interventions 48
8.2.1 Experimental Intervention 48
8.2.2 Control Intervention 49
8.3 Dose modifications 49
8.4 Compliance with study intervention 49
8.4.1 Retention 50
8.5 Data Collection and Follow-up for withdrawn participants 50
8.6 Trial specific preventive measures 50
8.7 Concomitant Interventions (treatments) 50
8.8 Study Drug Accountability 51
8.9 Return of Study Drug 51
9. STUDY ASSESSMENTS 51
9.1 Study flow chart(s) / table of study procedures and assessments 52
9.2 Assessments of outcomes 54
9.2.1 Assessment of Primary Outcomes 54
9.2.5 Assessments in participants who prematurely stop the study 56
9.3 Procedures at each visit 56
9.3.1. Screening, visit 1 and informed consent (Week -4 to week-1) at SHCS center 56
9.3.2. Baseline, visit 2 (Day 0) at SHCS center 57
9.3.4. Week 6 (± 7 days) 57
9.3.5. Weeks 12 and 36 (± 7 days) 58
9.3.6. Week 24 (± 7 days) at SHCS center 58
9.3.7. Week 48 (± 21 days) or study discontinuation visit at SHCS center: 59

10. SAFETY 60
10.1 Drug studies 60
10.1.1. Definition and assessment of (Serious) Adverse Events and other safety related events . 60
10.1.2. Reporting of (Serious) Adverse Events and other safety related events 61
10.1.3 Follow up of (Serious) Adverse Events and other safety related events 62
11. STATISTICAL METHODS 63
11.1 Hypotheses 63
11.2 Determination of Sample Size 63
11.3 Statistical criteria of termination of trial 63
11.4 Planned Analyses 64
11.4.4 Interim analyses 66
11.4.5 Safety analysis 66
11.4.6 Deviation(s) from the original statistical plan 66
11.5 Handling of missing data and drop-outs 66
12. QUALITY ASSURANCE AND CONTROL 67
12.1 Data handling and record keeping / archiving 67
12.1.1 Case Report Forms 67
12.1.2 Source data 67
12.1.3 Analysis and Record keeping / archiving 67
12.2 Data management 67
12.3 Monitoring 68
12.4 Audits and Inspections 68
12.5 Confidentiality, Data Protection 68
12.6 Storage of biological material and related health data 68
13. POST-STUDY 69
14. PUBLICATION AND DISSEMINATION POLICY 72
15. FUNDING AND SUPPORT 72
15.1 Funding 72
15.2 Other Support 72
16. INSURANCE 72
17. STUDY AGREEMENT 72
18. REFERENCES 73
19. APPENDIX 79
20. SUPPLEMENTARY FILES 80

Study synopsis

**Sponsor / Sponsor-Investigator**	Pre Alexandra Calmy
**Study Title:**	Evaluation of a simplified strategy for the long-term management of HIV infection: a non-inferiority, randomized, controlled, open-label clinical trial
**Short Title / Study ID:**	SIMPL'HIV trial
**Protocol Version and Date:**	Version 1.0, 21 DEC 2016<br>Version 2.0, 24 FEB 2017<br>Version 3.0, 27 JUN 2017<br>Version 4.0, 02 JAN 2018<br>Version 5.0, 18 JUN 2018<br>Version 5.1, 30 JUL 2019
**Trial registration:**	https://www.clinicaltrials.gov/
**Study category and Rationale**	A – Dolutegravir 50 mg (DTG, Tivicay®) is used as a dual therapy in this protocol, i.e. in association with other antiretroviral agents as indicated in the product information.
**Clinical Phase:**	Phase IV
**Background and Rationale:**	At present, HIV treatment guidelines recommend a combination of three drugs for the initiation and maintenance of antiretroviral therapy (cART). Simplification of treatment and care is considered critical to further scale-up treatment, support retention in care, and reduce costs. Current simplified maintenance regimens are mainly based on boosted protease inhibitor-based mono and dual therapies. However, many HIV-infected individuals are still excluded from this niche, either due to suboptimal virological control or a lack of the necessary infrastructure to monitor such a regimen as is the case in resource-limited settings.<br>Dolutegravir (DTG) is a once-daily integrase inhibitor that shows very good tolerability, high potency, very few drug-drug interactions and a distinctive resistance profile in combination therapy. These characteristics suggest that DTG could be an acceptable and efficient alternative to protease inhibitors in simplified maintenance therapy when given together with a nucleoside reverse transcriptase inhibitor (NRTI).<br>The efficacy of a dual therapy of DTG with 3TC (lamivudine) was demonstrated in a large, fully powered, randomized clinical trial of ART-naïve individuals. A single arm, open label pilot study of 90 patients tested the association of DTG with 3TC as a de-escalation strategy and showed no alarming signal. In addition, a large randomized trial conducted by ViiV pharmaceutical has shown that a dual therapy combining rilpivirine (RIL) and DTG was non inferior to standard cART. In our trial, we chose to study a novel, soon generically available, dual maintenance therapy including DTG and emtricitabine (FTC).<br>Simplification also includes optimized monitoring, such as simplified laboratory monitoring, task-shifting of ART management from doctors to nurses or decentralization of care. To our knowledge, these strategies have only been tested in resource-limited settings.<br>We aim to demonstrate, in a multicentre, non-inferiority, open-label, randomized controlled trial that maintenance therapy could be safely simplified to DTG-based dual therapy in association with FTC and to patient-centered monitoring once virological suppression is achieved.

**Objective(s):**	The trial will address two primary objectives:<br>1. To assess the efficacy of DTG-based maintenance therapy in virologically suppressed HIV-infected individuals who are switched from standard therapy (cART) to DTG + FTC dual therapy.<br>2. To assess the costs of a patient-centered ART monitoring which offers participants alternative options for venipuncture and blood analysis/drug delivery/study visits and omits CD4 cell counts and safety blood tests during follow-up.
**Outcome(s):**	1. The first primary outcome will be the maintenance of HIV-RNA <100 copies/ml through the 48-week study duration.<br>2. Secondary outcomes will include the maintenance of HIV-RNA <50 copies/ml through 48 weeks of follow-up (classical proportion and by FDA snapshot), loss of future drug options, change in CD4 cell count, DTG and FTC plasma levels, safety, treatment satisfaction, monitoring satisfaction, health-related quality of life, study satisfaction, and costs.The second primary outcome will be to assess the direct costs of patient-centered monitoring.<br>Secondary outcomes will include acceptability, safety, satisfaction and cost-effectiveness of monitoring arms.
**Study design:**	This is a pragmatic multicentre, 2x2 factorial randomized controlled trial with 1:1:1:1 randomization to switching to DTG-based maintenance dual therapy in association with FTC or continuation of cART, and to patient-centered monitoring or continuation of standard monitoring.<br>The randomized comparisons will examine whether DTG + FTC dual therapy is non-inferior in terms of viral suppression compared to standard therapy, and whether patient-centered monitoring is cost-saving and acceptable compared to standard monitoring.<br>Patients will be followed during 48 weeks.<br>A sample size of 92 patients in each group (DTG + FTC dual therapy and standard cART) will be required to demonstrate non-inferiority with a non-inferiority (NI) margin of 12%.
**Inclusion / Exclusion criteria:**	Inclusion criteria:<br>1. Informed consent as documented by signature;<br>2. Documented HIV-1 infection;<br>3. Enrolled in the SHCS or receiving care from a medical doctor of the SHCS network;<br>4. ≥ 18 years of age;<br>5. HIV-RNA <50 copies/mL at screening and for at least 24 weeks before screening on effective suppressive cART, one blip with less than 200 copies/mL being allowed during this period if followed by at least 2 results < 50 copies/mL.<br>6. On standard cART at the time of inclusion, i.e.:<br>- 2 NRTIs + either 1 NNRTI, 1 boosted PI or 1 INSTI;<br>- NRTI-sparing triple ARV regimen (e.g. 1 NRTI + 1 NNRTI + 1 InSTI);<br>- Dual therapy with protease inhibitor.<br><br>Exclusion criteria:<br>1. HIV-2 infection;<br>2. Previous ART change for unsatisfactory virological response, i.e slow initial virological suppression, incomplete suppression or rebound. Change of drug or drug class for convenience or toxic effect prevention or management is allowed.<br>*Note: patients with documented genotype(s) presenting only a M184V mutation remain eligible.*

	3. Creatinine clearance < 50ml/min;<br>4. ASAT or ALAT >2.5x upper limit of the norm;<br>5. Known hypersensitivity, intolerance or allergy to DTG or FTC;<br>6. Known or suspected non-adherence (defined as <80% adherence, i.e. missed dose > 1x/week), to current treatment in the last 6 months;<br>7. Concomitant use of drugs that decrease DTG blood concentrations including carbamazepine, oxcarbamazepine, phenytoin, phenobarbital, St John's wort and rifampicin;<br>8. Women who are pregnant or breast-feeding;<br>9. a. Presence of any InSTI-resistance.<br>b. Non availability of previous routine resistance test, at least for reverse transcriptase and protease genes.<br>*Note: Subjects remain eligible in the absence of any previous resistance test only if they are on their first-line antiretroviral regimen.*<br>10. Evidence of acute or chronic hepatitis B virus infection based on results of serology testing:<br>- subjects positive for HBsAg are excluded (corresponding to acute or chronic infection);<br>- only positive anti-HBc subjects will be excluded if positive HBV-DNA;<br>- susceptible[1] (negative HBsAg, anti-HBc and anti-HBs), immune due to hepatitis B vaccination (negative HBsAg and anti-HBc and positive anti-HBs) and immune due to natural infection (negative HBsAg and positive anti-HBc and anti-HBs) subjects are not excluded.<br><br>Patients enrolled in other interventional studies are not elibible. After the other interventional study termination, they will have to wait at least 3 months before the Simpl'HIV screening.
**Measurements and procedures:**	*Intervention*: DTG 50mg 1x / day + FTC 200 mg 1x / day<br><br>*Clinical procedures*: participants will be screened up to 4 weeks prior to the baseline visit and, if eligible, will be seen at the baseline visit, at week 6 and week 12, and twelve-weekly afterwards (weeks -4, 0, 6, 12, 24, 36 and week 48). During each of these visits starting from the baseline visit, adherence to study drug will be assessed, concomitant medications and adverse events will be evaluated.<br><br>*Laboratory procedures*: besides HIV viral load performed at each study visit, the following laboratory assessments will be performed for all patients:<br>• Screening visit: CD4 count, full blood count, safety serum chemistry, glucose and lipid profile;<br>• Baseline visit: HIV-DNA level in peripheral blood;<br>• Week 48: HIV-DNA level in peripheral blood, CD4 count, full blood count, safety serum chemistry, glucose and lipid profile, DTG and FTC plasma concentration for patients receiving DTG and FTC);<br>• Beta HCG (urinary pregnancy test) at screening visit and every 6 months thereafter (i.e. week 24 and 48 visits) in women of childbearing potential;<br>• DTG + FTC concentration in plasma for patients taking DTG and/or FTC in case of HIV-RNA ≥ 100 copies/ml (if HIV-RNA is

[1] HBV-susceptible subjects will be offered with immunization as standard-of-care practice.

	twice consecutively ≥ 100 copies/ml, this measure will be performed only on the sample with the highest value);<br>• Genotypic resistance in case of virological failure (defined as two HIV-RNA measurements ≥ 100 copies/ml 2 weeks apart);<br>• Pharmacogenetics analysis in case of virological failure (defined as two HIV-RNA measurements ≥ 100 copies/ml 2 weeks apart);<br>• Plasma samples will be stored at each study visit and used for dolutegravir and emtricitabine plasma concentration measurement, genotypic testing and pharmacogenetics analysis in patients with virological failure.<br><br>For patients in the standard monitoring arm, additional laboratory assessments will be performed as per SHCS regular assessments:<br>• Weeks 12, 24, 36: CD4 count, full blood count, safety serum chemistry, glucose and lipid profile.<br><br>*Questionnaires*: health-related quality of life and patient's monitoring satisfaction questionnaires will be performed for all patients at baseline and weeks 12 and 48.
**Study Product / Intervention:**	<u>Dual therapy arm</u>: patients randomized to this arm will be switched to DTG + FTC dual maintenance therapy. DTG 50 mg will be administered together with FTC 200 mg once daily as dual therapy for a duration of 48 weeks.<br>Patient-centered monitoring arm: patients randomized to this arm will have immunological and safety blood analyses (CD4 cell count, lipids and glucose, renal and hepatic function tests, creatine kinase) performed at screening and at week 48. Additional safety blood tests will be performed only in case of signs of symptoms as indicated by physician.<br>In addition, patients will be offered alternative options for the conduction of study visits, of venipunctures and blood analyses and of drugs delivery at weeks 6, 12 and 36. Participants will be asked to select at least one of the three following strategies:<br>• Venipuncture for blood analyses: decentralised venipuncture, i.e. at peripheral laboratory near patient's home or place of work, or with general practitioner, versus at their affiliated SHCS site;<br>• Drug delivery: delivery of ARV drugs/prescription by mail or at a chosen pharmacy versus at their affiliated SHCS site;<br>Assessment and clinical interview (adherence, side effects, concomitant medications): phone call with study nurse/physician versus face-to-face at their affiliated SHCS site.
**Control Intervention**	Standard-of-care arm: patients randomized to this arm will continue their current standard ART regimen (cART).<br>Standard monitoring arm: patients randomized to this arm will continue a standard 3-monthly routine safety biological monitoring (including CD4 cell count, lipids and glucose, renal and hepatic function tests, creatine kinase) at their SHCS site.
**Post-study follow-up**	At week 48 visit, participants will give their informed consent to participate in the post-study period including a follow-up up to week 144 with an annual visit.<br>Outcomes will include proportion of patients maintaining HIV suppression, proportion of patients experiencing loss of future drug options, time to loss of virological response (TLOVR), assessment of predictors of virological failure, the change in several laboratory in CD4 cell count, safety, quality

	of life, ART and monitoring changes during post-study, patient's treatment and monitoring satisfaction, adherence to treatment.<br>Week 96 and 144 visits include clinical procedures (ART changes and reasons for changes; monitoring changes; current treatment and monitoring satisfaction; number, date and value of HIV-RNA performed; number, date and results of genotypes resistance testing performed; grade 3 or 4 AE and SAE occurrence; guality of life; adherence questionnaire; patient's weight and blood pressure) and laboratory procedures (HIV-1 RNA in plasma; CD4 count; full blood count; safety serum chemistry inluding creatinine, ASAT/ALAT, alkaline phosphatases, glucose and lipid profile).
**Number of Participants with Rationale:**	Total number of participants: 184<br>Number in each group: 92<br>A sample size of 92 patients in each group (DTG + FTC dual therapy and standard cART) will be required to demonstrate non-inferiority with a non-inferiority (NI) margin of 12%.
**Study Duration:**	1 year of preparation period (June 2016 to May 2017)<br>1 year of recruitment period (May 2017 to June 2018)<br>2 years of study duration (May 2017 to May 2019)<br>2 years of post-study duration (May 2018 to May 2021)
**Study Schedule:**	Start of preparation period: June 2016<br>First-Participant-In: May 2017<br>Last-Participant-Out (planned): May 2021
**Investigator(s):**	Sponsor-Investigator:<br>- Prof Alexandra Calmy, MD, PhD, D, Medical specialties department, Infectious diseases consultation, University Hospitals of Geneva, rue Gabrielle-Perret-Gentil 4, 1211 Genève 14, Switzerland, Alexandra.Calmy@hcuge.ch<br><br>Principal Investigators (PI):<br>- Dr Delphine Sculier, MD, MSc, Avenue de Champel 6, 1206 Genève, Switzerland. Delphine.sculier@hin.ch<br>- Dr Gilles Wandeler, MD MSc, Departement of Infectious Disease, Bern University Hospital, University of Bern, 3010 Bern, Switzerland, gilles.wandeler@ispm.unibe.ch<br><br>Site-Principal Investigators (PI):<br>- Dr Delphine Sculier, MD, MSc, Avenue de Champel 6, 1206 **Genève**, Switzerland. Delphine.sculier@hin.ch<br>- Dr Gilles Wandeler, MD MSc, Departement of Infectious Disease, **Bern** University Hospital, University of Bern, 3010 Bern, Switzerland, gilles.wandeler@ispm.unibe.ch<br>- Prof Enos Bernasconi, Dr.med., Serviceof Infectious Diseases, **Lugano** Regional Hospital, Via Tesserete 46, CH-6903 Lugano, Switzerland, Enos.Bernasconi@eoc.ch<br>- Dr Dominique Braun, Dr. med, Department of Infectious Diseases and Hospital Epidemiology, University Hospital of **Zurich**, Rämistrasse 100, 8091 Zürich, Zurich Switzerland, Dominique.Braun@usz.ch<br>- Dr Matthias Cavassini, MD, PD, Infectious Diseases Service, **Lausanne** University Hospital, Rue du Bugnon, 46, 1011 Lausanne, Switzerland, Matthias Cavassini@chuv.ch<br>- Dr Marcel Stoeckle, MD, Department of Infectious Diseases and Hospital Epidemiology, University Hospital of Basel, Spitalstrasse 21,

	4056 **Basel**, Switzerland, Marcel.Stoeckle@usb.ch<br>- Dr. Patrick Schmid, MD, Division of Infectious Diseases and Hospital Epidemiology, Kantonspital **St.Gallen**, Rorschacher Str. 95, 9007 St. Gallen, Switzerland, Patrick.schmid @kssg.ch
**Study Centre(s):**	Multi-centre:<br>- Geneva: University Hospitals of Geneva, Infectious diseases consultation<br>- Lausanne: Centre Hospitalier Universitaire Vaudois, Infectious disease consultation<br>- Bern: Departement of Infectious Diseases, Bern university Hospital, University of Bern<br>- Basel: University Hospital of Basel, Department of Infectious Diseases and Hospital Epidemiology<br>- Zurich: University Hospital of Zurich, Department of Infectious Diseases and Hospital Epidemiology<br>- Lugano: Lugano Regional Hospital, Service of Infectious Diseases<br>- St-Gallen: Kantonspital St. Gallen, Division of Infectious Diseases and Hospital Epidemiology
**Statistical Considerations:**	DTG + FTC dual therapy is expected to be non- inferior to standard-of-care using a 12% non-inferiority margin assuming that 92% of patients are virologically suppressed at week 48 in the two comparison arms.<br>We will evaluate the proportion of patients with viral suppression at week 48 in each arm, and compute a Mantel-Haenszel risk difference (DTG+FTC minus cART) stratified by monitoring type with a two-sided 95% confidence interval. If the lower confidence limit is higher than -12% we will conclude non-inferiority. If the lower confidence limit is below -12% we will conclude that DTG + FTC is potentially inferior to cART. Per protocol (PP) and intention-to-treat (ITT) analyses will be performed. Exclusion criteria for PP will be: patients who discontinue treatment prematurely for reasons other than death, patients with a treatment adherence of less than 80%, or those who do not have HIV-1 RNA results available at week 48. For ITT analysis, all randomized patients will be analysed in the allocated group regardless of any protocol violations.
**GCP Statement:**	This study will be conducted in compliance with the protocol, the current version of the Declaration of Helsinki, the ICH-GCP or ISO EN 14155 (as far as applicable) as well as all national legal and regulatory requirements.

STUDY SUMMARY IN LOCAL LANGUAGE

GERMAN

Nach derzeitigen Empfehlungen wird für die Einleitung und Erhaltung der antiretroviralen Therapie eine Kombination von drei Medikamenten angeboten. Vereinfachungen der Therapie und der Pflege werden in Bezug auf eine verbesserte Patientenbindung sowie reduzierte Gesundheitskosten als kritisch betrachtet. Aktuell werden vereinfachte Erhaltungstherapien selten eingesetzt und basieren meist auf verstärkten Protease-Inhibitoren oder Dual-Therapien.

Dolutegravir ist ein Integrase-Inhibitor, mit ausgezeichneter Toleranz, sehr guter Wirksamkeit, wenig Wechselwirkungen mit anderen Medikamenten und einer hohen genetischen Resistenzbarriere in kombinierter Therapie. Diese Eigenschaften deuten darauf hin, dass Dolutegravir eine akzeptable und wirksame Alternative als vereinfachte Erhaltungstherapie sein könnte (mit nukleosidischer Reverse-Transkiptase-Inhibitoren).

Dies ist eine multizentrische klinische Studie, welche die Wirksamkeit von Dolutegravir und Emtricitabine in Dual-Therapie als Erhaltungstherapie bei freiwilligen, virussupprimierten Patienten untersucht. Ziel ist es aufzuzeigen, dass die Erhaltungstherapie in Bezug auf die Virussuppression nicht schlechter ist als die Standard-Dreifachtherapie, und dass vereinfachte Laborkotrollen in Bezug auf Kosten, Akzeptanz, Sicherheit und Lebensqualität für die Patientenden Standard-Kontrollen überlegen sind.

184 Teilnehmer werden aus der Schweizerischen Kohortenstudie rekrutiert. Diese Patienten haben eine nicht nachweisbare Virämie seit mindestens 24 Wochen unter herkömmlicher Dreifachtherapie und kein Therapieversagen mit Integrase-Inhibitoren in der Vergangenheit. Die Teilnehmer werden nach Zufallsprinzip eingeteilt (randomisiert) und wechseln entweder auf Dolutegravir und Emtricitabine als Dual-Therapie oder führen ihre Standard-Therapie fort; eine zweite Randomisierung teilt sie entweder der vereinfachten oder der Standard-Laborüberwachung zu. Bei allen Patienten werden während 48 Wochen die Adhärenz und die Virämie, zuerst in einem Abstand von 6 Wochen und nach dem ersten Quartal vierteljährlich kontrolliert.

Allen Patienten die Woche 48 der Studie abgeschlossen haben, können ihre Zustimmung geben, die Nachbeobachtung auf 3 Jahre mit 1 Besuch pro Jahr zu verlängern.

Diese Studie ist insofern verallgemeinerbar, da wir Patienten aus dem Netzwerk der Schweizerischen Kohorte rekrutieren. Darüberhinaus beabsichtigen wir zu zeigen, dass eine vereinfachte Erhaltungstherapie gut verträglich und auch in Regionen mit limitierten Ressourcen durchführbar sein kann.

Mit einer Nachbeobachtung von 144 Wochen, hoffen wir, die Frage nach der Wirksamkeit der Dolutegravir und Emtricitabine Duatl-Therapie mit ausreichender Teststärke und annähernd dem Kontext des wirklichen Lebens beantworten zu können.

FRANÇAIS

Les recommandations de traitement proposent actuellement une combinaison de trois médicaments pour l'initiation et la maintenance d'une thérapie antirétrovirale. La simplification du traitement et de son suivi est considérée comme critique pour améliorer la rétention en soins et réduire les coûts de la santé. Les traitements simplifiés de maintenance actuels sont peu utilisés et reposent sur l'utilisation d'antiprotéases boostées ou de bithérapies.

Le dolutegravir est un inhibiteur de l'intégrase, avec une excellente tolérance, une très bonne efficacité et des interactions médicamenteuses limitées, ainsi qu'une haute barrière génétique à la résistance en traitement combiné. Ces caractéristiques suggèrent que le dolutegravir pourrait être une option acceptable et efficace en traitement de maintenance simplifié (avec inhibiteur nucléosidique de la reverse transcriptase).

Il s’agit d’un essai clinique multicentrique visant à tester l’efficacité d’un traitement de maintenance par dolutegravir et emtricitabine en bithérapie chez des patients volontaires et en suppression virologique. L’objectif est de démontrer que cette thérapie de maintenance n’est pas inférieure en terme de suppression virologique, en comparaison avec une thérapie antirétrovirale standard, et qu’un suivi de traitement simplifié est bénéfique en terme de coût, d’acceptabilité, de sécurité et de qualité de vie pour les patients si on le compare au suivi standard.

184 participants seront recrutés principalement au sein de l’Etude suisse de cohorte VIH. Ces patients auront une charge virale indétectable depuis au moins 24 semaines, sous thérapie antirétrovirale conventionnelle, sans antécédent d’échec au traitement antirétroviral. Les participants seront randomisés pour recevoir soit une bithérapie de dolutegravir et emtricitabine, soit pour poursuivre leur thérapie standard; une seconde randomisation les assignera soit à un suivi décentralisé et allégé, soit à un suivi standard. Tous les patients seront vus durant 48 semaines, avec des visites fréquentes de vérification d’adhérence et de la charge virale, aux 6 semaines sur le premier trimestre puis de manière trimestrielle. Les patients ayant terminé la semaine 48 de l’étude, pourront donner leur consentement pour étaindre le suivi post-étude à 3 ans avec 1 visite par année.

Cet essai se veut généralisable car nous comptons prouver qu’un traitement de maintenance simplifié pourrait être bien toléré et disponible également dans les régions à ressources limitées.

Avec un suivi étendu à 144 semaines, nous espérons répondre à la question de l’efficacité du dolutégravir et de l’emtricitabine en bithérapie avec une puissance suffisante pour nous approcher du contexte de la vie réelle.

ITALIAN

Le raccomandazioni attuali per la terapia propongono una combinazione di tre farmaci per l’inizio e il mantenimento di una terapia antiretrovirale. La semplificazione della terapia e del follow-up è considerata critica per il mantenimento in terapia e per ridurre i costi della salute. Le attuali terapie semplificate di mantenimento sono poco utilizzate et sono basate sull’uso di antiproteasi con ritonavir o di biterapie.

Il dolutegravir è un inibitore dell’intergasi con un’eccellente tollerabilità, un’efficacità molto buona, poche interazioni medicamentose nonchè un’alta barriera genetica allo sviluppo di resistenze in caso di terapia combinata. Queste caratteristiche suggeriscono che il dolutegravir potrebbe essere un’opzione accettabile ed efficace nella terapia di mantenimento (con inibitore nucleosidico della transcriptasi inversa).

Si tratta si uno studio clinico multicentrico che mira a testare l’efficacità di una terapia di mantenimento con dolutergavir ed emtricitabina in biterapia in pazienti volontari in soppressione virologica. L’obbiettivo è dimostrare che questa terapia semplificata di mantenimento è non inferiore, in termini di soppressione virologica, a una terapia antiretrovirale standard e che un follow-up semplificato è benefico in termini di costi, accettabilità, sicurezza et qualità di vita per i pazienti rispetto al follow-up standard.

180 partecipanti saranno reclutati principalmente all’interno dello studio svizzero della coorte. Questi pazienti avranno una carica virale indetettabile da almeno 24 settimane, in terapia antiretrovirale convenzionale, senza precedenti di fallimenti della terapia antiretrovirale. I partecipanti saranno randomizzati per ricevere una biterapia di dolutegravir et emtricitabina o per proseguire la loro terapia standard; una seconda randomizzazione li assegnera a un follow-up semplificato o a un follow-up standard. Tutti i pazienti saranno visitati alla settimana 48 con frequenti visite di verifica dell’adesione terapeutica e della carica virale ogni 6 settimane durante il primo trimestre et poi con frequenza trimestrale. Una volta finita la settimana 48 dello studio, i pazienti potranno dare il loro consenso ad estendere il periodo di monitoraggio post-studio a 3 anni con una visita all’anno.

Questo studio si vuole generalizzabile poiché noi contiamo dimostrare che una terapia di mantenimento semplificata potrebbe essere ben tollerata e disponibile anche nelle regioni con risorse limitate.

Con un follow-up della durata di 144 settimane, speriamo rispondere alla domanda relativa all’efficacità di dolutegravir ed emtricitabina in biterapia con una potenza sufficiente per avvicinarci al contesto della vita reale.

Abbreviations

Provide a list of abbreviations used on the protocol

ACTG	AIDS Clinical Trial Group
AE	Adverse Event
ART	Antiretroviral Therapy
ASR	Annual Safety Report
CA	Competent Authority (e.g. Swissmedic)
CEC	Competent Ethics Committee
CK	Creatine Kinase
CNS	Central Nervous System
CRF	Case Report Form
ClinO	Ordinance on Clinical Trials in Human Research *(in German: KlinV, in French: OClin)*
CRA	Clinical Research Associate
CRC	Clinical Research Centre
eCRF	Electronic Case Report Form
EDC	Electronic Data Capture
CTCAE	Common terminology criteria for adverse events
CTU	Clinical Research Centre
DSMB	Data Safety and Monitoring Board
DSUR	Development safety update report
DTG	Dolutegravir
EACS	European AIDS Clinical Society
EMA	European Medicines Agency
FDA	Food and Drug Administration, USA
FOPH	Federal Office of Public Health's
FTC	Emtricitabine
GCP	Good Clinical Practice
IB	Investigator's Brochure
Ho	Null hypothesis
H1	Alternative hypothesis
HFG	Humanforschungsgesetz (Law on human research)
HIV	Human Immunodeficiency Virus
HMG	Heilmittelgesetz
HRA	Federal Act on Research involving Human Beings
HUG	Geneva University Hospital
ICH	International Conference on Harmonisation
IMP	Investigational Medicinal Product

IIT	Investigator-initiated Trial
INSTI	Integrase Strand Transfer Inhibitor
ISO	International Organisation for Standardisation
ITT	Intention to treat
KlinV	Verordnung über klinische Versuche in der Humanforschung *(in English: ClinO, in French OClin)*
LPTh	Loi sur les produits thérapeutiques
LRH	Loi fédérale relative à la recherche sur l'être humain
MD	Medical Device
NI	Non-Inferiority
NNRTI	Non-nucleoside Reverse Transcriptase Inhibitor
NRTI	Nucleoside Reverse-Transcriptase Inhibitor
OClin	Ordonnance sur les essais cliniques dans le cadre de la recherche sur l'être humain *(in German : KlinV, in English : ClinO)*
PCR	Polymerase Chain Reaction
PBMC	Peripheral Blood Mononuclear Cells
PI	Principal Investigator
PP	Per Protocol
QALY	Quality-adjusted life years
RIL	Rilpivirine
SAE	Serious Adverse Event
SDV	Source Data Verification
SF-12	Short Form 12-item Questionnaire
SHCS	Swiss HIV Cohort
SMT	Simplified Maintenance Trial
SOP	Standard Operating Procedure
SPC	Summary of product characteristics
SUSAR	Suspected Unexpected Serious Adverse Reaction
TLOVR	Time to Loss of Virological Response
TMF	Trial Master File
WHO	World Health Organization
3TC	Lamivudine

Study schedule

	Screening	Year 1					
	-4 weeks to -1 week	Day 0	Week 6	Week 12	Week 24	Week 36	Week 48
Eligibility screen [1]	X	X					
Written informed consent	X						
Demographics	X						
HIV-related data and medical history [2]		X					
Concomitant medications [3]		X	X	X	X	X	X
Full physical examination by physician [4]		X			X		X
Allocation (double randomization)		X					
Start DTG + FTC OR remain on standard cART		X					
Start patient-centered monitoring OR remain on standard monitoring		X					
Adherence check		X	X	X	X	X	X
Adverse events check		X	X	X	X	X	X
Drug distribution		X	X	X	X	X	X
HIV-RNA in plasma + sample storage	X[5]	X[β]	X[β]	X[β]	X[β]	X[β]	X[β]
HIV-DNA in plasma (in batch)		X					X
CD4 count*	X			X*	X*	X*	X
Full blood count*	X			X*	X*	X*	X

Columns with purple background represent visits which need to be performed at study site

[1] At screening visit: patient chart review for exclusion and inclusion criteria. At day 0: review of laboratory assessments made at the screening visit and completion of the eligibility checklist.
[2] At day 0: HIV-1 infection, date of HIV-1 diagnosis, years of HIV-1 suppression, nadir CD4 count, zenith HIV-1 RNA, previous genotype, ongoing hepatitis B and C, other co-morbidities. All other visits: updated medical history including changes or additions to diagnoses, diseases and medications will be done in the frame of the concomitant medication and the adverse event check.
[3] Prohibited: carbamazepine, oxcarbamazepine, phenytoin, phenobarbital, St John's wort and rifampicin.
[4] Full physical examination by study nurse & medical doctor: vital signs, anthropometric measurements, cardiovascular, respiratory and gastrointestinal systems; central nervous and skin systems as deemed necessary.
[5] No sample storage on screening visit

Safety serum chemistry [6]*	**X**		**¥µ**	**X*µ**	**X*µ**	**X*µ**	**X**
Urine Beta HCG pregnancy test in women with childbearing potential	**X**				**X**		**X**
HIV RNA in plasma and sample storage		**X**	**(at any time if HIV-RNA is ≥ 100 copies)**				
Genotypic resistance test (real time) and pharmacogenetic analysis (in batch)			**(at any time if HIV-RNA is ≥ 100 copies on two consecutive measurements)**				
DTG and FTC plasma concentration			**(at any time if HIV-RNA is ≥ 100 copies, if twice consecutively only at first time)**				
Health-related quality of life questionnaire PROQOL-HIV<br>Visual analog scale		**X**<br>**X**		**X**<br>**X**			**X**<br>**X**
Assess willing for change of monitoring option / satisfaction if patient randomised in the patient-centered monitoring arm				**X**	**X**	**X**	**X**
Health related costs assessment							**X**

[6] Safety serum chemistry includes CK, creatinine, ASAT, ALAT, gamma-GT, alkaline phosphatases; glucose and lipid profile.
[β] Patients with HIV-RNA between 20 and 99 copies/ml will benefit from another HIV-RNA measurement after 6 weeks (± 5 days). Patients with HIV-RNA ≥ 100 copies/ml will benefit from another HIV-RNA measurement after 2 weeks (± 5 days).
* CD4-count, full blood count and safety serum chemistry will be performed on a 3-monthly basis for patients randomized in the standard monitoring arm. Patients randomized in the patient-centered monitoring arm will benefit from these routine tests on a yearly basis (i.e. at day 0 and week 48).
[¥] Patients new to DTG will benefit from CK measurement and from hepatic safety serum tests, i.e. ASAT, ALAT, gamma-GT, alkaline phosphatases, at week 6 whatever monitoring arm.
[µ] Diabetic patients under concomitant metformine and dolutegravir will benefit from blood glucose and creatinine measurements at weeks 6, 12, 24 and 36 whatever monitoring arm.

1. STUDY ADMINISTRATIVE STRUCTURE

1.1 Sponsor, Sponsor-Investigator

Professor Alexandra CALMY, Médecin adjointe agrégée, Director, HIV Unit, Division of Infectious Diseases, Geneva University Hospitals, Geneva; clinical study leader.

E-mail: Alexandra.calmy@hcuge.ch

Phone: 022 372 98 12

The Sponsor-investigator will oversee all activities within this protocol in regular meetings with the trial team, the trial coordinators and the sites (teleconferences, physical meetings and email). The Sponsor-investigator will setup contracts with the participating sites to determine the sites' and the sponsor's role in the study (i.e. data collection, reporting and reimbursement). Major tasks such as protocol writing, site monitoring visits, data management, statistical analysis, interpretation of the data, writing of the report will be conducted in collaboration with the trial team and the Sponsor's delegates. Contracts will be setup with the Clinical Trial Units where necessary.

1.2 Principal Investigator(s)

Principal Investigators:

- Dr Delphine Sculier, MD, MSc, Avenue de Champel 6, 1206 Genève, Switzerland, phone : +41 (0)79 810 74 76; Delphine.sculier@hin.ch
- Dr Gilles Wandeler, MD, MSc, Department of Infectious Diseases, Bern University Hospital, University of Bern, 3010 Bern, Switzerland, phone : +41 78 775 85 33 ; gilles.wandeler@ispm.unibe.ch

Site-Principal investigators:

- Dr Delphine Sculier, MD, MSc, Avenue de Champel 6, 1206 Genève, Switzerland, phone : +41 (0)79 810 74 76; delphine.sculier@hin.ch
- Dr Gilles Wandeler, MD, MSc, Department of Infectious Diseases, **Bern** University Hospital, Univ**ersi**ty of Bern, Finkenhubelweg 11, 3012 Bern, Switzerland, phone : +41 78 775 85 33 ; gilles.wandeler@ispm.unibe.ch
- Prof Enos Bernasconi, MD, PhD, Service of Infectious Diseases, **Lugano** Regional Hospital, Via Tesserte 46, CH-6903Lugano, Switzerland, phone: +41 91 811 60 22 Enos.Bernasconi@eoc.ch
- Dr Dominique Braun, MD, Department of Infectious Diseases and Hospital Epidemiology, University Hospital of **Zurich**, Rämistrasse 100, 8091 Zürich, Zurich Switzerland, phone : +41 44 255 91 96, Dominique.Braun@usz.ch
- Dr Matthias Cavassini, MD, PD, Department of Infectious Diseases, University Hospitals of **Lausanne**, Rue du Bugnon, 46, 1011 Lausanne, Switzerland, phone: +41 21 314 1022i Matthias Cavassini@chuv.ch
- Dr Marcel Stoeckle: MD, Department of Infectious Diseases and Hospital Epidemiology, University Hospital of **Basel**, Spitalstrasse 21, 4056 Basel, Basel, Switzerland, Phone: +41 61 265 50 62, Marcel.Stoeckle@usb.ch
- Dr. Patrick Schmid, MD, Division of Infectious Diseases and Hospital Epidemiology, Kantonspital **St.Gallen**, Rorschacher Str. 95, 9007 St. Gallen, Switzerland, phone: +41 71 494 10 21, Patrick.schmid @kssg.ch

Co-investigators:

- Prof Huldrych Günthard, MD, PhD, Division of Infectious Diseases and Hospital Epidemiology, University Hospital **Zurich**, University of Zurich, Winterhurerstrasse 190, CH-8057 Zurich, Switzerland, phone: +41 44 255 34 50, huldrych.guenthard@usz.ch
- Dr Karin Metzner, PhD, Division of Infectious Diseases and Hospital Epidemiology, Departement

of Medicine, University Hospitals Zurich, Rämistrasse 199, U WEST 1, 8006 Zurich, Switzerland, phone: +41 44 255 30 29, karin.metzner@usz.ch
- Prof Matthias Egger, MD, PhD, MSc FFPH DTM&H, Institute for Social and Preventive Medecine, Finkenhubelweg 11, 3012 Bern; phone: +41 31 631 35 01, email: matthias.egger@ispm.unibe.ch
- Dr Jan Fehr, MD, PD, Department of Infectious Diseases and Hospital Epidemiology, University Hospital of Zurich, Rämistrasse 100, 8091 Zürich, Zurich, Switzerland, phone: +41 44 255 34 02, Jan.Fehr@usz.ch
- Mrs Sabine Yerly, MSc, Department of genetics and laboratories, Virology laboratory, rue Gabrielle-Perret-Gentil 4, 1211 Genève 14, Switzerland, Sabine.Yerly@hcuge.ch
- Prof Laurent Decosterd, PhD, Pharmacology laboratory, Clinical pharmacology departement, uniuversity of Lausanne, phone: +41 21 314 42 72, LaurentArthur.decosterd@chuv.ch
- Prof Pietro Vernazza, MD, PhD, Division of Infectious Diseases and Hospital Epidemiology, Kantonspital **St.Gallen**, Rorschacher Str. 95, 9007 St. Gallen, Switzerland, phone: +41714942631, Pietro.vernazza@kssg.ch

1.3 Methodologists and Statistician ("Biostatistician")

General methodologists:
- Dr Sven Trelle, PD, MD, Co-director, Clinical Trials Unit Bern, University of Bern, Finkenhubelweg 11, 3012 Bern; phone: +41 31 631 35 04, email: sven.trelle@ctu.unibe.ch
- Prof Matthias Egger Prof. Dr. med., MSc FFPH DTM&H, Institute for Social and Preventive Medecine, Finkenhubelweg 11, 3012 Bern; phone: +41 31 631 35 01, email: matthias.egger@ispm.unibe.ch

Statistician:
- Andreas Limacher, PhD, head of Statistics and Methodology, Clinical Trials Unit Bern, University of Bern, Finkenhubelweg 11, 3012 Bern; phone: +41 31 631 35 10, email: andreas.limacher@ctu.unibe.ch

1.4 Laboratory

Virology laboratory Geneva HUG

Mrs Sabine Yerly, MSc, Department of genetics and laboratories, Virology laboratory, rue Gabrielle-Perret-Gentil 4, 1211 Genève 14, Switzerland, Sabine.Yerly@hcuge.ch,

Pharmacolocy laboratory Lausanne CHUV

Prof Laurent Decosterd, PhD, Clinical pharmacology department, University Hospital of Lausanne, LaurentArthur.decosterd@chuv.ch

Division of Infectious Diseases and Hospital Epidemiology USZ

Dr Karin Metzner, PhD, Division of Infectious Diseases and Hospital Epidemiology, Departement of Medicine, University Hospitals Zurich, Rämistrasse 199, U WEST 1, 8006 Zurich, Switzerland, karin.metzner@usz.ch

All participating sites will be using their local laboratories for virology, immunology, haematology and chemistry assessments. The laboratories' postal addresses, certifications and normal values will be filed in the investigator files of the respective sites.

1.5 Monitoring institution

Clinical Research Unit (CTU) of Geneva University Hospitals will be responsible for developing a central monitoring plan that will be followed in the seven study sites.

1.6 Data Safety Monitoring Committee

An independent Data Safety and Monitoring Board (DSMB) will be set up to monitor the safety and efficacy of the study interventions in participants enrolled in the trial. The DSMB will be chaired by Jose Ramon Arribas Lopez, and will include *Yasdan Yasdanpanah (clinical epidemiology, pharmaco-economics), Valérie Journot (statistician), Bart Rijnders (clinical medicine) and Samia Hurst (bioethics)*. A conflict of interest statement will be completed by all members. Frequency of data reporting to the DSMB, frequency of DSMB meetings and stopping rules are described in section 11.3.

1.7 Any other relevant Committee, Person, Organization, Institution

1.7.1. Study Scientific Committee:

- Prof Matthias Egger Prof. Dr. med., MSc FFPH DTM&H, Institute for Social and Preventive Medecine, Finkenhubelweg 11, 3012 Bern; phone: +41 31 631 35 01, email: matthias.egger@ispm.unibe.ch
- Prof Alexandra Calmy, MD, PhD, D, Medical specialties department, Infectious diseases consultation, University Hospitals of Geneva, rue Gabrielle-Perret-Gentil 4, 1211 Genève 14, Switzerland, phone: +41 22 372 98 12, Alexandra.calmy@hcuge.ch
- Dr Delphine Sculier, MD, MSc, Avenue de Champel 6, 1206 Genève, Switzerland, phone: +41 79 810 74 76, Delphine.sculier@hin.ch
- Dr Gilles Wandeler, MD MSc, Department of Infectious Diseases, Bern University Hospital, University of Bern, 3010 Bern, Switzerland, phone : +41 XXX ; gilles.wandeler@ispm.unibe.ch
- Dr Marcel Stoeckle, MD, Department of Infectious Diseases and Hospital Epidemiology, University Hospital of Basel, Spitalstrasse 21, 4056 Basel, Basel, Switzerland, Phone: +41 61 265 50 62, Marcel.stoeckle@usb.ch
- Dr Jan Fehr, PD Dr. med. Department of Infectious Diseases and Hospital Epidemiology, University Hospital of Zurich, Rämistrasse 100, 8091 Zürich, Zurich, Switzerland, phone: +41 44 255 34 02, Jan.fehr@usz.ch
- Prof Enos Bernasconi, Dr.med., Serviceof Infectious Diseases, Lugano Regional Hospital, Via Tesserete 46, CH-6903 Lugano, Switzerland, phone: +41 91 811 60, Enos.bernasconi@eoc.ch
- Dr Dominique Braun, Dr. med, Department of Infectious Diseases and Hospital Epidemiology, University Hospital of Zurich, Rämistrasse 100, 8091 Zürich, Zurich Switzerland, phone : +41 44 255 91 96, Dominique.braun@usz.ch
- Prof Pietro Vernazza, Division of Infectious Diseases and Hospital Epidemiology, Kantonspital St.Gallen, Rorschacher Str. 95, 9007 St. Gallen, Switzerland, phone: +41714942631, Pietro.vernazza@kssg.ch
- Dr Matthias Cavassini, MD, PD, Department of Infectious Diseases, University Hospitals of Lausanne, Rue du Bugnon, 46, 1011 Lausanne, Switzerland, phone: +41 21 314 10 22, Matthias.Cavassini@chuv.ch
- Prof Huldrych Günthard, MD, PhD, Division of Infectious Diseases and Hospital Epidemiology, University Hospital Zurich, University of Zurich, Winterhurerstrasse 190, CH-8057 Zurich, Switzerland, phone: +41 44 255 34 50, Huldrych.guenthard@usz.ch
- Dr. Patrick Schmid, MD, Division of Infectious Diseases and Hospital Epidemiology, Kantonspital St.Gallen, Rorschacher Str. 95, 9007 St. Gallen, Switzerland, phone: +41714941021, Patrick.schmid @kssg.ch
- Dr Karin Metzner, PhD, Division of Infectious Diseases and Hospital Epidemiology, Departement of Medicine, University Hospitals Zurich, Rämistrasse 199, U WEST 1, 8006 Zurich, Switzerland, phone: +41 44 255 30 29, Karin.metzner@usz.ch
- Mrs Sabine Yerly, MSc, Department of genetics and laboratories, Virology laboratory, rue Gabrielle-Perret-Gentil 4, 1211 Genève 14, Switzerland, Sabine.Yerly@hcuge.ch
- Prof Laurent Decosterd, PhD, Pharmacology laboratory, Clinical pharmacology departement, uniuversity of Lausanne, phone: +41 21 314 42 72, LaurentArthur.decosterd@chuv.ch
- Dr Sven Trelle, MD, Co-director, Clinical Trials Unit Bern, University of Bern, Finkenhubelweg 11, 3012 Bern; phone: +41 31 631 35 04, email: sven.trelle@ctu.unibe.ch

External persons might be invited to join the Study Scientific Committee meeting.

1.7.2. Trial Team Coordinators at the coordinating centre Geneva

The study will be coordinated under the direct supervision of the sponsor-investigator by the following study physicians and clinical research associates at the research team of the HIV unit at Geneva

University Hospitals (Geneva coordinating centre):

Dr Marta Buzzi, MD, Infectious diseases consultation, Geneva University Hospitals, Rue Gabrielle Perret-Gentil 4, 1211 Genève 14, Switzerland, phone: +41 22 3723351, email: marta.buzzi@hcuge.ch, will be coordinating medical issues, study safety, data review, report writing.

Dr Annalisa Marinosci, MD, Infectious diseases consultation, Geneva University Hospitals, Rue Gabrielle Perret-Gentil 4, 1211 Genève 14, Switzerland, phone: +41 22 37228809, email: annalisa.marinosci@hcuge.ch, will be coordinating medical issues, study safety, data review, report writing.

Ms. Tamara Da Silva, Clinical Research Associate at the Infectious diseases consultation, Geneva University Hospitals, Rue Gabrielle Perret-Gentil 4, 1211 Genève 14, Switzerland, phone: +41 22 3729812, email: Tamara.dasilva@hcuge.ch, will be coordinating essential documents set up, SOPs, regulatory issues, monitoring, document retention as well as day to day contact with the sites.

Ms. Charlotte Barbieux, Clinical Research Associate at the Infectious diseases consultation, Geneva University Hospitals, Rue Gabrielle Perret-Gentil 4, 1211 Genève 14, Switzerland, phone: +41 22 3729804, email: charlotte.barbieux@hcuge.ch, will be coordinating essential documents set up, SOPs, regulatory issues, monitoring, document retention as well as day to day contact with the sites.

Other clinical research associates present at the HIV research team in Geneva are not excluded.

Roles and responsibilities of the coordinators are defined by the sponsor-investigator in the terms of reference of each employee.

2. ETHICAL AND REGULATORY ASPECTS

The decision of the CEC concerning the conduct of the study will be made in writing to the Sponsor-Investigator before commencement of this study. The clinical study can only begin once approval from all required authorities has been received. Any additional requirements imposed by the authorities shall be implemented.

2.1 Study registration

The study will be registered in the www.clinicaltrials.gov registry and in the Swiss Federal Complementary Database.

2.2 Categorisation of study

This study is a category A study because the interventional product is used as a dual therapy in combination with FTC in this protocol, i.e. in association with other antiretroviral agents as indicated in the product information.

2.3 Competent Ethics Committee (CEC)

The responsible investigator ensures that approval from an appropriately constituted Competent Ethics Committee (CEC) is sought for the clinical study. The Sponsor-Investigator asks to the Ethics Committee document needed for this aim and make tham available to the related study site.

It is the investigators duty to report all changes in the research activity and all unanticipated problems involving risks to humans; including in case of planned or premature study end and the final report to the ethics committee within the required time frames. Reporting time frames for adverse events are mentioned in chapter 10 of this protocol.

No changes are made to the protocol without prior Sponsor and CEC approval, except where necessary to eliminate apparent immediate hazards to study participants.

Premature study end or interruption of the study is reported within 15 days. The regular end of the study is reported to the CEC within 90 days, the final study report shall be submitted within one year after study end. Amendments are reported according to chapter 2.10.

2.4 Competent Authorities (CA)

The study is a category A study. Therefore, approval from the competent authority (Swissmedic) is not necessary before the start of the clinical trial.

2.5 Ethical Conduct of the Study

The study will be carried out in accordance to the protocol and with principles enunciated in the current version of the Declaration of Helsinki, the guidelines of Good Clinical Practice (GCP) issued by ICH, the Swiss Law and Swiss regulatory authority's requirements. The CEC will receive annual safety and interim reports and be informed about study stop/end in agreement with local requirements.

2.6 Declaration of interest

The Sponsor-investigator has received financial support from the Swiss National Fund for Science for the present study; the sponsor-investigator, all investigators and authors of the final manuscript will have no financial relationships with any organisations that might have an interest in the present study; no other relationships or activities that could appear to influence the results of the study.

2.7 Patient Information and Informed Consent

The investigators will explain to each participant the nature of the study, its purpose, the procedures involved, the expected duration, the potential risks and benefits and any discomfort it may entail. Each participant will be informed that the participation in the study is voluntary and that he/she may withdraw from the study at any time and that withdrawal of consent will not affect his/her subsequent medical assistance and treatment.

The participant must be informed that his/her medical records may be examined by authorised individuals other than their treating physician.

All participants for the study will be provided a participant information sheet and a consent form describing the study and providing sufficient information for participant to make an informed decision about their participation in the study. A time frame of preferably 24 hours will be given to the participant to decide whether to participate or not.

The patient information and consent form (appendix 1) will be submitted to the CEC to be reviewed and approved. The formal consent of a participant, using the approved consent form, must be obtained before the participant is submitted to any study procedure.

The participant should read and consider the statement before signing and dating the informed consent form, and should be given a copy of the signed document. The consent form must also be signed and dated by the medical investigator and it will be retained as part of the study records. Medical investigator can delegate the signature of the consent form to a study nurse via the delegation log. In this case the medical investigator must countersign the consent form before the randomisation during the baseline visit.

2.8 Participant privacy and confidentiality

The investigator affirms and upholds the principle of the participant's right to privacy and that they shall comply with applicable privacy laws. Especially, anonymity of the participants shall be guaranteed when presenting the data at scientific meetings or publishing them in scientific journals.

Individual subject medical information obtained as a result of this study is considered confidential and disclosure to third parties is prohibited. Subject confidentiality will be further ensured by utilising subject identification code numbers to correspond to treatment data in the computer files.

For data verification purposes, authorised representatives of the Sponsor-Investigator, a competent authority (e.g. Swissmedic), or an ethics committee may require direct access to parts of the medical records relevant to the study, including participants' medical history.

2.9 Early termination of the study

The Sponsor-Investigator may terminate the study prematurely according to certain circumstances, for example:

- ethical concerns,
- insufficient participant recruitment,
- when the safety of the participants is doubtful or at risk, respectively,
- alterations in accepted clinical practice that make the continuation of a clinical trial unwise,
- early evidence of benefit or harm of the experimental intervention.

2.10 Protocol amendments

Substantial amendments are only implemented after approval of the CEC respectively.

Under emergency circumstances, deviations from the protocol to protect the rights, safety and well-being of human subjects may proceed without prior approval of the sponsor and the CEC. Such deviations shall be documented and reported to the sponsor and the CEC as relevant according to the procedure described in the related Standard Operating Procedure. All Non-substantial amendments are communicated to the CEC within the Annual Safety Report (ASR).

3. BACKGROUND AND RATIONALE

3.1 Background and Rationale

Current European HIV treatment (EACS) guidelines recommend a combination of three active drugs for the initiation and maintenance of ART. Combinations usually include two nucleoside reverse transcriptase inhibitors (NRTIs) plus a non-nucleoside reverse transcriptase inhibitor (NNRTI), or a protease inhibitor (PI), or an integrase inhibitor (INSTI). The cost of a full standard triple treatment (drugs only) in Switzerland ranges from CHF 14,000 to CHF 22,000 per year (1) .

Simplified maintenance therapy in patients on suppressive ART aims to limit toxicities, support retention in care, and reduce costs to the healthcare system (2, 3) (Figure 1).

Figure 1: HIV continuum of care, from induction to maintenance regimens

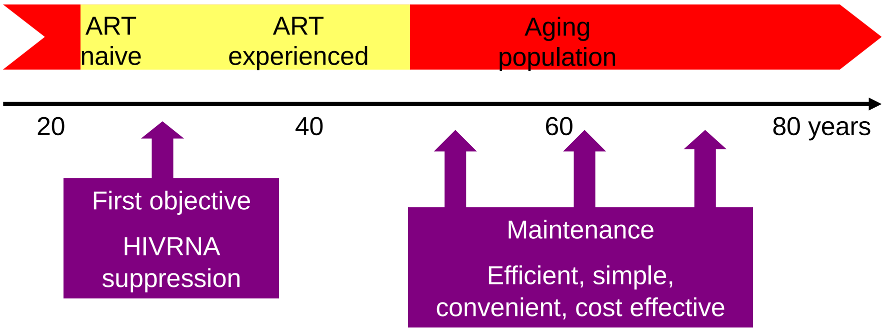

(courtesy of Pietro Vernazza)

Current simplified maintenance regimens are NRTI-sparing regimens, mainly boosted PI-based mono and dual therapies (4-6). Eleven years after the first boosted PI monotherapy trial (7), revisiting this strategy is critical. In 2015, a PI-based mono-maintenance regimen has still not been widely adopted due to suboptimal virological control, or the lack of the necessary infrastructure to monitor such regimens in resource-limited settings (2). The PROTEA trial also showed less efficacy of PI monotherapy in patients with nadir CD4 less than 200 cells/µl (8). Other possible maintenance regimen includes cabotegravir plus rilpivirine dual therapy as a phase II, industry-sponsored trial showed excellent antiviral activity compared to efavirenz plus dual-NRTIs until the end of week 96 (9). The very good tolerability, high convenience, lack of drug-drug interactions, high potency, and distinctive resistance profile suggest that INSTI-based therapy could be an acceptable and efficient alternative to PIs in simplified maintenance therapy as a bitherapy (2).

Dolutegravir efficacy data in antiretroviral drug combinations

The SINGLE, FLAMINGO and SPRING 2 randomized controlled trials showed the superiority of DTG at week 48 compared to efavirenz (88% of patients achieving undetectable viral load compared to 81% respectively, p=0.003) or ritonavir boosted darunavir (90% vs. 83%, p=0.025), and its non-inferiority to raltegravir (RAL) (88% vs. 85%) in antiretroviral drug combinations among ART-naïve HIV-infected patients(10-12). In these trials, no patient developed mutations associated with resistance to integrase inhibitors or accompanying NRTIs. This absence of development of resistance has never been seen in past trials assessing the efficacy of first line ART regimens with NNRTIs or PIs(13). The DTG resistance pattern is driven by the R263K mutation which also confers a loss in viral fitness, potentially explaining the absence of detectable mutations by conventional assays in ART-naïve patients receiving DTG(14).

DTG was found superior to raltegravir among treatment-experienced patients with resistance to at least 2 classes of antiretroviral drugs, excluding integrase inhibitors, with 71% patients on DTG achieving viral suppression at week 48 compared to 64% in the raltegravir arm (15). In patients with raltegravir or elvitegravir resistant HIV infection, DTG may retain antiviral activity at the dose of 50 mg twice daily(16, 17) as DTG resistance patterns are different to resistance patterns to raltegravir or elvitegravir(14). However, cross-resistance mutations such as the combination of Q148H/R/K with G140S/A may decrease dolutegravir susceptibility (18).

HIV DNA reservoirs

A challenge in HIV treatment is the existence of a HIV-DNA reservoir made of latently infected CD4 T-cells circulating in the peripheral blood or disseminated in lymphoid organs and associated tissues(19, 20). Sub-optimal drug penetration in tissues such as the genital tract or the brain may allow for ongoing replication in body compartments even with undetectable plasma viral load(21). The HIV-DNA reservoir establishes itself very early in the course of HIV infection(22), and is a strong predictor of HIV disease progression(23) as well as a risk factor for neurocognitive disorders(24). High level of HIV-DNA was predictive of virological rebound during ART interruptions(25). Some studies found that RAL decreased the HIV-DNA reservoir in latently infected peripheral T-cells when used in an intensification ART strategy(26) or when part of a conventional triple ART regimen(27).

The reservoir is measured by the total cell-associated HIV-DNA as the frequency of infected cells per million in peripheral blood mononuclear cells (PBMC)(20). Boulassel et al demonstrated that the CD4 cell nadir independently predicted the size of the HIV reservoir after prolonged therapy (28). In the current trial we will measure proviral DNA from PBMCs by using a novel digital droplet PCR based assay as developed in the laboratory of H. Günthard / K. Metzner at the University Hospital Zurich for the HIV.X project “Deciphering Host-Virus Interactions to Cure HIV” (29). We will also assess if proviral DNA, and nadir CD4 count, could predict virological failure.

Simlified maintenance therapy has the advantage to reduce common NRTI and NNRTI associated side-effects, such as renal and central nervous system toxicities mainly due to tenofovir and efavirenz respectively(30), which in turn will reduce costs to the healthcare system (31). Regimen simplification is also considered critical to further scale-up of treatment, particularly in low- and middle-income settings, and to support retention in care (3). The World Health Organization favours a simple regimen with the following characteristics: minimal risk of failure; efficacy and tolerability; robustness and forgiveness; no overlapping resistance in treatment sequencing; and convenience (3). Simplified DTG-based therapy seems to meet these criteria. Our primary objective is to assess the efficacy of simplified maintenance DTG- based dual therapy, together with FTC, in patients followed within the community research network of the Swiss HIV Cohort Study (SHCS).

Optimization of clinical and biological treatment monitoring

Maintenance therapy does not only refer to drug optimization. Costs are mainly shared among the direct drug costs, biological monitoring, and medical (and paramedical) consultations(32). We consider that with newer more potent antiretroviral drugs, which are also better tolerated, there is room for resource optimization. The use of routine, frequent CD4 cell measurements in the monitoring of HIV-infected patients on treatment is an excellent example. This raises the issue as to whether regular and frequent CD4+ cell counts measurements are still of benefit for monitoring treatment success in these patients when access to HIV-RNA measurement is guaranteed. Thus, while a CD4 cell count at baseline continues to be important for initial clinical management decisions, once ART is initiated and patients have stabilized on treatment, the additional value of CD4 testing is questionable. Indeed, a recent meta-analysis and literature review supported the reduction in the frequency or stopping routine CD4 monitoring for patients who are immunologically stable on ART in settings where routine viral load monitoring is provided (33, 34). Based on previous randomized trials among adults and children (35, 36), we anticipate that routine lipid, renal, and complete blood count testing may not be helpful in the management of patients on long-term therapy.

To our knowledge, optimized monitoring strategies have only been tested in resource-limited settings where human and financial resources are scarce compared to the growing number of HIV patients in care. Several strategies have demonstrated positive results. The STRATALL trial demonstrated that the clinical follow-up of HIV patients commencing cART was non-inferior to biological monitoring with 6-monthly CD4 count and HIV viral load measurements, at a time when generic HIV viral load assays were not available (37, 38). A Cochrane systematic review of 10 studies, all conducted in Africa and including randomized controlled trials (39, 40), showed that task-shifting of ART management from doctors to trained and supported nurses did not reduce the quality of care and potentially decreased the number of patients lost to follow-up. Ahn et al have shown that annual CD4 monitoring was sufficient in virologically suppressed patients with higher baseline CD4 count ≥250 cells/mm^3 in the Asia-Pacific region (41).

Seventy percent of HIV-infected patients in Switzerland are integrated into the work force and most are able to work full time (42). In this situation, a patient-centered monitoring may be more suitable in the long-term. As a second objective, we propose to assess if a patient-centered monitoring that offers alternative options for the delivery of study visits, laboratory tests and drugs is cost-saving (first goal) and acceptable (second goal) , without jeopardizing safety, compared to routine monitoring.

3.2 Investigational Product (treatment) and Indication

DTG is a second-generation INSTI that can be administered once daily without the need for pharmacokinetic boosting, irrespective of food intake, and with very few drug-drug interactions. Following a dose of 50 mg, it has a plasma concentration 24 hours after that is 19 times higher than the *in vitro* protein-adjusted IC90 (concentration inhibiting 90% of *in vitro* viral replication) of 64 ng/ml, thus providing a significant buffer in the case of reduced concentrations due to late or missed doses, reduced absorption, or drug interactions (10, 43). *In vivo* trough concentration values are 25 times higher than the *in vitro* threshold and its long half-life of 12 hours also contributes to the higher efficacy observed with DTG (43). However, plasma concentrations of antiretroviral drugs may be influenced by drug-metabolizing enzymes or drug transporters, leading either to insufficient levels of the drugs with the risk of virological failure, either to higher plasma levels associated with side effects (44). For example, large interindividuel variation in plasma concentration of raltegravir was reported (45) and raltegravir was found to be a substrate of efflux transporters expressed in the intestines (46, 47). Efflux transporters with altered function could also potentially impact dolutegravir plasma concentration.

Prolonged and more stable binding to the integrase-DNA complex compared to first generation integrase inhibitors, raltegravir or elvitegravir, with a longer dissociative half-life (71 hours compared with 8.8 hours for raltegravir and 2.7 hours for elvitegravir) is thought to add to its higher barrier to resistance (48). The DTG resistance pattern is driven by the mutation R263K, which confers low-grade resistance to the drug, and defers from the three genetic pathways involving integrase residues Q148, N155, and Y143 to the first-generation INSTI (43). When associated with H51Y and/or G118R mutations, the mutation R263K decreases viral replication capacity and impairs the emergence of resistance against reverse transcriptase inhibitors (13, 14). This loss in viral fitness may explain the absence of detectable mutations by conventional assays in naïve patients receiving DTG (14). Single initial mutations to DTG in salvage trials do not result in high-level resistance to the drug, suggesting a high genetic barrier at least similar to the one of boosted PI (13). However, recent data from the SAILING trial and an observational DTG monotherapy maintenance study have shown the development of INSTI mutations including N155H or Q148R pathways (15, 49, 50). Cross-resistance mutations such as the combination of Q148H/R/K with G140S/A may also decrease dolutegravir susceptibility in patients failing raltegravir salvage regimens (18).

DTG is well tolerated with only 2% to 3% of patients discontinuing the drug for adverse events in phase III trials. Frequent side-effects include sleep disturbance, gastro-intestinal complaints, headache, and respiratory tract complaints; most were grades 1 or 2 in clinical trials (13, 14). No life threathening adverse events were observed. Rates of psychiatric adverse events, i.e. insomnia, anxiety, depression or suicidality, were low in treatment-naïve phase III/IIb trials of DTG with frequency of withdrawal as low as 5% (51). However, observational studies have reported higher rates of central nervous system side effects (up to 20%) in treatment-naïve or -experienced patients receiving DTG (52, 53) with discontinuation rates due to central nervous system adverse events from 0.6% to 3.4% (52, 54-56). A retrospective cohort study in the Netherlands reported up to 13.7% of discontinuation rate due to adverse event on DTG (57) and another in Germany found that women and older patients were more at risk of discontinuating DTG for neuropsychiatric side effects (58). DTG is also lipid neutral with fewer elevations of low-density lipoprotein of grade 2 or higher compared to efavirenz or boosted darunavir in the SINGLE and FLAMINGO trials (12, 59). However, there are no data on the effect of DTG on body fat composition and the associated cardiovascular risk or bone mass density, which are growing concerns in our aging HIV population.

Drug interactions are very few (43), another advantage of the compound. DTG is a substrate of UGT1A1, CYP3A4, UGT1A3, and UGT1A9. Only 1% of potential drug-drug interactions are considered to be at risk (Figure 2). DTG should be used at the dose of 50mg twice daily with rifampicin, a potent inducer of UGT1A1 and CYP3A4. Co-administration with other inducers of UGT1A1 and CYP3A4 such as carbamazepin, oxcarbamazepin, phenytoin, phenobarbital and St. John's wort should be avoided. As DTG inhibits the renal transporter OCT2, it is contraindicated with the antiarrhythmic dofetelide (which is not authorized in Switzerland) and caution is needed when co-administered with metformin. DTG should be taken with food when administered with calcium or iron supplementation or vitamins intake as well as with magnesium or aluminium-containing antiacids (14).

<u>Figure 2:</u> Drug-drug interactions in the INSTI drug classes

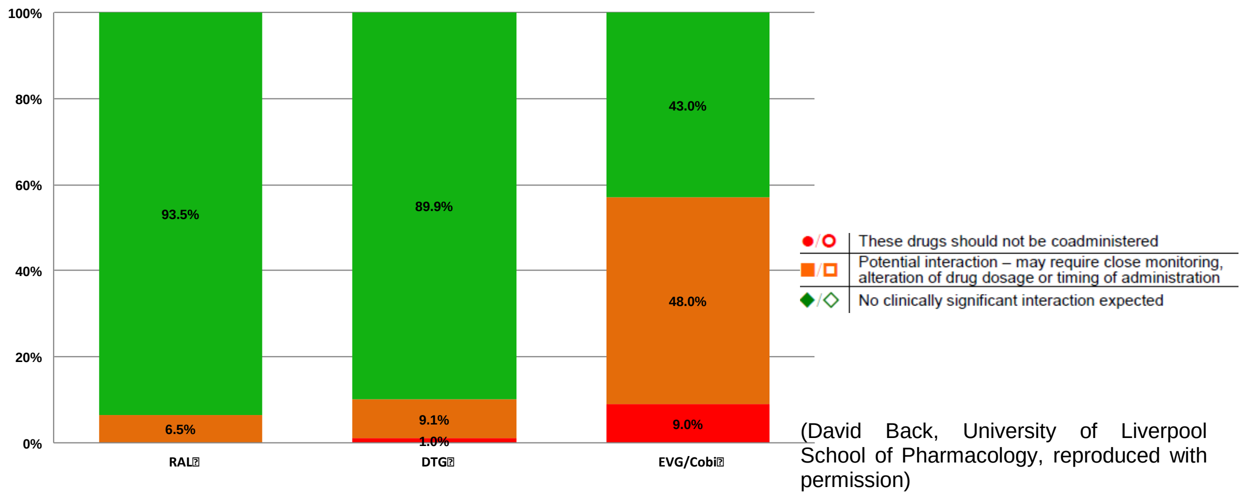

(David Back, University of Liverpool School of Pharmacology, reproduced with permission)

Abbreviations: RAL, raltegravir; DTG, dolutegravir; EVG/COBI, elvitegravir and cobicistat in a co-formulation

Untill July 2017, there was no data from well-controlled studies evaluating the use of DTG-based regimens in pregnant women. However, pharmacokinetics of maternal dolutegravir were assessed as part of the IMPAACT 1026 study, a phase 4 study evaluating the use of ARV regimens in pregnant women in the Americas (60). The calculated DTG area under the concentration versus time curve (AUC) was 25-30% lower in the 2nd and 3rd trimesters compared to paired postpartum data but these differences were not statistically significant and the AUC was similar to historical adult controls (60). One maternal adverse event of increased hepatic tests was reported and possibly related to DTG. Four infants were born with congenital abnormalities but no relationship to ARV exposure during first trimester was reported (60).
Post-marketing surveillance up to January 16, 2016 reported a total of 74 pregnancies on DTG. Pregnancy outcomes were as followed: 18 live enfant with no apparent congenital anomaly, 2 live enfant with congenital anomaly, 13 spontanerous abortion with no apparent congenital anomaly, 1 spontaneous abortion with congenital anomaly, 1 still birth with no apparent congenital anomaly and 39 outcomes were ongoing or unknown (61).
Based on results shown in July 2017 in 845 pregnant women in Botzwana(62), dolutegravir-based triple antiretroviral regimen was accepted in the 9.0 EACS guideline also during pregnancy(63), and dolutegravir was announced as part of first line regimen in various low-income countries regardless of the childbearing potential (64).
However, on May 18, 2018, the WHO, the FDA, and the EMA diffused an advertisement regarding a safety signal on the use of dolutegravir in early pregnancy with a risk of neural tube defect in infant born to women using dolutegravir in early pregnancy. This advertisement was based on a non planned analysis of data from pregnant women using dolutegravir in pregnancy in Botswana: 4/426 women gave birth to infants with neural tube defects(65-67). Despite the safety signal regarding the use of DTG in early pregnancy, dolutegrvir is considered safe when used in late pregnancy(68).

Emtricitabine is a nucleoside reverse transcriptase inhibitor that has activity against both HIV and hepatitis B virus. It is very similar to lamivudine (3TC) with respect to activity, convenience, safety and resistance profile (69). However, a randomized trial of FTC versus 3TC short-term monotherapy showed a longer plasma half-life activity and a superior antiviral activity of FTC 200 mg once a day compared to 3TC (70). Finally, FTC is a pregnancy category B drug and therefore an acceptable medication to use during pregnancy (69).

3.3 Preclinical Evidence

Based on exhaustive OMS literature review (71), even if FTC is currently considered equivalent to 3TC, some pharmacological differences could be important in the clinical context of dual therapy. FTC has a longer half-life and a greater potency against HIV-1 compared to 3TC. FTC has superior inhibition of viral replication when combined with tenofovir (TDF) compared to 3TC+TDF combination and has a higher binding affinity for reverse transcriptase and lower affinity for mitochondrial DNA

polymerase compared to 3TC. Moreover, the development of the M184V/I mutation, which is the most common NRTI mutation, is associated to a greater extent with the use of a 3TC-rather than a FTC-containing regimen.

3.4 Clinical Evidence to Date

Dolutegravir efficacy data in maintenance monotherapy

DTG monotherapy as maintenance treatment in an ART experienced population was proved to be feasible, with a high rate of effectiveness over 24 weeks in three observational studies (49, 72, 73). *Katlama et al* enrolled 28 patients virologically controlled for 6.6 years with a median nadir CD4 count of 215 cells/mm^3. The proportion of patients with viral load <50 copies/ml at week 24 was 89% (25/28 patients). Three virological failures were reported: all occurred in patients previously exposed to first generation integrase inhibitors, and patients developed signature mutations for integrase inhibitors at week 24. Patients were however switched back to conventional triple ART regimen with virological success. The study by *Rojas et al* included 33 patients, virologically controlled for 8 years and with a mean nadir CD4 count of 229 cells/mm^3 (SD ±125); 97% of patients remained with an undetectable viral load at 24 weeks. One patient, previously failing on raltegravir, demonstrated virological failure with HIV-DNA genotypic resistance test showing a signature mutation for integrase inhibitor at week 24. *Oldenbuettel et al* enrolled 31 patients virologically suppressed for 24 weeks. One patient discontinued for adverse event (ongoing cough) and one presented with virological failure at week 24. This latest patient continued on DTG monotherapy for five more weeks; the genotypic resistance test at that time showed major INSTI mutations (73).

However, an academic-driven randomized controlled trial of DTG mono-maintenance therapy (the DOMONO trial - NCT02401828) in which 104 HIV virologically suppressed patients were enrolled to an immediate or delayed (at week 24) switch to DTG monotherapy and followed throughout 48 weeks from the date of switch, was prematurely stopped because of virological failure in 10% of patients (74, 75). While DTG monotherapy was non-inferior to cART at week 24, most of virological failures occurred after week 24 and led to INSTI-associated resistance mutations in three patients.

In addition, the Data Safety Monitoring Board of the DOLAM study, an open-label randomized controlled trial (EudrCT number 2015-000274-35) which enrolled HIV-1-infected adults on stable triple antiretroviral therapy to receive cART, DTG + 3TC or DTG monotherapy, decided to interrupt the DTG monotherapy arm because of the occurrence of two virological failures in this arm (76).

Finally, the REDOMO study showed that the percentage of virological failures in three large clinical cohorts of individualy switching to a DTG-monotherapy maintenance strategy was 9%. The selection of genotypic resistance mutations in individuals failing to DTG-monotherapy was rapid and high. The mutations selected on virological failures to DTG-monotherapy involves different pathways of resistance to INSTI (92Q, 118R, 148X and 155H) (77).

Dolutegravir efficacy data in dual therapy (either in ART-naive or experienced patients)

The PADDLE study, a single-arm pilot trial for patients with HIV-RNA<100,000/ml copies at screening, demonstrated an overall efficacy of DTG-3TC of 90% as *first-line treatment*. One patient with virologic failure had an entry HIV RNA >100,000 copies and was suppressed initially, but developed low-level viremia from week 36-48; no resistance was detected (78). Bitherapy of DTG and rilpivirine was tested among 38 experienced patients who had benefited from several ART regimens. Participants presented with significant major resistant mutations to NRTIs, NNRTIs and PIs but none of them had resistance to INSTI or rilpivirine (79). All patients were virologically suppressed at week 48, showing that heavily pre-treated patients can safely be switched to simplified ART. Moreover, in vitro studies have shown that the presence of NRTIs resistance mutations M184I/V and K65R prevent the emergence of HIV-1 resistance against DTG, but not RAL or EVG (80).

The LAMIDOL study, which enrolled 104 HIV experienced patients without previous virological failure to switch to a dual maintenance therapy of DTG + 3TC (lamivudine) showed that 97% (101/104) of participants remained virologically suppressed at week 40(81). 7.7% (8/104) of participants experienced serious adverse events; only 1 patient discontinued study drugs due to suicide ideation.

A summary of the studies on DTG monotherapy and dual therapy that have been presented or published so far is given in the Supplementary Files 1 and 2.

In the SIMPL'HIV trial, we chose to evaluate the efficacy and safety of DTG + FTC dual maintenance

therapy in HIV virologically suppressed experienced patients. The choice of the combination of DTG with FTC is based on (1) the superiority of emtricitabine 200 mg once daily when compared to 3TC (lamivudine) (70), (2) the fact that no study – to our knowledge - is currently evaluating this combination, and (3) the soon generically available dual maintenance therapy combining DTG and FTC.

3.5 Dose Rationale

This trial will be using the dose of DTG 50mg in combination with FTC 200 mg oral per day, which corresponds to the doses recommended by the manufacturers.

3.6 Explanation for choice of comparator

HIV guidelines provide clear recommendations of what a first-line and maintenance regimen should be according to the latest publications (82, 83). Based on recommendations of the European AIDS Clinical Society (EACS) guidelines dated of October 2015 (84), recently updated in October 2017(63) we will accept as standard of care any of the following ART regimens that achieved viral suppression for at least 24 weeks, i.e.:

- 2 NRTIs + either 1 NNRTI, 1 boosted PI or 1 INSTI;
- NRTI-sparing triple ARV regimen (e.g. 1 NRTI + 1 NNRTI + 1 InSTI);
- Dual therapy with protease inhibitor (such as boosted PI + NRTI or NNRTI or InSTI).

We will exclude the following regimens:

- Two-drug combinations with: 1 NRTI + 1 NNRTI, 1 NRTI + 1 PI without RTV, 1 NRTI + RAL, 2 NRTIs, MVC + RAL (not recommended by EACS guidelines);
- Triple NRTIs combination (not recommended by EACS guidelines);
- Dual therapy with DTG other than PI-based dual regimens, such as DTG + NRTI or DTG + NNRTI (simplified regimen already);
- PI monotherapy (simplified regimen already).

3.7 Risks / Benefits

The main risk in this trial is virological failure on DTG + FTC dual therapy. In the ART Cohort Collaboration of 18 cohorts from Europe and North America including about 17900 patients, low level viremia measured between 50 and 199 copies/ml was weakly associated with virological failure (aHR 1.38, 95%CI 0.96-2.00) (85). We will measure HIV-1 RNA level in the plasma on a regular basis to monitor for low level viremia. Any patient with HIV-1 RNA ≥100 copies/ml will undergo an additional HIV-1 RNA measurement 14 days later (± 5 days), as well as additional adherence session and review of concomitant medications and dolutegravir and emtricitabine plasma concentration measurements.

We will define virological failure as plasma HIV-1 RNA ≥100 copies/ml on two consecutive measurements. Any patient reaching virological failure will be assessed for genotypic resistance and pharmacogenetic analyses. This latest analysis will be only done in patients who are included in the SHCS and have also signed a consent form for genetic studies. In case of virological failure, the patient will undergo an additional medical visit with review of adherence, concomitant medications, discussion of results of DTG + FTC plasma concentration. Patients randomised in the DTG + FTC dual therapy arm will be switched to a standard cART regimen while waiting for results of genotypic resistance. Patients randomised in the continuing cART arm will await results of the genotypic results. In both cases, treatment will be adjusted according to genotypic resistance results. All failing patients will complete study procedures until week 48. Figure 3 presents the algorithm for the management of virological failure.

Any patient with HIV-RNA between 20 and 99 copies at any time of scheduled visits will have an additional HIV-RNA measurement after 6 weeks (+/-5 days).

Figure 3: Algorithm for the management of virological failure

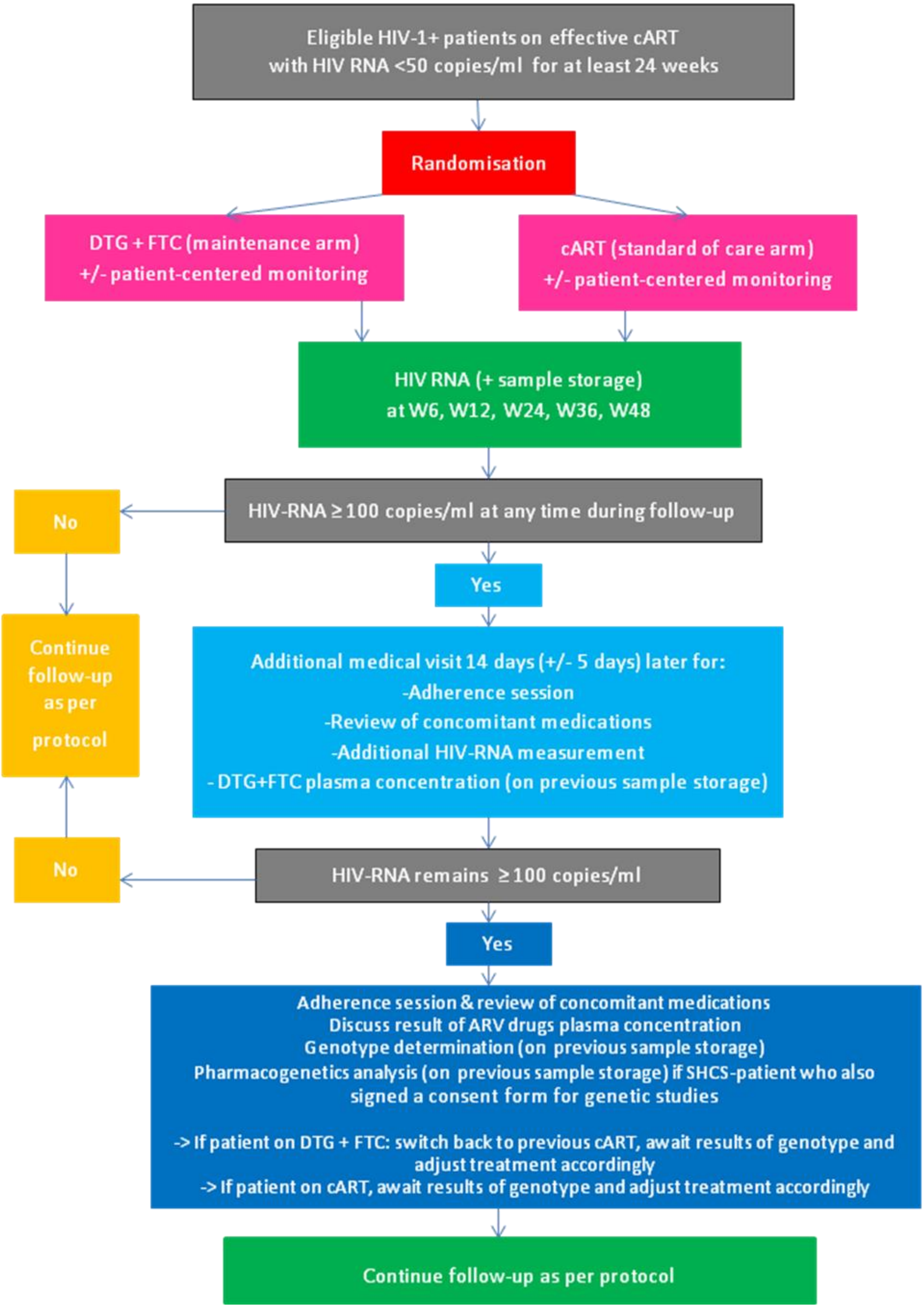

Other risks associated with the trial consist of the occurrence of DTG-related side effects as observed in phase III trials and observational studies, and described in section 3.2. Side effects were relatively uncommon, with the exception of CNS adverse events in observational studies. These CNS side effects led to few discontinuation of the drug. However, patients randomised in the DTG + FTC dual therapy arm and “new” to DTG will be evaluated for CNS symptoms using a standardised questionnaire at week 2 (by phone call by the study nurse/physician) and at week 6.
Emtricitabine is a well tolerated drug with little toxicity: in trials of patients with HIV or HBV infection, adverse events occurring with at least 10% frequency include gastro-intestinal symptoms with 14% to 18% of nausea and 9% to 24% of diarrhea, asthenia (10%-16%) and headache (12%18%) (69). Very few of the adverse events were categorized as serious.

Potential benefits to participants may include a simplification of antiretroviral therapy and a decrease in potential side effects related to other antiretroviral drugs.

3.8 Justification of choice of study population

This maintenance trial will enroll HIV-positive individuals on first-line or maintenance antiretroviral therapy who are virologically suppressed and included in the SHCS or getting care from a medical doctor of the SHCS network. The SHCS is highly representative of the HIV epidemic in Switzerland with an estimated coverage of 75% of HIV-infected individuals, 69% of all patients with AIDS in Switzerland, and 72% of all ART-treated individuals. Patients will be recruited during routine consultations in the outpatient clinics of the participating sites of the SHCS network. The study does not foresee inclusion of vulnerable participants or enrolment in emergency situations.

4. STUDY OBJECTIVES

4.1 Overall Objective

The purpose of this study is to evaluate whether maintenance antiretroviral therapy could be simplified to DTG + FTC dual therapy and/or patient-centered monitoring once virological suppression is achieved. Using a factorial design, the study aims to assess the efficacy of DTG + FTC dual therapy to maintain virological suppression through 48 weeks of follow-up as well as the costs of a patient-centered ART laboratory monitoring. The study will also describe the safety and acceptability of the different study arms.

4.2 Primary Objective

The study seeks primarily to assess:

- The efficacy of DTG + FTC dual therapy to maintain virological suppression throughout 48 weeks in virologically suppressed HIV-infected individuals who are switched from standard therapy to DTG + FTC dual therapy.
- The costs from the perspective of the health care provider of a patient-centered ART monitoring strategy which offers alternative options for the conduction of study visits, venipunctures, drugs delivery, and which omits CD4 cell count, lipid and glucose measurements, and safety blood tests (CK, renal, liver and full blood count) during follow-up.

4.3 Secondary Objectives

Secondary objectives are to assess:

- Loss of future drug options in patients with virological failure, predictors of virological failure, time to loss of virological response (TLOVR), change in CD4 cell count, change in HIV-DNA blood reservoir, adherence, DTG and FTC plasma concentration, safety, treatment satisfaction, acceptability, health-related quality of life, costs of treatment arms;
- Acceptability, safety, cost-effectiveness, and health-related quality of life of monitoring arms.

4.4 Safety Objectives

The study aims to assess the safety of DTG + FTC dual therapy and simplified ART monitoring throughout 48 weeks by evaluating the following parameters:

- Loss of future drug options in case of virological failure defined as new intermediate or high-level resistance to one or more drugs to which the patient’s virus was considered sensitive at trial entry;
- Change in CD4 cell count, total cholesterol, low density lipoprotein-cholesterol, Framingham-calculated cardiovascular risk, and glomerular function rate;
- Occurence of adverse events**;**
- Occurence of serious adverse events**;**
- Occurrence of CNS adverse events in patients new to DTG.

5. STUDY OUTCOMES

5.1 Primary Outcomes

The *first primary outcome* for the comparison between DTG + FTC dual therapy and standard cART is the proportion of patients maintaining HIV suppression defined as HIV-RNA <100 copies/ml throughout 48 weeks (+/- 21 days). A single blip defined as HIV-RNA <200 copies/ml during the study period is allowed as long as this value is followed by HIV-RNA <50 copies/ml.

The *second primary outcome* for the comparison between patient-centered and standard monitoring is the direct costs of the two study arms from the health care system perspective at week 48.

5.2 Secondary Outcomes

1. Proportion of patients maintaining HIV suppression defined as HIV-RNA <50 copies throughout 48 weeks (+/- 21 days). A single blip defined as HIV-RNA <200 copies/ml during the study period is allowed as long as this value is followed by HIV-RNA <50 copies/ml;
2. Proportion of patients with HIV-RNA < 50 cp/ml at week 48 by FDA snapshot analysis; Proportion of patients experiencing loss of future drug options defined as new intermediate or high-level resistance to one or more drugs to which the patient's virus was considered sensitive at trial entry;
3. Time to loss of virological response (TLOVR) up to week 48 defined as the first of the two-confirmed HIV-RNA >100 copies/ml (at least two weeks apart);
4. Change in CD4 cell count from baseline to week 48 +/- 21 days;
5. Change in HIV-DNA level from baseline to week 48 +/- 21 days;
6. Change in lipidic profile (total cholesterol, high density lipoprotein-cholesterol, triglycerides, and low density lipoprotein-cholesterol) from baseline to week 48 +/- 21 days;
7. Change in glucose profile from baseline to week 48;
8. Change in Framingham-calculated cardiovascular risk from baseline to week 48;
9. Change in glomerular function rate from baseline to week 48;
10. Proportion of patients with an adverse event throughout week 48;
11. Proportion of patients with a serious adverse event (SAE) throughout week 48;
12. Proportion of patients with a CNS adverse event throughout week 48;
13. Proportion of patients new to DTG with CNS symptoms at 2 and 6 weeks after starting DTG;
14. Change in patients' health-related quality of life from baseline to weeks 12 and 48;
15. Assessment of patient's monitoring satisfaction from baseline to weeks 24 and 48 for patients in the patient-centered monitoring arm;
16. Assessment of the global satisfaction of the monitoring at week 48 for all patients (regarless of the randomisation arm);
17. Proportion of patients in the patient-centered monitoring arm expressing willingness to change monitoring options throughout 48 weeks;
18. Assessment of patient's treatment satisfaction at week 48;
19. Assessment of ARV treatment in the post study;
20. Study satisfaction at week 48;
21. Cost-effectiveness of study arms at 48 weeks;
22. Changes in patient weight from baseline to week 48.

5.3 Other Outcomes of Interest

1. Assessment of patient adherence to treatment throughout 48 weeks of follow-up;
2. Number of extra-visits performed outside trial scheduled throughout 48 weeks.

5.4 Safety Outcomes

1. Change in CD4 cell count from baseline to week 48;
2. Change in lipidic profile (total cholesterol, high density lipoprotein-cholesterol, triglycerides, low and high density lipoprotein-cholesterol) from baseline to week 48;
3. Change in glucose profile from baseline to week 48;
4. Change in Framingham-calculated cardiovascular risk from baseline to week 48;

5. Change in glomerular function rate from baseline to week 48;
6. Proportion of patients with an adverse event at week 48;
7. Proportion of patients with a serious adverse event at week 48;
8. Proportion of patients with a CNS adverse event throughout 48 weeks
9. Proportion of patients new to DTG with CNS symptom at 2 and 6 weeks after starting DTG;
10. Changes in patient weight from baseline to week 48.

6. STUDY DESIGN

6.1 General study design and justification of design

This is a pragmatic multicentre, 2 x 2 factorial randomized controlled trial with 1:1:1:1 randomization to switching to DTG + FTC dual therapy or continuation of cART and to patient-centered monitoring or continuation of standard monitoring (Figure 4).

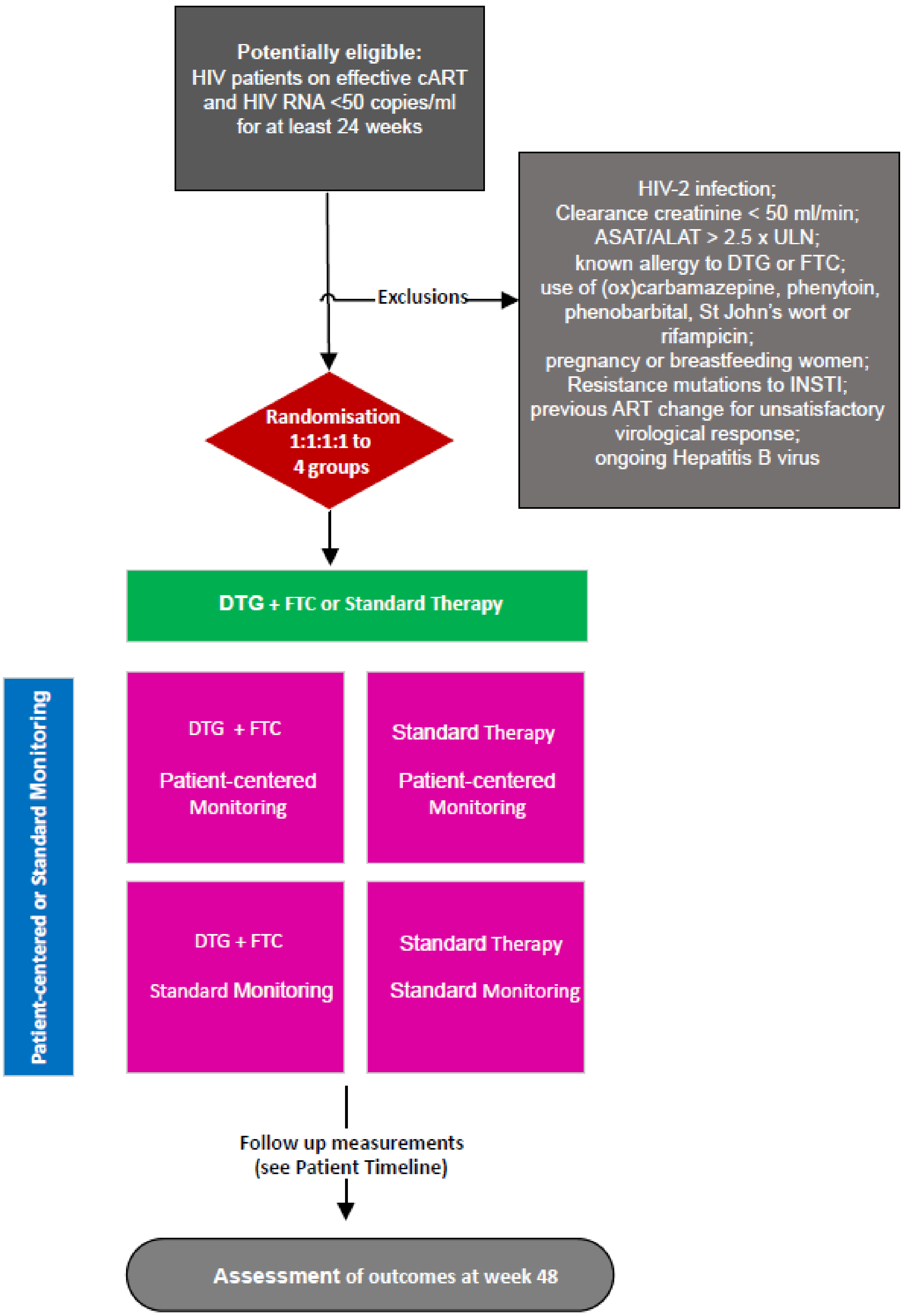

Figure 4: Schematic presentation of study design DTG + FTC dual therapy

HIV-infected patients virologically suppressed for at least 24 weeks on standard cART will be randomly allocated:

- Standard-of-care arm: patients randomized in this arm will continue their current cART regimen, according to section 7.1.
- Dual therapy arm: patients randomized in this arm will be switched to DTG + FTC dual therapy. DTG 50 mg and FTC 200 mg will be administered once daily as dual therapy for a duration of 48 weeks.

For all patients, HIV-RNA measurements will be performed at screening, baseline, week 6, week 12, and every 12 weeks afterwards. Any patient with HIV-RNA between 20 and 99 copies/ml at any time of scheduled visits will have an additional HIV-RNA measurement after 6 weeks (± 5 days). Any patient with HIV-RNA > 100 copies/ml at any time of scheduled visits will have an additional HIV-RNA measurement after 2 weeks (± 5 days).

Patient-centered monitoring

Randomization will also allocate patients into the following two groups:

- Standard monitoring arm: patients randomized in this arm will continue a standard 12-weekly routine immunological and safety blood monitoring, including CD4 cell count, lipids and glucose, renal and hepatic function tests, creatine kinase at their affiliated SHCS sites.

- Patient-centered monitoring arm: patients randomized in this arm will have immunological and safety blood analyses (CD4 cell count, lipids and glucose, renal and hepatic function tests, creatine kinase) performed at screening and at week 48. Additional safety blood tests will be performed in case of signs and symptons as indicated by the physician. In addition, patients will have to choose at least one of three alternative options for the conduction of study visits, venipuncture and drugs delivery at weeks 6, 12 and 36, see section 8.1.1. Patients will also have the opportunity to change or give their opinion on the alternative option(s) chosen if they find it non convenient during the study. Changes will be possible at week 24; satisfaction will be assessed at week 12, 24, 36 and 48.

Importantly, all interventions or blood measurements potentially related to adherence (affecting the primary endpoint) will be identical between the study arms (see section 8.4).

6.2 Methods of minimising bias

6.2.1 Randomisation

Allocation sequence

Patients will be randomised 1:1:1:1 using randomly-permuted blocks of varying sizes to one of the four study arms (one computer-based randomization list stratified per study center).

Concealment mechanism

Each of the 7 centres will receive the attribution number using a central computer-based randomization system.

Implementation

One allocation sequence stratified per study center will be generated by an independent team who will not be involved in the implementation of the study on sites using a computer-generated sequentially numbered randomization list. Lists will be transferred to the electronic case report form based on a standardized process. The process ensures that lists are stored with restricted access so that only system administrators have access. Once eligibility criteria are confirmed and when the patient has provided oral written consent to participate in one of the 7 centers enrolled in the trial, the study nurse on site (or the study investigator) will register the patient in the electronic data capture system after which the allocation will be revealed.

6.2.2 Blinding procedures

This will be an open-label trial.

6.2.3 Other methods of minimising bias

The use of validated questionnaires on adherence, health related quality of life and treatment satisfaction will help minimising information biais. The following validated questionnaires will be used:

- Adherence will be measured using the questionaire reported by Glass, Sterne et al. 2015 (86) and used during routine HIV consultations within the SHCS.
- Health related quality of life will be measured using the PROQOL-HIV questionnaire (87, 88) together with a visual analog scale.
- Central nervous system symptoms will be assessed using the depression module PHQ-9 recommended by the WHO (89, 90).

6.3 Unblinding Procedures (Code break)

Not applicable. This is an open-label trial.

7. STUDY POPULATION

HIV-1 positive individuals included in the SHCS or getting care from a medical doctor of the SHCS network will be enrolled in this maintenance trial. All SHCS centres will participate in the study. Patients relocating within Switzerland can be followed in any of the participating SHCS centres.

7.1 Eligibility criteria

Participants fulfilling all of the following <u>inclusion criteria</u> are eligible for the study:

1. Informed consent as documented by signature
2. Documented HIV-1 infection;
3. Enrolled in the SHCS or receiving care from a medical doctor of the SHCS network;
4. ≥ 18 years of age;
5. HIV-RNA <50 copies/mL at screening and for at least 24 weeks before screening on effective suppressive cART, one blip with less than 200 copies/mL allowed during this period if followed by at least 2 results < 50 copies/mL;
6. On standard cART at the time of inclusion, i.e.:
   - 2 NRTIs + either 1 NNRTI, 1 boosted PI or 1 INSTI;
   - NRTI-sparing triple ARV regimen (e.g. 1 NRTI + 1 NNRTI + 1 InSTI);
   - Dual therapy with protease inhibitor.

Every effort will be made to include women (91) in the same proportion as in the prospective SHCS (28% of HIV-infected individuals included into the SHCS are female; SHCS website demographic data, accessed 20 December 2017).

The presence of any of the following <u>exclusion criteria</u> will lead to exclusion of the participant:

1. HIV-2 infection;
2. Previous ART change for unsatisfactory virological response, i.e. slow initial virological suppression, incomplete suppression or rebound; change for convenience or toxic effect prevention or management allowed.
   Note: patients with documented genotype(s) presenting only a M184V mutation remain eligible;
3. Creatinine clearance <50 ml/min;
4. ASAT or ALAT > 2.5x upper limit of the norm;
5. Known hypersensitivity, intolerance or allergy to DTG or FTC;
6. Known or suspected non-adherence (defined as <80% adherence, i.e. missed dose > 1x/week), to current treatment in the last 24 weeks;
7. Concomitant use of drugs that decrease dolutegravir blood concentrations including carbamazepine, oxcarbamazepine, phenytoin, phenobarbital, St John's wort and rifampicin;
8. Women who are pregnant or breast-feeding;
9. a. Presence of any InSTI-resistance. (92).
   b. Non availability of previous routine resistance test, at least for reverse transcriptase and protease genes.
   Note: Subjects remain eligible in the absence of any previous resistance test only if they are on their first-line antiretroviral regimen;
10. Evidence of acute or chronic hepatitis B virus infection based on results of serology testing as follows:
    - subjects positive for HBsAg are excluded (corresponding to acute or chronic infection);
    - only positive anti-HBc subjects will be excluded if positive HBV-DNA;
    - susceptible [*] (negative HBsAg, anti-HBc and anti-HBs), immune due to hepatitis B vaccination (negative HBsAg and anti-HBc and positive anti-HBs) and immune due to natural infection (negative HBsAg and positive anti-HBc and anti-HBs) subjects are not excluded.

---

[*] HBV-susceptible subjects will be offered with immunization as standard-of-care practice.

Patients enrolled in other interventional studies are not eligible. After the other interventional study termination, they will have to wait at least 3 months before the Simpl'HIV screening.

7.2 Recruitment and screening

7.2.1 Recruitment strategies

Patients will be recruited using the SHCS database. According to the database, it is estimated that >2500 individuals followed at the participating centres in 2014 (out of 8900 participants) are potentially eligible. However, the genotype is not available to screen patients solely on the basis of the SHCS database and the potential number of candidates will be therefore downgraded to 1200. If 15% of the patients agree to participate, the recruitment will be completed within a year.

Possible candidates will be contacted by their attending physician or delegates (e.g.study nurse, study coordinator) and will undergo screening procedures according to the protocol after giving written consent. No advertising is intended at this stage other than information about the protocol (flyer in appendix 2) in the study centers and local HIV/AIDS associations. Participants will not receive financial compensation for participation. Recruitment will continue until the target sample size is reached. In the event of slow or insufficient recruitment, study sites visits will be conducted and human resources allocated. In addition, an international collaboration could be sought in case a rapid recruitment is needed.

7.2.2 Feasibility of recruitment

The prospective research cohort (SHCS) has been already used successfully to plan randomized controlled trials and other clinical trials in the field of HIV in Switzerland, including SNF grants (SNF grant 32003B_135745). As the SHCS is highly representative of the HIV care and treatment network in Switzerland, with over 75% of all HIV-infected individuals included this cohort in Switzerland (93), we consider that we have an excellent tool to estimate patient recruitment.

All participating SHCS sites agree to recruit patients according to the following table:

Site	Number of patients
Geneva	34
Lausanne	24
Lugano	12
Bern	28
Zürich	50
St Gallen	12
Basel	24

7.3 Assignment to study groups

7.3.1 Allocation sequence

Patients will be randomised 1:1:1:1 using randomly-permuted blocks of varying sizes to one of the four study arms (one computer-based randomization list stratified per study center).

7.3.2 Concealment mechanism

Each of the 7 centres will receive the attribution number using a central computer-based randomization system.

7.3.3 Implementation

One allocation sequence stratified per study center will be generated by an independent team who will not be involved in the implementation of the study on sites using a computer-generated sequentially numbered randomization list. Lists will be transferred to the electronic case report form based on a standardized process. The process ensures that lists are stored with restricted access so that only

system administrators have access. Once eligibility criteria are confirmed and when the patient has provided oral written consent to participate in one of the 7 centers enrolled in the trial, the study nurse on site (or the study investigator) will register the patient in the electronic data capture system after which the allocation will be revealed.

7.4 Criteria for withdrawal / discontinuation of participants

Participants will be withdrawn from the trial if:

1. The subject withdraws consent; in this case the patient will continue the antiretroviral treatment with a standard-of-care treatment (cART) and will continue regular follow-up with his/her treating physician;
2. Clinical reasons believed to be life-threatening by the physician, even if not addressed in the toxicity management protocol;
3. Subject is judged by the investigator to be at significant risk of failing to comply with the provisions of the protocol so as to cause harm to self or seriously interfere with the validity of the study result.
4. Study routine procedure must be stopped due to safety concerns.

Withdrawn participants will not be replaced.

Participants in the DTG + FTC dual therapy maintenance arm will discontinue study drugs if (non exhaustive list):

- diagnosed with virological failure defined as plasma HIV-RNA ≥100 copies/ml on two consecutive occasions;
- become pregnant
- start breastfeeding;
- requiring to use prohibited concomitant medications such as carbamazepine, oxcarbamazepine, phenytoin, phenobarbital, rifampicin or St John's Wort;
- presenting serious adverse events that necessite to stop dolutegravir and/or emtricitabine;
- reasons related to conceiving and DTG potentially safety issue.

They will still be included in the study, continue their antiretroviral treatment with a standard cART. They can be switched to their previous ARV regimen or to a different one according to the genotypic resistance testing, drug-drug interactions profile and/or clinical evolution, and will complete study procedures until week 48. If the new regimen still fulfills the eligibility criteria the subject can continue the study, otherwise the subject will be withdrawn from the trial. In case of a pregnancy the viral load measurement will be performed once per month accordingly to the EACS guidelines.

8. STUDY INTERVENTION

8.1 Identity of Investigational Products (treatment)

8.1.1 Experimental Intervention (treatment)

Dolutegravir 50 mg (Tivicay®) is manufactured by ViiV Health Care. It is a round pale yellow tablet to be taken orally once daily with or without food. DTG 50 mg will be administered once daily in combination with FTC for a duration of 48 weeks.

Emtricitabine 200 mg (Emtriva®) is manufactured by Gilead Sciences. It is blue and white capsule to be taken orally once daily with or without food. FTC 200mg will be administered once daily in combination with DTG for a duration of 48 weeks.

Patient-centered monitoring will include reduced immunological and safety blood analyses (CD4 cell count, lipids and glucose, creatine kinase, renal and hepatic function tests) performed at screening and at week 48. In addition, patients will be offered alternative options for the conduction of study visits, venipuncture and drugs delivery at weeks 6, 12 and 36. Participants will be asked to select at least one of the three following strategies:

- Venipuncture for blood analyses: decentralised venipuncture, e.g. at peripheral laboratory near patient's home or place of work, or with general practitioner, versus at their affiliated SHCS site;
- Drug delivery: delivery of ARV drugs/prescription by mail or at a chosen pharmacy versus at their affiliated SHCS site;
- Assessment and clinical interview: phone call with study nurse/physician versus face-to-face at their affiliated SHCS site.

These options will be organized at study site discretion. Table 1 summarises the differences between the monitoring arms and Table 2 lists the different options chosen by each study site.

Table 1: Summary of the differences between standard and patient-centered monitoring arms

STANDARD MONITORING*		PATIENT-CENTERED MONITORING*
All visits at SHCS center	**WHERE**	Weeks 24 and 48 visits at SHCS center<br><br>Weeks 6, 12, 36 according to patient choice (see HOW below)
Week 6:<br>- HIV-RNA measurement only<br>Weeks 12, 24, 36 and 48 visits:<br>- Complete blood analysis with HIV-RNA, CD4 count, full blood count, safety serum chemistry	**WHAT**	Week 6, 12, 24, 36:<br>- HIV-RNA measurement only<br>Week 48:<br>- Complete blood analysis with HIV-RNA, CD4 count, full blood count, safety serum chemistry
- Face to face study visit<br>- Venipuncture with study nurse<br>- Drug or prescription delivery according to site usual practice	**HOW**	At least one of the following options:<br>- Visits: Phone call versus at SHCS center<br>- Venipuncture: peripheral versus at SHCS center<br>- Delivery of drugs: by mail or at chosen pharmacy versus at SHCS center

* No differences for screening and baseline visit

Table 2: Patient-centered care arm options available at each study site

	Visit	Laboratory	Study drug
**Geneva**	Phone call	- Venipuncture at Dianalabs<br>- HIV RNA, CD4 and storage at HUG	DHL (address chosen by the patient)
**Lausanne**	Phone call	- Venipuncture at La Source<br>- Storage at CHUV	Decentralized pharmacy (chosen by the patient)<br>Labels will be attached to the IMP bottles by the patient
**Neuchâtel**	Phone call	- Venipuncture at GP office<br>- Storage at CHUV	Decentralized pharmacy (chosen by the patient)<br>Labels will be attached to the IMP bottles by the patient
**Lugano**	Phone call	Not available	La Poste (address chosen by the patient)
**Zurich**	Phone call	- Venipuncture at GP office<br>- All analyses at USZ laboratory<br><br>Not feasible for patients on DTG/metformin<br>Not feasible for patients new to DTG at week 6	Decentralized pharmacy (chosen by the patient)<br>Labels will be attached to IMP bottles by the patient
**Bern**	Phone call	- Venipuncture at GP office<br>- Analyses at Inselspital	La Poste (address chosen by the patient)
**St. Gallen**	Phone call	- Venipuncture at GP office<br>- Analyses at Kantonsspital and at peripheral lab	La Poste (address chosen by the patient)
**Basel**	Phone call	- Venipuncture at GP office<br>- Analyses at Universitätsspital	La Poste (address chosen by the patient)

Patients will also have the opportunity to change or give their opinion on the alternative option chosen if they find it non convenient during the study. Changes will be possible at week 24; satisfaction will be assessed at week 12, 36 and 48.

8.1.2 Control Intervention (standard/routine/comparator treatment)

Patients in the control intervention will be on fully suppressive antiretroviral regimen.

The control intervention may include several options:

- 2 NRTIs + either 1 NNRTI, 1 boosted PI or 1 INSTI;
- NRTI-sparing triple ARV regimen (e.g. 1 NRTI + 1 NNRTI + 1 InSTI);
- Dual therapy with protease inhibitor (such as PI + NRTI or NNRTI or InSTI).

Within-class antiretroviral switch (such as switch from TDF to TAF) are accepted during the study period only for patients randomized in the continuing cART arm. Other antiretroviral switch are discouraged and sponsor agreement is needed before the switch.

The following regimens will be excluded:

- Two-drug combinations with: 1 NRTI + 1 NNRTI, 1 NRTI + 1 PI without RTV, 1 NRTI + RAL, 2 NRTIs, MVC + RAL (not recommended by European guidelines);
- Triple NRTIs combination (not recommended by European guidelines);
- Dual therapy with DTG other than PI-based dual regimen, such as DTG + NRTI, DTG + NNRTI (simplified regimen already);
- PI monotherapy (simplified regimen already).

Standard monitoring includes a standard 12-weekly routine immunological and safety blood monitoring, including CD4 cell count, lipids and glucose, creatine kinase, renal and hepatic function tests, at affiliated SHCS sites.

8.1.3 Packaging, Labelling and Supply (re-supply)

The commercial product of Dolutegravir (Tivicay®), Emtricitabine (Emtriva®) and other antiretroviral drugs will be administered in this study (without deviation, i.e. only commercial product will be administered). The ARV drugs will be distributed to the participants according to usual local practice at the study centers. Each study centre distributing ARV will be resupplied using the hospital's routine order system. The study drugs (Tivicay® and Emtriva® and cART drugs) will be labelled with the following information in the local language: Short title of the study, Protocol CCER number, patient ID number, name of sponsor-investigator and/or principal investigator. Additional information such as date of IMP/prescription distribution can be added at site's discretion.

8.1.4 Storage Conditions

The investigational product will be stored securely at the study centers distributing ARV with limited access and under the recommended storage conditions.

ARV drugs must be stored at maximum 30°C. Study centre distributing ARV will be equiped with a temperature monitoring system and document storage temparatures in the investigator site file.

Temperature deviations of >30°C must be reported to the sponsor-investigator.

8.2 Administration of experimental and control interventions

8.2.1 Experimental Intervention

Dolutegravir 50 mg and Emtricitabine 200 mg will be administered per os once daily as dual therapy for a duration of 48 weeks. Participants will be requested to take the IMPs at approximately the same time each day (+/- 1 hour) with or without food. Dolutegravir and emtricitabine will be dispensed from study site or local pharmacy according to site practice at each scheduled visit. Study drug dispensing and return information will be recorded on the CRFs and drug accountability logs.

At the end of the study, all patients will be offered the possibility to continue their treatment with either a standard ART regimen or DTG + FTC dual therapy. This will be decided at treating physician discretion after discussion with the patient and depending on the efficacy of the strategy during the

study period. Subsequent follow-up will be made at the discretion of the treating physicians.

Participants in the patient-centered monitoring arm will be asked to select at least one of the three different options:

- Venipuncture for blood analyses: decentralised venipuncture, i.e. at peripheral laboratory nearer to their home or place of work, or with general practitioner, versus at their affiliated SHCS site;
- Drug delivery: delivery of ARV drugs/prescription by mail or at chosen pharmacy versus at their affiliated SHCS site;
- Assessment and clinical interview: phone call with study nurse/physician versus fa-to-face at their affiliated SHCS site.

At the end of the study, patients will continue regular monitoring as per SHCS requirements.

8.2.2 Control Intervention

Participants randomised in the continuing cART arm will continue to take their standard fully suppressive antiretroviral therapy as previously, once or twice daily according to their antiretroviral regimen, at approximatively the same time (+/- 1 hour) with or without food. All recommended American and European regimens are administered per os.

Patients randomized in the standard monitoring arm will continue a standard 12-weekly routine immunological and safety blood monitoring, including CD4 cell count, creatine kinase, lipids and glucose, renal and hepatic function tests, at their affiliated SHCS sites.

8.3 Dose modifications

The dose of emtricitabine must be adapted according to kidney function. The dose of 200 mg once a day is the dose for a creatinine clearance of ≥ 50ml/min. As participants will be excluded if creatinine clearance is < 50 ml/min, there is no need for dose modification at enrolment. However, if creatinine clearance decreases during the study period, dose modification of emtricitabine will be made according to European guidelines, i.e.

- 200 mg every 48 hours if creatinine clearance between 30 and 49 ml/min (calculation preferably according to the CKD-EPI);
- 200 mg every 72 hours if creatinine clearance between 10 and 29 ml/min (calculation preferably according to the CKD-EPI);

  200 mg every 96 hours creatinine clearance <10 ml/min or haemodialysis (calculation preferably according to the CKD-EPI).

8.4 Compliance with study intervention

Since non-adherence is one reason for simplified maintenance failure, special care will be given to optimize adherence. Patients known or suspected to be non-adherent will not be eligible for the study. All centres will apply standard specific interventions to promote patient adherence.

- Adherence will be documented at each visit, being face-to-face or through phone call, as participants will answer specific questions, identical to the questions used at the routine visits of the SHCS (86);
- Patients will be asked to fill in a diary with daily documentation of the date and time of drug intake, concomitant health conditions and medications at least in the case of any deviation from usual practice;
- Pill count will be performed at each visit, being face-to-face or through phone call.

Patient’s adherence will be calculated as a percentage. Patients should have taken at least 80% of treatment pills during the study period to be considered in the per protocol analysis.

Patients with HIV-RNA ≥100 copies/ml at any time during the study will also benefit from DTG + FTC plasma concentration measurement.

8.4.1 Retention

Participant retention will be promoted using different strategies:

- Participants will be reminded of the critical importance of drug intake during visits, either face-to-face or through phone call;
- SMS reminders will be sent to patients 1 day prior to each scheduled study visit where feasible;
- Efforts will be made to adjust the timing of the study visits to the schedule of the patient whilst respecting the protocol given visit windows.

8.5 Data Collection and Follow-up for withdrawn participants

Participants who withdraw from the study will be asked to come in for a close-out visit (named study discontinuation visit) where all assessments planned at week 48 will be done.

8.6 Trial specific preventive measures

Concomitant medication(s) will be reviewed at baseline, at each visit thereafter and in case HIV-RNA is measured ≥ 100 copies/ml. Patients receiving anti-epileptic drugs such as carbamazepine, oxcarbamazepine, phenytoin, phenobarbital, St John’s wort, or receiving the antibiotics rifampicin will not be eligible for the study. The concomitant administration of these drugs with dolutegravir decreases the plasma concentration of dolutegravir, and thus increasing the risk of virological failure. Patients on anti-tuberculosis treatment may be eligible at completion of anti-TB drugs. The use of these drugs is part of the exclusion criteria to the study and will be recorded in the screening CRF.

Pregnant women or breastfeeding mothers will not be eligible for the study as triple antiretroviral therapy is standard of care for these conditions. Pregnancy and breastfeeding are part of the exclusion criteria and will be recorded in the CRF.

Women of child-bearing potential will be screened for pregnancy at the screening visit of the study. Those who are found to be pregnant will be excluded from study participation (i.e. screening failure) and referred to an appropriate medical service.

Women of child-bearing potential included into the trial will further undergo urine pregnancy test once every 6 months (i.e. at week 24 and 48 visits). Results of screening and follow-up urine pregnancy tests will be recorded in the study CRF.

Women who become pregnant or start breasfeeding after randomisation will be switched to standard triple cART not containing dolutegravir and will continue the study. They will continue to be monitored for the development of adverse events (AE) or serious adverse events (SAE), and for pregnancy outcome.

After May 23, 2018, women of child-bearing potential and on dolutegrvir are quickly identified by Sponsor-investigator and Study Sites and these women are all informed about the safety signal concerning DTG use in early pregnancy and risk of neural tube defect in infants born to women using DTG in early pregnancy. As communicated on May 25, 2018 to the leading Ethics Committee and to the DSMB (see appendix n° 7 and n° 8 respectively), these women are all asked to sign an information sheet explaining this risk. In case of willingness of pregnancy, they are switched to DTG-free regimen, at study investigator’s discretion. In case of non-willingness of a pregnancy, the use of an effective contraceptive method (oral, injectable, implantable contraceptive and intrauterine contraceptive device are considered effective contraceptive methods as well as condom in stable couple and sexual abstinence) is proposed, if not already used by the women. Women of child-bearing potential, on dolutegravir, without willingness of a pregnancy, and refusing to use an effective contraceptive method are proposed to change their ART to a DTG-free regimen. If any of the above procedure is not respected, the sponsor will decide to withdraw the concerned patient from the study.

8.7 Concomitant Interventions (treatments)

Co-administration of the anti-diabetic drug metformine with dolutegravir increases the plasma concentration of metformine; a dose adjustment of metformine is needed with limiting total daily dose to 1000 mg. As metformin is eliminated renally, patients with moderate renal impairment may be at

increased risk for lactic acidosis due to increased metformin concentrations. Thefore, blood glucose and kidney function will be screened in diabetic patients under metformine at the start and during co-administration with dolutegravir + emtricitabine dual therapy, i.e. week 6, 12, 24 and 36. If creatinine clearance falls below 45ml/min, metformine will be stopped as per Swiss endocrinology and diabetology guidelines (94) and a switch to another oral antidiabetic drug will be made and documented in the study CRF.

Participants receiving magnesium-, aluminium- or calcium-containing anti-acids or vitamin supplements with calcium, iron, or magnesium will take dolutegravir 2 hours before or 6 hours after co-administered drugs.

Concurrent participation in non-interventional clinical trials is allowed as long as there is no impact on the objectives of this trial.

8.8 Study Drug Accountability

Study centre distributing ARV will order its own study drugs using the hospital's supply route. The ARV drug bottles will be labelled with study specific labels. Participants will be asked to return ARV drug bottles (full, partially empty and completely empty) to the study nurse at each visit occurring at the SHCS center. The study sites will maintain adequate documentation of the ARV drug distribution. Sample documentation will be provided by the Sponsor-Investigator. This applies to the interventional product as well as the comparators.

8.9 Return of Study Drug

Returned drugs bottles (full, partially empty and completely empty) will be registered in individual patient's files.

9. STUDY ASSESSMENTS

9.1 Study flow chart(s) / table of study procedures and assessments

	Screening	Year 1					
	-4 weeks to -1 week	Day 0	Week 6	Week 12	Week 24	Week 36	Week 48
Eligibility screen [1]	X	X					
Written informed consent	X						
Demographics	X						
HIV-related data and medical history [2]		X					
Concomitant medications [3]		X	X	X	X	X	X
Full physical examination by physician [4]		X			X		X
Allocation (double randomization)		X					
Start DTG + FTC OR remain on standard cART		X					
Start patient-centered monitoring OR remain on standard monitoring		X					
Adherence check		X	X	X	X	X	X
Adverse events check		X	X	X	X	X	X
Drug distribution		X	X	X	X	X	X
HIV-RNA in plasma + sample storage	X[5]	X[β]	X[β]	X[β]	X[β]	X[β]	X[β]
HIV-DNA in plasma (in batch)		X					X
CD4 count*	X			X*	X*	X*	X

Columns with purple background represent visits which need to be performed at site

[1] At screening visit: patient chart review for exclusion and inclusion criteria. At day 0: review of laboratory assessments made at the screening visit and completion of the eligibility checklist.

[2] At day 0: HIV-1 infection, date of HIV-1 diagnosis, years of HIV-1 suppression, nadir CD4 count, zenith HIV-1 RNA, previous genotype, ongoing hepatitis B and C, other co-morbidities. All other visits: updated medical history including changes or additions to diagnoses, diseases and medications will be done in the frame of the concomitant medication and the adverse event check.

[3] Prohibited: carbamazepine, oxcarbamazepine, phenytoin, phenobarbital, St John's wort and rifampicin.

[4] Full physical examination by study nurse & medical doctor: vital signs, anthropometric measurements, cardiovascular, respiratory and gastrointestinal systems; central nervous and skin systems as deemed necessary.

[5] No sample storage on screening visit

Full blood count*	X			X*	X*	X*	X
Safety serum chemistry [6] *	X		¥µ	X*µ	X*µ	X*µ	X
Urine Beta HCG pregnancy test in women with childbearing potential if applicable	X				X		X
HIV RNA in plasma and sample storage		X	**(at any time if HIV-RNA is ≥ 100 copies)**				
Genotypic resistance test (real time) and pharmacogenetic analysis (en batch)			**(at any time if HIV-RNA is ≥ 100 copies on two consecutive measurements)**				
DTG and FTC plasma concentration			**(at any time if HIV-RNA is ≥ 100 copies, if twice consecutively only at first time)**				
Health-related quality of life questionnaire PROQOL-HIV<br>Visual analog scale		X<br>X		X<br>X			X<br>X
Assess willing for change of monitoring option / satisfaction if patient randomised in the patient-centered monitoring arm				X	X	X	X
Health related costs assessment							X

[6] Safety serum chemistry includes CK, creatinine, ASAT, ALAT, gamma-GT, alkaline phosphatases; glucose and lipid profile.

β Patients with HIV-RNA between 20 and 99 copies/ml will benefit from another HIV-RNA measurement after 6 4 weeks (± 5 days).

* CD4-count, full blood count and safety serum chemistry will be performed on a 3-monthly basis for patients randomized in the standard monitoring arm. Patients randomized in the patient-centered monitoring arm will benefit from these routine tests on a yearly basis (i.e. at day 0 and week 48).

¥ Patients new to DTG will benefit from CK measurement and from hepatic safety serum tests, i.e. ASAT, ALAT, gamma-GT, alkaline phosphatases, at week 6 whatever monitoring arm.

µ Diabetic patients under concomitant metformine and dolutegravir will benefit from blood glucose and creatinine measurements at weeks 6, 12, 24 and 36 whatever monitoring arm.

9.2 Assessments of outcomes

9.2.1 Assessment of Primary Outcomes

- Viral load in plasma: HIV-1 RNA level in the plasma will be measured at every study-visit. HIV-1 RNA levels will be quantified using PCR with a limit of detection of 20 copies/ml or less at local virology laboratories. The same assays are being used for and recognised by the SHCS.
- Costs: costs will be measured starting from the baseline visit for the complete study period and from the perspective of the health care provider. Costs will be obtained from health-care insurance of the participants and from trial accounting and collected at week 48. We will consider the following costs:
  - Costs of outpatient medical consultations (HIV and non-HIV medical consultations);
  - Costs of other consultations (non-medical consultations);
  - Costs of antiretroviral drugs;
  - Costs of other drugs;
  - Costs of laboratory tests;
  - Costs of diagnostic tests other than laboratory;
  - Costs of hospitalization;
  - Costs related to the study;
  - Other costs.

9.2.2 **Assessment of Secondary Outcomes**

- Proportion of patientswith HIV-RNA <50 cp/ml at week 48 by FDA snapshot. The following assumptions will be done:
  a. Success is considered as HIV-RNA < 50 cp/ml in the shanpshot time window;
  b. Failures will be defined as one of the following categories, i.e.:
     i. HIV-RNA $\geq$ 50 cp/ml;
     ii. Change of therapy
     iii. Discontinuation of therapy due to lack of efficacy;
  c. No virological data will be defined as one of the following categories, i.e.:
     i. Discontinued due to adverse event or death;
     ii. Discontionuation of therapy due to consent withdrawal or loss to follow-up or other reason;
     iii. Patients that are on study but have missing data in shapshot time window;
  d. Shapshot time window is 48 wkks after the baseline visit +/- 3 weeks (i.e. window size is half of the duration of the time between study visits);
- Loss of future drug options will be assessed in patients failing treatment, i.e. patients who will have 2 consecutive HIV RNA measurements ≥100 copies/ml. For these patients, genotype resistance testing will be performed at the SHCS center according to their usual practice. Plasma samples will be collected at each visit, processed and stored at -80°C at local virology laboratories. The same processing and storage conditions will be used as for SHCS samples. Genotype resistance interpretation will be centrally repeated by the Laboratory of Virology at HUG at the end of the study.
- CD4 cell count change: measurement of the CD4/CD8 fraction at local immunology or virology laboratories.
- HIV-DNA level change: HIV-DNA level will be measured in batch by a digital droplet PCR (ddPCR) assay using the RAINDANCE system. PBMC samples will be stored at randomization and at week 48 visit and the assay will be performed retrospectively in the Division of Infectious Diseases and Hospital Epidemiology, University hospital Zurich
- Lipidic profile changes: measurement of total cholesterol, LDL-cholesterol, HDL-cholesterol and triglycerides at local chemistry laboratories.
- Glocose level changes: measurement of glucose at local laboratories.

- Framingham-calculated cardiovascular risk changes: calculation using the Framingham 10 year CHD risk calculator by MDCalc: http://www.mdcalc.com. A printout of the result should be filed with the source documents.
- Glomerular function rate changes: measurement of plasma creatinine at the sites' local chemistry laboratories and calculation according to the CKD-EPI (Chronic Kidney Disease Epidemiology Collaboration) equation using the following online calculator: http://www.qxmd.com/calculate-online/nephrology/ckd-epi-egfr. A printout of the result should be filed with the source documents.
- Occurence of adverse events: documentation in the electronic CRF by the study nurse or other (e.g. study coordinator, research associate).
- Occurence of serious adverse events (SAE): documentation in the electronic CRF by the study nurse or other (e.g. study coordinator, research associate).
- Occurence of CNS adverse events: documentation in the electronic CRF (log AE) by the study nurse or other (e.g. study coordinator, research associate).
- Presence of CNS symptoms: documentation in the electronic CRF (week 2 and 6 CNS questionnaire) by the study nurse or other (e.g. study coordinator, research associate).
- Weight changes: documentation in the electronic CRF by the study nurse or other (e.g. study coordinator, research associate).
- Health-related quality of life: assessment using the PROQOL-HIV instrument (87, 88) and a visual analog scale. These assessments will be performed at baseline, week 12 and week 48 by the study nurse/physician. The PROQOL-HIV questionnaire can be filled-in directly by the patient. Documentation on paper source document and transfer into the electronic CFR by the study nurse or other (e.g. study coordinator, research associate).
- Patient's monitoring satisfaction. Assessment using:
    - Monitoring choice, for patients randomized in the patient-centered monitoring arm);
    - Visual analogue scale (for all patients regardless of the randomizato arm),
    - Proportions of patients in the patient-centered monitoring arm expressing the the willingness to change of monitoring option
- Patient's treatment satisfaction: assessing using visual analogues scale, patient's preferences about treatment, ARV regimen chosen for the post-study period and reason for change in case of change.
- Study satisfaction: assessment using 7 questions discussed at week 48.

9.2.3 Assessment of other Outcomes of Interest

- Adherence will be assessed at each study visit by questionnaire and pill count as described in section 8.4 for all patients. Adherence will also be assessed via a DTG + FTC plasma concentration at weel 48 (performed retrospectively) for patient on an ARV regimen containing at least DTG + FTC.
- DTG and FTC drug plasma concentration levels will be determined by liquid chromatography coupled with tandem mass spectrometry (LC-MS/MS). This method is available at the Laboratory of clinical pharmacology at CHUV, Lausanne, Switzerland.
- DTG pharmacogenetic analysis will be performed at the Laboratory of clinical pharmacology at CHUV, Lausanne, Switzerland, only for patients included in the SHCS and who have signed a consent form for genetical analysis within the SHCS.
- Extra-visits outside scheduled study visits will be recorded in the electronic CRF by the study nurse or other (e.g. study coordinator, research associate) with the date and reason of consultation. Extra visits are all medical and non-medical visits reimboursed by the patient's health insurance.
- Study-related data, such as study drug distribution and return, randomization will be collected by the study nurse or other (e.g. study coordinator, research associate) as per protocol and recorded on study source documents and entered into the electronic CRF.
- Demographic data will be retrieved from the SHCS or the patient's medical file and will include: age, sex, ethnicity and level of education.
- HIV related data and medical history data will be retrieved from the SHCS or the patient's medical file and will include: date of HIV-1 infection (first documented positive HIV-1 serology), duration of viral suppression, nadir CD4 count, previous genotype, prior AIDS events (CDC C events), co-infection with hepatitis B or C, other ongoing medical conditions at baseline (i.e. diabetes mellitus, hypertension, etc.).

9.2.4 Assessment of Safety Outcomes

9.2.4.1 Adverse Events

At every study visit starting from the randomization in the trial, i.e. from the baseline visit, we will record adverse event information (i.e. type of event, date of onset, duration, final outcome, action to be taken, assessment of severity, relationship with study drug(s) and/or procedure). For the definition and procedures, we are reffering to section 10. CNS side effects will be recorded using a standardized questionnaire at week 2 (by phone call) and week 6 among patients new to DTG drug and reported as adverse events as well. Assessment of adverse events will be standardized in the CRF.

9.2.4.2 Laboratory parameters

Efficacy laboratory such as HIV-1 RNA level will be quantified at every visit using PCR with a limit of detection of 20 copies/ml or less at the sites' virology laboratories. Plasma samples will also be collected at every visit, processed and stored at -80°C as per SHCS samples. In case of HIV-RNA measurement ≥ 100 copies/ml, stored plasma sample will be sent to the Laboratory of clinical pharmacology of the CHUV for DTG and FTC plasma concentration. In case of virological failure, stored plasma will be used for genotypic resistance testing. Otherwise, cells and plasma samples are stored at least until the publication of the final manuscript. Detailed instructions for sample collection, storage and shipment will be provided by the sponsor in the written standard operating procedure.

Safety laboratory (i.e. creatinine, CK, ASAT, ALAT, gamma-GT, alkaline phosphatases, glucose and lipid profile), full blood count and CD4/CD8 cell count will be measured at the local clinical chemistry, haematology and immunology/virology laboratories. Certificates and normal value ranges will be available at each study site in the investigator file. Laboratory abnormalities that are listed in the Adverse Event list should be graded accordingly (see details in the section 10).

9.2.4.3 Vital signs

Blood pressure and heart rate will be measured after 10 minutes resting in a sitting position. Temperature will be collected and recorded in the CRF. Physical examination will include anthropometric measurements (weight and height), assessement of the cardiovascular, respiratory and gastrointestinal systems, as well as skin and central nervous systems as deemed necessary by the study physician.

9.2.5 Assessments in participants who prematurely stop the study

Participants who prematurely stop the study will be asked to come in and complete the study procedures of the week 48 visit. Procedures are described in section 10.

9.3 Procedures at each visit

9.3.1. Screening, visit 1 and informed consent (Week -4 to week-1) at SHCS center

Participants must give their written consent by signing the informed consent form prior to any study specific procedure. Results from a blood test performed before the screening visit is accepted for the Simpl'HIV trial if a patient is included in the SHCS and if the time window of -7 to -28 days to the baseline visit is respected. In such cases data should be entered in the database as part of the screening visit.

Screening visit can be done by the study nurse only (the presence of a medical doctor is not obligatory).

Clinical assessments:

- Demographics related data.

Laboratory assessments:

- HIV-1 RNA in the plasma;
- CD4 count;

- Full blood count;
- Safety serum chemistry: CK, creatinine, ASAT/ALAT, gamma-GT, alkaline phosphatases, glucose and lipid profile;
- Urinary β-HCG in women of childbearing potential.

Eligibility criteria will be checked by the study physician following the results of the screening visit.
If some of the laboratory assessments are not done at the screening visit and this does not impact patient's eligibility, these assessments should be performed at baseline visit. In such cases the results will be entered in the database as part of the screening visit.

9.3.2. Baseline, visit 2 (Day 0) at SHCS center

All assessments made at the screening visit must be reviewed and the eligibility criteria check-list completed by the study physician in order to randomise the patient. The randomisation will be performed by sending a completed electronic randomisation request from the study site in secuTrial. Details will be provided by the sponsor in the Standard Operating Procedure.

The randomisation confirmation will be filed in the study documents. The participant's assigned treatment groups and corresponding randomisation numbers will be recorded in the CRF.

Clinical assessments:

- Eligibility check;
- Questionnaires completion by the patient: visual analog scale, patient's monitoring satisfaction;
- Adherence check;
- Medical history including diagnosis, diseases and concomitant medications. Any ongoing conditions that could be recorded as adverse events during the course of the study should be recorded;
- Physical examination and vital signs;
- Start DTG+FTC dual maintenance therapy OR remain on standard cART;
- Start patient-centered monitoring OR remain on standard monitoring;
- Documentation of alternative option(s) chosen for patient-centered monitoring if randomised in patient-centered monitoring;
- Study drug distribution and documentation (DTG + FTC or standard cART).

Laboratory assessments:

- HIV-1 RNA in plasma. If the baseline visit is performed within 7 days from the HIV-RNA measurement used for the screening visit, it is not mandatory to repeat the HIV-RNA measurement at the baseline visit. In such cases, HIV-RNA result used for the screening visit will be entered as well for the baseline visit in the database.
- Sample plasma and cells sample storage. If a venipuncture is planned at the baseline visit only for storages and the patient is included into the SHCS, plasma and cell sample storage can be drown at the screening visit.
- HIV-1 DNA from PBMCs by ddPCR (performed retrospectively)
- DTG-related pharmacogenetics host profile will be performed for all patients on DTG. This analysis will be performed retrospectively according to funding availability and only for patients included in the SHCS and who have signed a consent form for genetic studies.

9.3.3. Week 2 (± 3 days)

Clinical assessments:

- CNS adverse event questionnaire for patients new to DTG (phone call with study nurse/physician for all subjects, regarless of the monitoring arm after randomisation).

9.3.4. Week 6 (± 7 days)

Clinical assessments:

- Adherence check by the study nurse/physician (face-to-face visit or by phone call);
- Recording/update of concomitant medications (face-to-face visit or by phone call);
- Adverse events recording (face-to-face visit or by phone call);

- CNS adverse event questionnaire for patients new to DTG (face-to-face visit or by phone call);
- Study drug distribution (DTG + FTC or standard cART) at study center, chosen pharmacy or by mail depending of study arm and option(s) chosen by the patient.

Laboratory assessments (at SHCS center or peripheral laboratory):

- HIV-1 RNA in plasma;
- Plasma sample storage;
- Dolutegravir and emtricitabine plasma concentration if HIV-1 RNA measurement ≥ 100 copies/ml in plasma;
- Genotypic resistance test if 2 consecutive HIV-1 RNA measurements ≥ 100 copies/ml in plasma;
- Pharmacogenetic analyses if 2 consecutive HIV-1 RNA measurements ≥ 100 copies/ml in plasma only for patients included in the SHCS and who have signed a consent form for genetic studies;
- CK, ASAT/ALAT, gamma-GT and alkaline phosphatases if patient new to DTG drug;
- Glucose and creatinine if patient under DTG and metformine.

For patients randomised in the patient-centered monitoring arm, study drug distribution and laboratory assessments can be done before clinical assessments but within the time window of the visit.

9.3.5. Weeks 12 and 36 (± 7 days)

Clinical assessments:

- Adherence check by the study nurse/physician (face-to-face visit or by phone call);
- Recording/update of concomitant medications (face-to-face visit or by phone call);
- Adverse events recording (face-to-face visit or by phone call);
- Questionnaires completion: visual analog scale, PROQOL-HIV, (face-to-face visit or by phone call);
- Study drug distribution (DTG + FTC or standard cART) at study center, chosen pharmacy or by mail depending of study arm and option(s) chosen by the patient;
- Patient’s monitoring satisfaction.

Laboratory assessments (at SHCS center or peripheral laboratory):

- HIV-1 RNA in plasma
- Plasma sample storage;
- Dolutegravir and emtricitabine plasma concentration if HIV-1 RNA measurement ≥ 100 copies/ml in plasma;
- Genotypic resistance test if 2 consecutive HIV-1 RNA measurements ≥ 100 copies/ml in plasma;
- Pharmacogenetic analyses if 2 consecutive HIV-1 RNA measurements ≥ 100 copies/ml in plasma only for patients included in the SHCS and who have signed a consent form for genetic studies;
- CD4 count, full blood count, safety serum chemistry (CK, creatinine, ASAT/ALAT, gamma-GT, alkaline phosphatases, glucose and lipids) if patient randomised in standard monitoring arm;
- Glucose, and creatinine if patient under DTG and metformine and patient-centered monitoring arm.

For patients randomised in the patient-centered monitoring arm, study drug distribution and laboratory assessments can be done before clinical assessments but within the time window of the visit.

9.3.6. Week 24 (± 7 days) at SHCS center

Clinical assessments:

- Adherence session by the study nurse;
- Recording/update of concomitant medications;
- Adverse event recording;
- Physical examination and vital signs;
- Study drug distribution and return (DTG + FTC or standard cART) according to site standard practice;
- Patient’s monitoring satisfaction (possibility to change patient-centered monitoring options).

Laboratory assessments:

- HIV-1 RNA in plasma;
- Plasma sample storage;
- Dolutgeravir and emtricitabine plasma concentration if HIV-1 RNA measurement ≥ 100 copies/ml in plasma
- Genotypic resistance test if 2 consecutive HIV-1 RNA measurements ≥ 100 copies/ml in plasma;
- Pharmacogenetic analyses if 2 consecutive HIV-1 RNA measurements ≥ 100 copies/ml in plasma only for patients included in the SHCS and who have signed a consent form for genetic studies;
- CD4 count, full blood count, safety serum chemistry (CK, creatinine, ASAT/ALAT, gamma-GT, alkaline phosphatases, glucose and lipids) if patient randomised in standard monitoring arm;
- Glucose, and creatinine if patient under DTG and metformine and patient-centered monitoring arm.
- Urinary β-HCG in women of childbearing potential.

9.3.7. Week 48 (±21 days) or study discontinuation visit at SHCS center:

Clinical assessments:

- Questionnaires completion: visual analog scale, PROQOL-HIV
- Adherence session by the study nurse;
- Recording/update of concomitant medications;
- Adverse event recording;
- Physical examination and vital signs;
- Study drug return (DTG + FTC or standard cART).
- Patient's monitoring satisfaction.

Laboratory assessments:

- HIV-1 RNA in plasma;
- CD4 count;
- Full blood count;
- safety serum chemistry: CK, creatinine, ASAT/ALAT, gamma-GT, alkaline phosphatases, glucose and lipid profile;
- Plasma and cells sample storage;
- HIV-1 DNA from PBMCs by ddPCR (performed retrospectively);
- Dolutegravir and emtricitabine plasma concentration for patients on ARV regimen containing at least DTG and FTC (performed retrospectively);
- Dolutegravir and emtricitabine plasma concentration if HIV-1 RNA measurement ≥ 100 copies/ml in plasma
- Genotypic resistance test if 2 consecutive HIV-1 RNA measurements ≥ 100 copies/ml in plasma;
- Pharmacogenetic analyses if 2 consecutive HIV-1 RNA measurements ≥ 100 copies/ml in plasma only for patients included in the SHCS and who have signed a consent form for genetic studies (performed retrospectively);
- Urinary β-HCG in women of childbearing potential.

Costs assessments

10. SAFETY

10.1 Drug studies

The Sponsor's SOPs provide more detail on safety reporting.

During the entire duration of the study, adverse events (AE) and serious adverse events (SAEs) are collected, fully investigated and documented in source documents and case report forms (CRF). Study duration encompassed the time from when the participant has been randomized in one study arm, i.e. starting from the baseline visit until the last protocol-specific procedure has been completed, including a safety follow-up period when needed.

10.1.1. Definition and assessment of (Serious) Adverse Events and other safety related events

An **Adverse Event (AE)** is any untoward medical occurrence in a patient or a clinical investigation participant administered a pharmaceutical product and which does not necessarily have a causal relationship with the study procedure. An AE can therefore be any unfavourable and unintended sign (including an abnormal laboratory finding), symptom, or disease temporally associated with the use of a medicinal (investigational) product, whether or not related to the medicinal (investigational) product.

Adverse Events that need to be reported in this study are listed in the Adverse Event list (see Appendix 6). This list will be used starting from the baseline visit to confirming the absence or presence of any listed adverse event. Spontaneous, non-serious Adverse Events that are not listed in the Appendix 6 list will be collected in the patient's medical file according to local usual practice but will not be reported into the study-related document. Laboratory abnormalities listed in the Adverse Event list, will be reported as Adverse Events if they are at least a grade 3 and if they appear after randomization in the study (i.e. starting from the baseline visit). In case of laboratory abnormality already present befor the randomisation, this will be considered as an adverse event only in case of worsening.

A **Serious Adverse Event (SAE)** is classified as any untoward medical occurrence that:

- results in death,
- is life-threatening,
- requires in-patient hospitalization or prolongation of existing hospitalisation,
- results in persistent or significant disability/incapacity,
- results in a congenital anomaly/birth defect, or
- is an important medical events that may not be immediately life-threatening or result in death, or require hospitalisation, but may jeopardise the patient or may require intervention to prevent one of the other outcomes listed above should also usually be considered serious. Examples of such events are intensive treatment in an emergency room or at home for allergic bronchospasm, blood dyscrasias or convulsions that do not result in hospitalisation, or development of drug dependency or drug abuse.

SAEs should be followed until resolution or stabilisation. See details in section 10.1.3.

Assessment of Causality of (Serious) Adverse Events and other safety related events

The Study Investigator makes a causality assessment of the event to the study drug and study procedure using the categories based on the ICH E2A guidelines:

Relationship	Description
Certain	Temporal relationship +<br>Improvement after dechallenge +<br>Recurrence after rechallenge<br>(or other proof of drug cause)
Probable	Temporal relationship + Improvement after dechallenge<br>No other cause evident

Possible	Temporal relationship<br>Other cause possible
Unlikely	Any assessable reaction that does not fulfil the above conditions
Excluded	Causal relationship can be ruled out
Not assessable	Not assessable

In case of an Adverse Event at least possibily related to the study drug, DTF and FTC plasma concentration measurement can be performed at trating fisician discretion as standard of care.

Unexpected Adverse Drug Reaction

An unexpected adverse drug reaction is an adverse reaction, the nature or severity of which is not consistent with the applicable product information (e.g. Investigator's Brochure for drugs that are not yet approved and Product Information for approved drugs, respectively).

Suspected Unexpected Serious Adverse Reactions (SUSARs)

A SUSAR is an unexpected adverse drug reaction that is at least possibly related to the IMP or the study procedure. The Sponsor-Investigator receives the SAE evaluation about seriousness and causality from the Study Investigator. The Sponsor-Investigator evaluates any SAE that has been reported regarding expectedness. If the event is at least possibly related to the investigational product and is both serious and unexpected, it is classified as a SUSAR.

Assessment of Severity of (Serious) Adverse Events and other safety related events

Assessment of severity of events will be performed on the basis of the DAIDS Table for Grading the Severity of Adult and Pediatric Adverse events (version 2.0, November 2014) (95).

10.1.2. Reporting of (Serious) Adverse Events and other safety related events

Reporting of SAEs

All SAEs must be reported immediately and within a maximum of 24 hours to the Sponsor-Investigator of the study. The Sponsor-Investigator will evaluate the expectedness of the SAE.

SAEs resulting in death are reported to the leading Ethics Committee by the Sponsor-Investigator within 7 days.

Reporting of SUSARs

A SUSAR must be reported to the leading Ethics Committee via the Sponsor-Investigator within 7 days if the event is fatal, or within 15 days for all other events.

The Sponsor-Investigator must inform all investigators participating in the clinical study of the occurrence of a SUSAR within the same time frame.

Reporting of Safety Signals

All suspected new risks and relevant new aspects of known adverse reactions that require safety-related measures, i.e. so called safety signals, must be reported to the Sponsor-Investigator within 24 hours. The Sponsor-Investigator must report the safety signals within 7 days to the leading Ethics Committee and all investigators participating in the clinical study.

Reporting and Handling of Pregnancies

Pregnant women are not eligible for the study. A pregnancy occurring during the study does not lead to a study withdrawn. Any pregnancy occurring during the treatment phase of the study and within 30 days after discontinuation of study medication will be reported to the Sponsor-Investigator within 24

hours. The course and outcome of the pregnancy will be followed up carefully, and any abnormal outcome regarding the mother or the child will be documented and reported to the Sponsor-Investigator. Pregnancies and their outcomes should be followed until resolution or stabilisation. Participants with ongoing pregnancy at week 48 or study discontinuation visit will be further followed up until pregnancy resolution. In case of newborn health problem, the duration of the follow-up can be extended.

Periodic reporting of safety

An annual safety report will be submitted once a year to the leading Ethics Committee by the Sponsor-Investigator. This annual safety report will contain information from all study sites. The Sponsor-Investigator will prepare the annual safety report and distribute it to the participating Investigators.

10.1.3 Follow up of (Serious) Adverse Events and other safety related events

Participants with ongoing AE, SAEs, SUSARs or pregnancy at week 48 or at study discontinuation visit will be further followed until either final outcome or 6 weeks (+/- 5 days) after the last patient last visit. Ongoing pregnancies at 48 week visit or at study discontinuation visit will be further followed-up until pregnancy resolution. Extension of this duration can be proposed for event of particular interest for the study. Follow up may include but is not limited to physical examination, laboratory tests, vital signs, telephone calls. Outcomes and resolution of events will be recorded in the Case Report Forms. In case of lost to follow up, efforts will be made to contact the patient or to ascertain the vital status of the participant.

11. STATISTICAL METHODS

11.1 Hypotheses

- HA1: DTG + FTC maintenance dual therapy is non-inferior to cART in maintaining plasma HIV viral load <100 copies/ml throughout 48 weeks in virologically suppressed patients.
- HO1 (tested): DTG + FTC maintenance dual therapy is inferior to cART by 12% in maintaining plasma HIV viral load <100 copies/ml throughout 48 weeks in virologically suppressed patients.

- HA2: Patient-centered monitoring reduces health-costs compared to standard monitoring.
- HO2 (tested): Patient-centered monitoring does not reduce health-costs compared to standard monitoring.

11.2 Determination of Sample Size

The first sample size calculation was based on the primary outcome for the comparison between DTG + FTC dual therapy and standard cART, i.e. the comparison between the proportions of patients in the two arms maintaining virologic suppression, using a two-sample proportion test (96). The following assumptions were made:

- Power 80%
- Significance level 0.025 (one-sided Z test of non-inferiority)
- 97% of patients with conventional cART will be virologically suppressed at week 48 (<100 copies/ml)
- 94% of patients on DTG + FTC dual therapy will be virologically suppressed at week 48 (<100 copies/ml)
- Non-inferiority margin of 12% (as in previous non-inferiority trials)(97-104). We chose 12% noninferiority margin because there is no difference in terms of resistance acquisition between 10% and 22% virological failure.

Sample size of 83 patients in each group (total sample size, 166 patients) to achieve 80% power to detect a non-inferiority margin difference between the group proportions of -0.12 was calculated. The power was computed for the case when the actual cART group proportion is 97% and the actual DTG + FTC dual therapy group proportion is 94%. To allow for at least 10% loss to follow-up and also for the randomisation in two steps in this 1:1:1:1 trial, we will aim to recruit **a total of 184 patients**.

The second sample size calculation was based on the primary outcome for the comparison between simplified monitoring and standard monitoring, i.e. the comparison between mean direct costs in the two arms (105). The following assumptions were made:

- Significance level 0.05 (two-sided t-test)
- Direct costs are CHF 25,000 per year (standard deviation (SD) CHF 8,000) with conventional monitoring
- SDs are equal in the two groups (CHF 8,000)

With sample sizes of 92 patients in each group, we will have approximately 55% power to detect a 10% difference in costs, and 99% power to detect a 25% difference in costs. We will also have substantial power to detect small-to-moderate differences in many of the secondary outcomes, e.g. changes in CD4 cell counts or health-related quality of life. The exceptions are rare outcomes such as adverse events, resistance or clinical complications for which the statistical power to detect differences will be low.

11.3 Statistical criteria of termination of trial

This is an open label study. The laboratory results that define treatment failure (HIV-RNA) of all participants included will be available within a week to the investigators. A detailed report including a summary of Serious Adverse Events, grade 3/4 Adverse Events, laboratory toxicities as well as virological failures, pregnancies and safety signals will be sent to the DSMB on a 6-monthly basis.

To protect the safety of the study participants, a safety interim analysis on the first 100 patients randomized will be done once reached the end of the time window for their 24 weeks study visit. The following data will be reviewed:

Safety data:

- Participant recruitment, accrual, retention and withdrawal information
- Adverse events (AEs) and serious adverse events (SAEs)
- Pregnancies
- Laboratory values
- Additional virological tests performed outside study schedule
- Any other safety-supporting data requested by the DSMB

Efficacy data:

- HIV-RNA data (primary endpoint measure)
- Genotype resistance test data (secondary endpoint)

We will also convene the DSMB in the following situations:

(1) if > 2 patients experienced virological failure and a new resistances on DTG + FTC dual therapy before the planned interim safety analysis is done;
(2) if new published or unpublished data show any major inconvenience of testing a DTG + FTC dual therapy arm.

The DSMB will then give recommendations regarding the continuation, modification or termination of the trial.

Stopping rules:

The DSMB may recommend stopping the trial for the following reasons:

- The data show a significantly increased risk of serious adverse events in one of the study groups;
- On the basis of a positive efficacy result only when the data are truly compelling and the risk of a false positive conclusion is acceptably low. The trial will only be stopped for positive efficacy if a strong statistically and clinically significant difference in virological success is seen between treatment arms;
- If interim data suggest that the simplified treatment/monitoring arm under study is of no benefit (trend indicating clear inferiority of the simplified treatment/monitoring arm), or that accrual rates are too low and/or that noncompliance is too great to provide adequate power for identifying the specified benefit, the DSMB may consider whether continuation of the study is futile and may recommend termination on this basis;
- It becomes clear that successful completion of the study is not feasible (e.g. there is an excess of patient dropout, missing data, lack of recruitment etc).

11.4 Planned Analyses

11.4.1 Datasets to be Analysed, Analysis Populations

As recommended by the CONSORT statement (106), two analysis sets will be performed: per-protocol (PP) and intention-to-treat (ITT).

For PP analysis, we will exclude patients for the following reasons: patients who discontinue treatment prematurely (< 47 weeks), unless virological failure occurred before, patients with a treatment adherence of less than 80% while on treatment, those who do not have HIV-1 RNA results at week 48 (+/-21 days), unless virological failure occurred before, or patients with major protocol deviation(s) (i.e. any failure to obtain informed consent, violation of any inclusion criteria, not receiving the allocated treatment or monitoring). Per-protocol and intention-to-treat analyses should reach the same conclusion to consider DTG + FTC as non-inferior to standard treatment.

For ITT analysis, all randomized patients will be analysed in the allocated group regardless of any protocol violations.

All drop outs will be described and reasons for their discontinuation will be given where possible.

11.4.2 Primary analysis

As a first step in this factorial trial, we will test for an interaction between the type of drug treatment and the monitoring type in order to assess if treatment effect is different depending on the type of monitoring. If the test is non-significant we will combine the two types of monitoring for the drug therapy comparison and the two drug therapies for the monitoring comparison. Otherwise, the trial will be analysed as a four-arm trial with a hierarchical testing approach.

For the primary comparison of the primary outcome between the two treatment arms, we will obtain the proportion of patients with viral suppression for 48 weeks in each arm, and compute a Mantel-Haenszel risk difference (DTG+FTC - cART) stratified for the monitoring type with a two-sided 95% confidence interval (107). If the lower confidence limit is higher than -12% we will conclude non-inferiority. If the lower confidence limit is below -12% we will conclude that DTG + FTC is potentially inferior to cART.

For the secondary comparison of the primary outcome between the two monitoring arms, we will calculate a Mantel-Haenszel test statistics and Mantel-Haenszel risk difference (DTG+FTC - cART) stratified for the type of treatment.

Secondary categorical outcomes (proportions of virological suppression <50 copies/mL both as a standard proportion and as FDA snapshot, of loss of future treatment options, of adverse events, of serious adverse events, of CSN adverse events, of CNS symptoms) between the DTG+FTC and cART groups, and between the patient-centered and standard monitoring groups will also be analysed using the Mantel-Haenszel test statistics and Mantel-Haenszel risk difference (DTG+FTC - cART) stratified for the type of monitoring and type of treatment, respectively.

For continuous outcome variables (CD4 count, HIV-DNA level, total cholesterol, high-density lipoproteine, triglycérides, low density lipoprotein-cholesterol, glucose, Framingham-calculated cardiovascular risk, glomerular function rate, weight, health-related quality of life, treatment satisfaction), we will compare the mean values at 48 months between the DTG+FTC and cART treatment groups and between the patient-centered and standard monitoring groups using linear regression adjusted for the type of monitoring and type of treatment, respectively.

Time to loss of virological response (TLOVR) will be graphically depicted by Kaplan-Meier curves, and evaluated by a univariable and multivariate Cox proportional Hazard model, adjusted for stratified for the type of monitoring and type of treatment.

Predictors of virological failure (nadir CD4 count, baseline HIV-DNA, zenith HIV-RNA, years since HIV infection, years of viral suppression, CD4/CD8 ratio, age, DTG and FTC plasma concentrations, pharmacogenetic analysis, any other relevant clinical or laboratory parameters) will be assessed using forward stepwise multiple logistic regression.

Economic analyses will be performed from the health provider perspective. A multiple linear regression model will be performed to compare the mean direct costs between the two monitoring strategies after adjustment for the treatment arm.

All analyses will be performed in the PP dataset and in the ITT dataset.

11.4.3 Secondary analysis

For categorical outcomes, we will additionally calculate relative risk differences between groups. We will calculate odds ratios from multiple logistic regression models adjusting for the type of monitoring and type of treatment as appropriate. For repeatedly measured categorical outcome variables (virological suppression <50 copies/mL), we will consider a mixed effects logistic regression with a random intercept on the subject to account for within-patient correlations.

For repeatedly measured continuous outcome variables, we will also consider all measurements in a linear mixed effects regression model to account for within-patient correlations. Finally, we will analyse development over time by exploring the functional relationship between outcome and time using fractional polynomials and a treatment-by-time interaction term.

Moreover, we will evaluate all outcomes in crude analyses not adjusting for type of monitoring or type of treatment. Binary outcomes will be compared by the Chi-squared or Fisher’s exact test, continuous outcomes by the Student t-test.

Cost-effectiveness of treatment arms (incremental cost-effectiveness ratio) will be calculated by taking the difference in mean costs between patient-centered arm and standard monitoring arm, and between DTG + FTC dual therapy arm and cART arm, and dividing them by the difference in time to loss of virological response (TLOVR) between the study arms. Difference in TLOVR will be assessed by comparing the area under the patient-centered and standard monitoring Kaplan-Meier curves, and under the DTG+FTC and cART Kaplan-Meier curves. Generalised lo-rank test will be used to compare times between groups.

11.4.4 Interim analyses

The DSMB will meet for a single interim safety analysis on the first 100 patients randomized once the end of the time window for their 24 weeks study visit is reached. The Sponsor-Investigator and all Study investigators will receive the related DSMB advice blinded to study arm. In addition to the scheduled meetings, extraordinary meetings may be requested by DSMB members, the Sponsor-Investigator, or the Study Scientific Committee at any time. The DSMB will review the safety data and may make recommendations about the conduct of the trial (study interruption) should any safety concerns be identified. The interim analysis will be prepared by an independent, unblinded statistician.

11.4.5 Safety analysis

The proportion of serious adverse events across the different study time-points will be compared between the two treatment arms by mixed logistic regression model with a random effect on the subject after adjustment for the monitoring strategy.

The proportion of drug-related events across the different study time-points will be compared between the two treatment arms by mixed logistic regression model with a random effect on the subject after adjustment for the monitoring strategy.

The proportion of patients experiencing loss of future drug options defined as new intermediate or high-level resistance to one or more drugs to which the patient’s virus was considered sensitive at trial entry will be compared between the two treatment arms by a multiple logistic regression model adjusted for the monitoring strategy.

11.4.6 Deviation(s) from the original statistical plan

A statistical analysis plan will be written before the end of the first year of participants enrolment. It will describe in details the statistical analyses answering the primary research question but also answering secondary research questions. It will detail how data will be described depending on their format. Once data will be available, the statistician in charge of the statistical analyses will apply the statistical analysis plan. Any deviations from the planned analyses will be at least described and justified.

11.5 Handling of missing data and drop-outs

The following strategies will be used to replace missing values at the final study timepoint. If HIV-RNA is available at week 36, the value at time 36 will be considered (last observation carried forward). If no data is available at weeks 36 and 48, we will consider these patients as failures. The same approach will be considered for secondary outcomes. For continuous outcomes, we will exclude patients from the analysis if no data is available at weeks 36 and 48. In the snapshot analysis, patients with missing outcome in the time snapshot window will be considered as failures. Sensitivity analyses will be performed to determine whether the conclusions are sensitive to assumptions about the missing-data mechanism. We will compare patient characteristics between the two groups having or not missing data in order to assess their similarity. If differences are outlined, missing data would be considered not at random and some bias in the estimation of treatment effect would be suspected. If not, we will consider the complete case analysis as valid to estimate the noninferiority of DTG+FTC compared to standard treatment.

12. QUALITY ASSURANCE AND CONTROL

12.1 Data handling and record keeping / archiving

12.1.1 Case Report Forms

All study data will be entered in an Electronic Data Capture (EDC) system by the study nurse or study coordinator or investigator or the local data manager with no double data entry.

CRFs will be kept current to reflect subject status at each phase during the course of the study. Study-related data of the patient will be collected in a coded manner. The names of the patients will not be disclosed. A code (unique) will be attributed to each patient registered.

Persons authorized by the sponsor-investigator to perform data entry or data review will be communicated to the data manager who will provide individual access codes according to the function assigned. CRF data entry authorization will be documented on each delegation log and a list of all authorized persons and their function stored with the data manager.

12.1.2 Source data

Source data must be available at each site to document the existence of the study participants. Source data must include the medical history and treatment of the participant as well as the original documents relating to the study:

- worksheets (as paper CRF) for screening, baseline, week2, week6, week 12, week24, week 36, week 48 visits and extra visit, study drug discontinuation visit and study discontinuation visit);
- logs (adherence log, drug accountability log, adverse event log, concomitant medication log);
- patient diary;
- cost-related letter from the patient's health insurance company.

The following data will be considered as source documents: patient questionnaires, demographic data, visit dates, participation in study and Informed Consent Forms, SAEs, AEs, adherence log, drug accountability log and concomitant medication log. All other data must be available in the participant's hospital chart.

12.1.3 Analysis and Record keeping / archiving

At interim safety analysis and final analysis, data files will be extracted from the database into statistical packages to be analyzed. The status of the database at this time is recorded in special archive tables.

The study database with all archive tables will be securely stored by the CTU, University of Bern. Essential documents, the trial master file as well as the investigator file for Geneva will be stored at the HIV unit, infectious diseases consultation at the HUG. Investigator files of other Swiss sites will be stored at each site separately. All study data must be archived for a minimum of 10 years after study termination or premature termination of the clinical trial.

12.2 Data management

12.2.1 Data Management System

The CRFs in this trial are implemented electronically using a dedicated electronic data capturing (EDC) system (secuTrial). The EDC system is activated for the trial only after successfully passing a formal test procedure. All data entered in the CRFs are stored on a Linux server in a dedicated Oracle database.

Responsibility for hosting the EDC system and the database lies with CTU, University of Bern.

12.2.2 Confidentiality, Data Protection (Data security, access and back-up)

The server hosting the EDC system and the database is kept in a locked server-room. Only the system administrators have direct access to the server. A role concept with personal passwords (site investigator, statistician, monitor, administrator etc.) regulates permission for each user to use the system and database as he/she requires.

All data entered into the CRFs are transferred to the database using Secure Sockets Layer (SSL) encryption. Each data point has attributes attached to it identifying the user who entered it with the exact time and date. Retrospective alterations of data in the database are recorded in an audit table. Time, table, data field and altered value, and the person are recorded (audit trail). A multi-level back-up system is implemented.

12.2.3 Electronic and Central Data Validation

Data is checked by the EDC system for completeness and plausibility. Furthermore, selected data points are cross-checked for plausibility with previously entered data for that participant. In addition, central data reviews will be performed on a regular basis to ensure completeness of the data collected and accuracy of the primary outcome data.

Before database lock the PI will validate the collected data with his signature.

12.3 Monitoring

For quality control of the study conduct and data retrieval, all study sites will be visited on-site by appropriately trained and qualified monitors. Any findings and comments will be documented in site visit reports and communicated to the local Investigator and to the Sponsor as applicable. Investigators at the participating study sites will support the Monitor in his/her activities. Prior to study start (first participant enrolled) a plan detailing all monitoring-related procedures will be developed. All source data and relevant documents will be accessible to Monitors and questions of Monitors are answered during site visits.

12.4 Audits and Inspections

No audits are foreseen by the sponsor-investigator. The study documentation and the source data/documents are accessible to auditors/inspectors (also CEC and CA) and questions are answered during inspections. All involved parties must keep the participant data strictly confidential.

12.5 Confidentiality, Data Protection

Direct access to source documents will be permitted for the purposes of monitoring, audits and inspections. Both ethics committee members and employees must also understand the confidentiality requirements for any information divulged to them. The data generated by this study will be considered confidential by the investigators, except to the extent that it is included in a publication as agreed in the publication policy of this protocol.

All data will be collected without any names and identifying information will not be recorded in transcription of tapes.

The protocol will be accessible to the trial team and all investigators and delegates during and after the course of the study. The dataset will be accessible only to the data manager, the principal investigators, sponsor-investigator and the statistician.

12.6 Storage of biological material and related health data

Plasma samples will be stored until at least the publication of the final manuscript

13. POST-STUDY

The study period ends at the week 48 visit or at the study discontinuation visit.

Antiretroviral treatment and monitoring after the end of the study period

At week 48 or study discontinuation visit, all patients will be offered the possibility to continue their treatment with DTG + FTC dual therapy, or previous-to-study cART regimen, or current cART regimen, or another regimen. This will be decided at the end of study by treating physician in collaboration with the patient. We suggest planning a viral load follow-up 6 weeks after an ARV de-escalating switch or a switch to a different regimen from the previous-to-study cART.

At week 48 or study discontinuation visit, all patients will be offered the possibility to continue their follow-up as standard monitoring or with one or more options from the patient-centered monitoring, according to local possibilities.

The treating physician will schedule follow-up after the end of the study period in any case, frot he following cases:

- AE, SAE, SUSAR still ongoing (see point 10.1.3);
- Pregnancy still ongoing (see point 10.1.2 and 10.1.3);
- HIV-RNA ≥ 20 cp/ml, for which the procedure descriped at point 3.6 applies.

Post-study visits

All subjects having concluded the week 48 study visit will give their informed consent to extend the study follow-up to 3 years with one visit per year (i.e. week 96 and 144 visits) coinciding with a standard-of-care follow-up visit.

Visit 96 and 144 (+/- 30 days)

Clinical assessments:

- ART changes since previous visit and reasons for changes;
- Current treatment satisfaction;
- Monitoring options changes since previous visit and reasons for changes;
- Current monitoring satisfaction;
- Number, date and value of HIV-RNA performed since previous visit;
- Number, date and results of genotypes resistance testing performed since previous visit*;
- Grade 3 or 4 AE, SAE and pregnancy occurrence since previous visit (reported yearly);
- Quality of life questionnaire and visual analogues scale;
- Adherence questionnaire*;
- Patient's weight and blood pressure*.

Laboratory assessments:

- HIV-1 RNA in plasma*;
- CD4 count*;
- Full blood count*;
- Safety serum chemistry*: creatinine, ASAT/ALAT, alkaline phosphatases, glucose and lipid profile.

* Data will be extracted from the SHCS database whenever possible.

Table of post-study procedure and assessments:

	Week 96 (+/- 30days)	Week 144 (+/- 30 days)
ART changes since previous visit and reasons for changes	X	X
Current treatment satisfaction	X	X
Monitoring options changes since previous visit and reasons for changes	X	X
Current monitoring satisfaction	X	X
Number, date and value of HIV-RNA performed since previous visit	X	X
Number, date and results of genotypes resistance testing performed since previous visit*	X	X
Grade 3 or 4 AE,SAE and pregnancy occurrence since previous visit (reported yearly)	X	X
Quality of life questionnaire and visual analogues scale	X	X
Adherence questionnaire*	X	X
Patient's weight and blood pressure*	X	X
HIV-1 RNA in plasma*	X	X
CD4 count*	X	X
Full blood count*	X	X
Safety serum chemistry*: creatinine, ASAT/ALAT, alkaline phosphatases, glucose and lipid profile	X	X

* Data will be extracted from the SHCS database whenever possible

Objectives and Ouctomes

The overall objective of the post-study is to evaluate the long term efficacy, fesability and acceptance both of the DTG+FTC dual strategy and of the options of a patient-centered monitoring as an observation without any intervention.

Secondary Outcomes related to the post-study:

1. Proportion of patients maintaining HIV suppression;
2. Proportion of patients experiencing loss of future drug options;
3. Time to loss of virological response (TLOVR);
4. Change in CD4 cell count from;
5. Change in lipidic profile;
6. Change in glucose profile;
7. Change in Framingham-calculated cardiovascular risk;
8. Change in glomerular function rate;
9. Proportion of patients with an adverse event of grade 3 or 4;
10. Proportion of patients with a severe at least one serious adverse event (SAE);
11. Proportion of patients with a CNS adverse event;
12. Changes in patient weight;
13. Change in patients' health-related quality of life;
14. Assessment of ART changes during post-study;
15. Assessment of patient's treatment satisfaction;

16. Assessment of option for moniroting changes during post-study;
17. Assessment of patient's monitoring satisfaction;
18. Number of viral load performed yearly;
19. Patient adherence to treatment.

Statistical analysis

Secondary Outcomes related to the post-study will be analyzed as desribed in section 11 for the 96 and 144 week time-points.

14. PUBLICATION AND DISSEMINATION POLICY

The final data will be presented at one or more scientific meetings and published in scientific journal(s). No patient data will be presented that could permit identification of any individual study participant. Publication of data derived from this protocol will be supervised by the Sponsor/Investigator and by the Principal Investigator(s)in conjunction with all study investigators. No publication will be made without prior approval of the Sponsor/Investigator and Principal Investigator(s).

15. FUNDING AND SUPPORT

15.1 Funding

This trial has received funding from the Swiss National Science Foundation. The funding application, review and approval documents as well as the approved budget are stored online (on mysnf.ch) and a copy filed in the trial master file.

Any potential additional funding from SHCS or other institution will be notified to the leading Ethic Committee.

15.2 Other Support

The study will be performed within the SHCS network using the local infrastructure of all participating sites.

Nested sub-studies are possible. In such cases the leading Ethics Committee will be notified.

16. INSURANCE

Insurance will be provided by the Geneva University Hospitals. A copy of the insurance confirmation will be filed in each investigator site file and the trial master file. The insurance certificate is filed at the legal office of the HUG.

17. STUDY AGREEMENT

The present Protocol acts as an Agreement between all participating Study Sites and the Sponsor. The Protocol represents a legal document especially for Study Sites with whom the Sponsor does not have a separate Study Site Agreement, such as Zurich.

18. REFERENCES

1. Sculier D, Calmy A. [What's new in HIV in 2014?]. Rev Med Suisse. 2015 Jan 14;11(456-457):148-52.

2. Calmy A, Sculier D. Is maintenance monotherapy the way forward? . Lancet HIV. 2015(Published Online September 15).

3. Ford N, Flexner C, Vella S, Ripin D, Vitoria M. Optimization and simplification of antiretroviral therapy for adults and children. Curr Opin HIV AIDS. 2013 Nov;8(6):591-9.

4. Cahn P, Andrade-Villanueva J, Arribas JR, Gatell JM, Lama JR, Norton M, et al. Dual therapy with lopinavir and ritonavir plus lamivudine versus triple therapy with lopinavir and ritonavir plus two nucleoside reverse transcriptase inhibitors in antiretroviral-therapy-naive adults with HIV-1 infection: 48 week results of the randomised, open label, non-inferiority GARDEL trial. Lancet Infect Dis. 2014 Jul;14(7):572-80.

5. Pulido F, Matarranz M, Rodriguez-Rivera V, Fiorante S, Hernando A. Boosted protease inhibitor monotherapy. What have we learnt after seven years of research? AIDS Rev. 2010 Jul-Sep;12(3):127-34.

6. Paton NI, Stohr W, Arenas-Pinto A, Fisher M, Williams I, Johnson M, et al. Protease inhibitor monotherapy for long-term management of HIV infection: a randomised, controlled, open-label, non-inferiority trial. Lancet HIV. 2015 Oct;2(10):e417-26.

7. Kahlert C, Hupfer M, Wagels T, Bueche D, Fierz W, Walker UA, et al. Ritonavir boosted indinavir treatment as a simplified maintenance "mono"-therapy for HIV infection. AIDS. 2004 Apr 9;18(6):955-7.

8. Antinori A, Arribas J, Fehr J, Girard PM, Horban A, Hill A, et al. The PROTEA trial: darunavir/ritonavir with or without nucleoside analogues, for patients with HIV-1 RNA below 50 copies/mL. J Int AIDS Soc. 2014;17(4 Suppl 3):19525.

9. Margolis DA, Brinson CC, Smith GH, de Vente J, Hagins DP, Eron JJ, et al. Cabotegravir plus rilpivirine, once a day, after induction with cabotegravir plus nucleoside reverse transcriptase inhibitors in antiretroviral-naive adults with HIV-1 infection (LATTE): a randomised, phase 2b, dose-ranging trial. Lancet Infect Dis. 2015 Jul 17.

10. Raffi F, Jaeger H, Quiros-Roldan E, Albrecht H, Belonosova E, Gatell JM, et al. Once-daily dolutegravir versus twice-daily raltegravir in antiretroviral-naive adults with HIV-1 infection (SPRING-2 study): 96 week results from a randomised, double-blind, non-inferiority trial. Lancet Infect Dis. 2013 Nov;13(11):927-35.

11. Walmsley SL, Antela A, Clumeck N, Duiculescu D, Eberhard A, Gutierrez F, et al. Dolutegravir plus abacavir-lamivudine for the treatment of HIV-1 infection. N Engl J Med. 2013 Nov 7;369(19):1807-18.

12. Clotet B, Feinberg J, van Lunzen J, Khuong-Josses MA, Antinori A, Dumitru I, et al. Once-daily dolutegravir versus darunavir plus ritonavir in antiretroviral-naive adults with HIV-1 infection (FLAMINGO): 48 week results from the randomised open-label phase 3b study. Lancet. 2014 Jun 28;383(9936):2222-31.

13. Llibre JM, Pulido F, Garcia F, Garcia Deltoro M, Blanco JL, Delgado R. Genetic barrier to resistance for dolutegravir. AIDS Rev. 2015 Jan-Mar;17(1):56-64.

14. McCormack PL. Dolutegravir: a review of its use in the management of HIV-1 infection in adolescents and adults. Drugs. 2014 Jul;74(11):1241-52.

15. Cahn P, Pozniak AL, Mingrone H, Shuldyakov A, Brites C, Andrade-Villanueva JF, et al. Dolutegravir versus raltegravir in antiretroviral-experienced, integrase-inhibitor-naive adults with HIV: week 48 results from the randomised, double-blind, non-inferiority SAILING study. Lancet. 2013 Aug 24;382(9893):700-8.

16. Eron JJ, Clotet B, Durant J, Katlama C, Kumar P, Lazzarin A, et al. Safety and efficacy of dolutegravir in treatment-experienced subjects with raltegravir-resistant HIV type 1 infection: 24-week results of the VIKING Study. J Infect Dis. 2013 Mar 1;207(5):740-8.

17. Castagna A, Maggiolo F, Penco G, Wright D, Mills A, Grossberg R, et al. Dolutegravir in antiretroviral-experienced patients with raltegravir- and/or elvitegravir-resistant HIV-1: 24-week results of the phase III VIKING-3 study. J Infect Dis. 2014 Aug 1;210(3):354-62.

18. Cavalcanti Jde S, Ferreira JL, Guimaraes PM, Vidal JE, Brigido LF. High frequency of dolutegravir resistance in patients failing a raltegravir-containing salvage regimen. J Antimicrob Chemother. 2015 Mar;70(3):926-9.

19. Chun TW, Fauci AS. HIV reservoirs: pathogenesis and obstacles to viral eradication and cure. AIDS. 2012 Jun 19;26(10):1261-8.

20. Rouzioux C, Richman D. How to best measure HIV reservoirs? Curr Opin HIV AIDS. 2013 May;8(3):170-5.

21. Svicher V, Ceccherini-Silberstein F, Antinori A, Aquaro S, Perno CF. Understanding HIV compartments and reservoirs. Curr HIV/AIDS Rep. 2014 Jun;11(2):186-94.

22. Deng K, Siliciano RF. HIV: Early treatment may not be early enough. Nature. 2014 Aug 7;512(7512):35-6.

23. Tsiara CG, Nikolopoulos GK, Bagos PG, Goujard C, Katzenstein TL, Minga AK, et al. Impact of HIV type 1 DNA levels on spontaneous disease progression: a meta-analysis. AIDS Res Hum Retroviruses. 2012 Apr;28(4):366-73.

24. Valcour VG, Ananworanich J, Agsalda M, Sailasuta N, Chalermchai T, Schuetz A, et al. HIV DNA reservoir increases risk for cognitive disorders in cART-naive patients. PLoS One. 2013;8(7):e70164.

25. Piketty C, Weiss L, Assoumou L, Burgard M, Melard A, Ragnaud JM, et al. A high HIV DNA level in PBMCs at antiretroviral treatment interruption predicts a shorter time to treatment resumption, independently of the CD4 nadir. J Med Virol. 2010 Nov;82(11):1819-28.

26. Vallejo A, Gutierrez C, Hernandez-Novoa B, Diaz L, Madrid N, Abad-Fernandez M, et al. The effect of intensification with raltegravir on the HIV-1 reservoir of latently infected memory CD4 T cells in suppressed patients. AIDS. 2012 Sep 24;26(15):1885-94.

27. Hey-Cunningham WJ, Murray JM, Natarajan V, Amin J, Moore CL, Emery S, et al. Early antiretroviral therapy with raltegravir generates sustained reductions in HIV reservoirs but not lower T-cell activation levels. AIDS. 2015 Feb 27.

28. Boulassel MR, Chomont N, Pai NP, Gilmore N, Sekaly RP, Routy JP. CD4 T cell nadir independently predicts the magnitude of the HIV reservoir after prolonged suppressive antiretroviral therapy. J Clin Virol. 2012 Jan;53(1):29-32.

29. SystemsX.ch. HIV-X - Deciphering Host-Virus Interactions to Cure HIV. 2018 [cited 12/01/2018]; Available from: http://www.systemsx.ch/projects/medical-research-and-development-projects/hiv-x/

30. Carrero-Gras A, Antela A, Munoz-Rodriguez J, Diaz-Menendez M, Viciana P, Torrella-Domingo A, et al. Nuke-sparing regimens as a main simplification strategy and high level of toxicity resolution after antiretroviral switch: the SWITCHART Study. J Int AIDS Soc. 2014;17(4 Suppl 3):19819.

31. Oddershede L, Walker S, Paton N, Stohr W, Dunn D, Sculpher M. Cost-effectiveness analysis of protease inhibitor monotherapy vs. ongoing triple-therapy in the long-term management of HIV patients. J Int AIDS Soc. 2014;17(4 Suppl 3):19498.

32. Schackman BR, Gebo KA, Walensky RP, Losina E, Muccio T, Sax PE, et al. The lifetime cost of current human immunodeficiency virus care in the United States. Med Care. 2006 Nov;44(11):990-7.

33. Ford N, Stinson K, Gale H, Mills EJ, Stevens W, Gonzalez MP, et al. CD4 changes among virologically suppressed patients on antiretroviral therapy: a systematic review and meta-analysis. J Int AIDS Soc. 2015;18(1):20061.

34. Ford N, Meintjes G, Pozniak A, Bygrave H, Hill A, Peter T, et al. The future role of CD4 cell count for monitoring antiretroviral therapy. Lancet Infect Dis. 2015 Feb;15(2):241-7.

35. Mugyenyi P, Walker AS, Hakim J, Munderi P, Gibb DM, Kityo C, et al. Routine versus clinically driven laboratory monitoring of HIV antiretroviral therapy in Africa (DART): a randomised non-inferiority trial. Lancet. 2010 Jan 9;375(9709):123-31.

36. Kekitiinwa A, Cook A, Nathoo K, Mugyenyi P, Nahirya-Ntege P, Bakeera-Kitaka S, et al. Routine versus clinically driven laboratory monitoring and first-line antiretroviral therapy strategies in African children with HIV (ARROW): a 5-year open-label randomised factorial trial. Lancet. 2013 Apr 20;381(9875):1391-403.

37. Laurent C, Kouanfack C, Laborde-Balen G, Aghokeng AF, Mbougua JB, Boyer S, et al. Monitoring of HIV viral loads, CD4 cell counts, and clinical assessments versus clinical monitoring

alone for antiretroviral therapy in rural district hospitals in Cameroon (Stratall ANRS 12110/ESTHER): a randomised non-inferiority trial. Lancet Infect Dis. 2011 Nov;11(11):825-33.

38. Boyer S, March L, Kouanfack C, Laborde-Balen G, Marino P, Aghokeng AF, et al. Monitoring of HIV viral load, CD4 cell count, and clinical assessment versus clinical monitoring alone for antiretroviral therapy in low-resource settings (Stratall ANRS 12110/ESTHER): a cost-effectiveness analysis. Lancet Infect Dis. 2013 Jul;13(7):577-86.

39. Kredo T, Adeniyi FB, Bateganya M, Pienaar ED. Task shifting from doctors to non-doctors for initiation and maintenance of antiretroviral therapy. Cochrane Database Syst Rev. 2014;7:CD007331.

40. Sanne I, Orrell C, Fox MP, Conradie F, Ive P, Zeinecker J, et al. Nurse versus doctor management of HIV-infected patients receiving antiretroviral therapy (CIPRA-SA): a randomised non-inferiority trial. Lancet. 2010 Jul 3;376(9734):33-40.

41. Ahn JY, Boettiger D, Law M, Kumarasamy N, Yunihastuti E, Chaiwarith R, et al. Implementation and Operational Research: Effects of CD4 Monitoring Frequency on Clinical End Points in Clinically Stable HIV-Infected Patients With Viral Suppression. J Acquir Immune Defic Syndr. 2015 Jul 1;69(3):e85-92.

42. Elzi L, Conen A, Patzen A, Fehr J, Cavassini M, Calmy A, et al. Ability to Work and Employment Rates in Human Immunodeficiency Virus (HIV)-1-Infected Individuals Receiving Combination Antiretroviral Therapy: The Swiss HIV Cohort Study. Open Forum Infect Dis. 2016 Jan;3(1):ofw022.

43. Cottrell ML, Hadzic T, Kashuba AD. Clinical pharmacokinetic, pharmacodynamic and drug-interaction profile of the integrase inhibitor dolutegravir. Clin Pharmacokinet. 2013 Nov;52(11):981-94.

44. Haas DW, Smeaton LM, Shafer RW, Robbins GK, Morse GD, Labbe L, et al. Pharmacogenetics of long-term responses to antiretroviral regimens containing Efavirenz and/or Nelfinavir: an Adult Aids Clinical Trials Group Study. J Infect Dis. 2005 Dec 01;192(11):1931-42.

45. Siccardi M, D'Avolio A, Rodriguez-Novoa S, Cuenca L, Simiele M, Baietto L, et al. Intrapatient and interpatient pharmacokinetic variability of raltegravir in the clinical setting. Ther Drug Monit. 2012 Apr;34(2):232-5.

46. Hashiguchi Y, Hamada A, Shinohara T, Tsuchiya K, Jono H, Saito H. Role of P-glycoprotein in the efflux of raltegravir from human intestinal cells and CD4+ T-cells as an interaction target for anti-HIV agents. Biochem Biophys Res Commun. 2013 Sep 20;439(2):221-7.

47. Tsuchiya K, Hayashida T, Hamada A, Oka S, Gatanaga H. Brief Report: High Peak Level of Plasma Raltegravir Concentration in Patients With ABCB1 and ABCG2 Genetic Variants. J Acquir Immune Defic Syndr. 2016 May 01;72(1):11-4.

48. Hightower KE, Wang R, Deanda F, Johns BA, Weaver K, Shen Y, et al. Dolutegravir (S/GSK1349572) exhibits significantly slower dissociation than raltegravir and elvitegravir from wild-type and integrase inhibitor-resistant HIV-1 integrase-DNA complexes. Antimicrob Agents Chemother. 2011 Oct;55(10):4552-9.

49. Katlama C, Soulie C, Blanc C, Denis A, Caby F, Schneider L, et al. Dolutegravir Monotherapy in HIV-Infected Patients with Suppressed HIV Viremia 15th Conference of the European AIDS Clinical Society. Barcelona, Spain 2015.

50. Underwood MR, Deanda F, Dorey D, Hightower K, Wang R, Griffith S, et al. Resistance Post Week 48 in ART-Experienced, Integrase Inhibitor-Naive Subjects with Dolutegravir (DTG) vs. Raltegravir (RAL) in SAILING (ING111762). 13th European HIV & Hepatitis workshop. Barcelona, Spain; 2015.

51. Quercia A, Roberts J, Murungi A, Curtis L, Payvandi N, Koteff J, et al. Psychiatric Adverse Events From the DTG ART-Naive Phase III/IIIb Clinical Trials. HIV Drug Therapy 2016. Glasgow, United Kingdom; 2016.

52. Baldin G, Borghetti A, Capetti A, Sterrantino G, Rusconi S, Latini A, et al. A comparison between tenofovir/emtricitabine/elvitegravir/cobicistat and dolutegravir-based three-drug regimens as switch strategies for virologically controlled, HIV-infected patients HIV Drug Therapy 2016. Glasgow, United Kingdom; 2016.

53. Fernandez C, Michie K, Thomson-Glover R, Chaponda M, Ratcliffe L. Adverse events and discontinuation of dolutegravir-based therapy in naïve and experienced patients: tertiary HIV centre experience. HIV Drug Therapy 2016. Glasgow, United Kingdom; 2016.

54. Madeddu G, Ricci E, Gulminetti R, Paola B, Squillace N, De Socio G, et al. Dolutegravir tolerability in clinical practice: results from the SCOLTA cohort. HIV Drug Therapy 2016. Glasgow,

United Kingdom; 2016.

55. Postel N, Mueller M, Wyen C, Brust J, Stoehr A, Glaunsinger T, et al. The DOL-ART cohort: providing evidence from real-world data - use of dolutegravir-based regimens in routine clinical care in Germany. HIV Drug Therapy 2016. Glasgow, United Kingdom; 2016.

56. Vivancos-Gallego MJ, Moreno A, Perez-Elias MJ, Gomez Ayerbe C, Casado JL, Quereda C, et al. Discontinuation of dolutegravir (DTG)-based regimens in clinical practice. HIV Drug Therapy 2016. Glasgow, United Kingdom; 2016.

57. de Boer MG, van den Berk GE, van Holten N, Oryszcyn JE, Dorama W, Moha DA, et al. Intolerance of dolutegravir-containing combination antiretroviral therapy regimens in real-life clinical practice. AIDS. 2016 Nov 28;30(18):2831-4.

58. Hoffmann C, Welz T, Sabranski M, Kolb M, Wolf E, Stellbrink HJ, et al. Higher rates of neuropsychiatric adverse events leading to dolutegravir discontinuation in women and older patients. HIV Med. 2017 Jan;18(1):56-63.

59. Walmsley S, Baumgarten A, Berenguer J, Felizarta F, Florence E, Khuong-Josses MA, et al. Dolutegravir Plus Abacavir/Lamivudine for the Treatment of HIV-1 Infection in Antiretroviral Therapy-Naive Patients: Week 96 and Week 144 Results from the SINGLE Randomized Clinical Trial. J Acquir Immune Defic Syndr. 2015 Aug 7.

60. Mulligan N, Best BM, Capparelli EV, Stek A, Barr E, Smith E, et al. Dolutegravir Pharmacokinetics in HIV-infected Pregnant and Postpartum Women. 23rd Conference on Retroviruses and Opportunistic Infections. Boston, USA; 2016.

61. Bürgi S. ViiV Healthcare Dolutegravir post-marketing surveillance data. 2016.

62. Zash R, Jacobson D, Mayondi G, Diseko M, Makhema J, Mmalane M, et al. Dolutegravir/tenofovir/emtricitabine (DTG/TDF/FTC) started in pregnancy is as safe as efavirenz/tenofovir/emtricitabine (EFV/TDF/FTC) in nationwide birth outcomes surveillance in Botswana. 9th International AIDS Conference on HIV Science Paris, France 2017.

63. European AIDS Clinical Society. Guidelines Version 9.0 October 2017. 2017 [cited; Available from: Guidelines Version 8.0 October 2015

64. Grinsztejn B, Coelho LE, Luz PM, Veloso VG. Towards an ideal antiretroviral regimen for the global HIV epidemic. J Virus Erad. 2017 Jul 1;3(3):111-6.

65. World Health Organization. Statement on DTG - Geneva 18 May 2018. 2018 [cited 01/07/2018]; Available from: http://www.who.int/medicines/publications/drugalerts/Statement_on_DTG_18May_2018final.pdf?ua=1

66. U.S. Food and Drug Administration. FDA to Evaluate Potential Risk of Neural Tube Birth Defects With HIV Medicine Dolutegravir (Juluca, Tivicay, Triumeq). 2018 [cited 01/07/2018]; Available from: http://www.thebodypro.com/content/81035/fda-to-evaluate-potential-risk-of-neural-tube-birt.html

67. European Medicines Agency. New study suggests risk of birth defects in babies born to women on HIV medicine dolutegravir 2018 [cited 01/07/2018]; Available from: http://www.ema.europa.eu/ema/index.jsp?curl=pages/news_and_events/news/2018/05/news_detail_002956.jsp&mid=WC0b01ac058004d5c1

68. Zash R, Jacobson DL, Diseko M, Mayondi G, Mmalane M, Essex M, et al. Comparative safety of dolutegravir-based or efavirenz-based antiretroviral treatment started during pregnancy in Botswana: an observational study. Lancet Glob Health. 2018 Jul;6(7):e804-e10.

69. Saag MS. Emtricitabine, a new antiretroviral agent with activity against HIV and hepatitis B virus. Clin Infect Dis. 2006 Jan 01;42(1):126-31.

70. Rousseau FS, Wakeford C, Mommeja-Marin H, Sanne I, Moxham C, Harris J, et al. Prospective randomized trial of emtricitabine versus lamivudine short-term monotherapy in human immunodeficiency virus-infected patients. J Infect Dis. 2003 Dec 01;188(11):1652-8.

71. World Health Organization HAP. Technical update on treatment optimization. Pharmacological equivalence and clinical interchangeability of lamivudine and emtricitabine: a review of the current literature 2012 June 2012.

72. Rojas J, Blanco JL, Montserrat L, Torres B, Parera M, Gonzales A, et al. Dolutegravir Monotherapy in HIV-infected Patients with Sustained Viral Suppression: A 24-week Pilot Study. 15th Conference of the European AIDS Clinical Society. Barcelona, Spain; 2015.

73. Oldenbuettel C, Wolf E, Ritter A, Noe S, Heldwein S, Pascucci R, et al. Dolutegravir

monotherapy as treatment de-escalation in HIV-infected adults with virological control: DoluMono cohort results. Antivir Ther. 2016 Sep 02.

74. Wijting I, Rokx C, Boucher CA, Van Kampen J, De Vries-Sluijs D, Schurink K, et al. Switching from cART to dolutegravir (DTG) maintenance monotherapy in virologically suppressed HIV-1 infected adults: a randomized multicenter, non-inferiority clinical trial (DOMONO). HIV Drug Therapy 2017. Glasgow, United Kingdom; 2016.

75. Wijting I, Rokx C, Boucher CA, de Vries-Sluijs TEMS, Schurink CAM, Andrinopoulou ER, et al. Dolutegravir as Maintenance Monotherapy for HIV-1: a Randomized Clinical Trial. Conference on Retroviruses and Opportunistic Infections 2017. Seattle, USA; 2017.

76. Martinez E. Personal communication on DOLAM study. In: Calmy A, editor.; 2017.

77. Blanco JL, Oldenbuettel C, Thomas R, Mallolas J, Wolf E, Brenner BG, et al. Comprehensive Assessment of Resistance Mutations Selected by Dolutegravir (DTG) in subjects Failing DTG-Monotherapy after Switching from other Therapies (Redomo Study). Conference on Retroviruses and Opportunistic Infections 2017. Seattle, USA; 2017.

78. Cahn P, Rolon MJ, Figueroa MI, Gun A, Patterson P, Sued O. Dolutegravir-Lamivudine as initial therapy in HIV-infected, ARV naive patients: 48 week results of the PADDLE trial 21st International AIDS Conference Durban, South Africa 2016.

79. Diaz A, Casado JL, Dronda F, Gomez-Ayerbe C, Vivancos MJ, Banon S, et al. Dolutegravir plus Rilpivirine in Suppressed Heavily Pre-Treated HIV-Infected Patients 21st International AIDS Conference. Durban, South Africa; 2016.

80. Oliveira M, Ibanescu RI, Pham HT, Brenner B, Mesplede T, Wainberg MA. The M184I/V and K65R nucleoside resistance mutations in HIV-1 prevent the emergence of resistance mutations against dolutegravir. AIDS. 2016 Jun 29.

81. Joly V, Burdet C, Landman R, Raffi F, Katlama C, Cabié A, et al. Promising results of Lamivudine + Dolutegravir Maintenance Therapy in ANRS 167 Lamidol Trial Conference on Retroviruses and Opportunistic Infections Seattle, USA; 2017.

82. Gunthard HF, Aberg JA, Eron JJ, Hoy JF, Telenti A, Benson CA, et al. Antiretroviral treatment of adult HIV infection: 2014 recommendations of the International Antiviral Society-USA Panel. JAMA. 2014 Jul 23-30;312(4):410-25.

83. Ministère des affaires sociales et de la Santé, editor. Prise en charge médicale des personnes vivant avec le VIH. Recommandations du groupe d'experts. Rapport 2013. Paris, France 2013.

84. European AIDS Clinical Society. Guidelines Version 8.0 October 2015. 2015.

85. Boillat-Blanco N, Darling KE, Schoni-Affolter F, Vuichard D, Rougemont M, Fulchini R, et al. Virological outcome and management of persistent low-level viraemia in HIV-1-infected patients: 11 years of the Swiss HIV Cohort Study. Antivir Ther. 2015;20(2):165-75.

86. Glass TR, Sterne JAC, Schneider MP, De Geest S, Nicca D, Furrer H, et al. Self-reported nonadherence to antiretroviral therapy as a predictor of viral failure and mortality: Swiss HIV Cohort Study. AIDS. 2015.

87. Duracinsky M, Herrmann S, Berzins B, Armstrong AR, Kohli R, Le Coeur S, et al. The development of PROQOL-HIV: an international instrument to assess the health-related quality of life of persons living with HIV/AIDS. J Acquir Immune Defic Syndr. 2012 Apr 15;59(5):498-505.

88. Duracinsky M, Lalanne C, Le Coeur S, Herrmann S, Berzins B, Armstrong AR, et al. Psychometric validation of the PROQOL-HIV questionnaire, a new health-related quality of life instrument-specific to HIV disease. J Acquir Immune Defic Syndr. 2012 Apr 15;59(5):506-15.

89. Pinto-Meza A, Serrano-Blanco A, Penarrubia MT, Blanco E, Haro JM. Assessing depression in primary care with the PHQ-9: can it be carried out over the telephone? J Gen Intern Med. 2005 Aug;20(8):738-42.

90. Kroenke K, Spitzer RL, Williams JB. The PHQ-9: validity of a brief depression severity measure. J Gen Intern Med. 2001 Sep;16(9):606-13.

91. Potterat MM, Monnin Y, Pechère A, Guesous I. [Women, forgotten by clinical research]. Revue Médicale Suisse. 2015(487):1733-6.

92. ANRS. HIV-1 genotypic drug resistance interpretation's algorithms. 2015 [cited 07/10/2015]; Available from: http://www.hivfrenchresistance.org/

93. Kohler P, Schmidt AJ, Cavassini M, Furrer H, Calmy A, Battegay M, et al. The HIV care cascade in Switzerland: reaching the UNAIDS/WHO targets for patients diagnosed with HIV. AIDS.

2015 Sep 13.

94. Zanchi A, Lehmann R, Philippe J. Antidiabetic drugs and kidney disease--recommendations of the Swiss Society for Endocrinology and Diabetology. Swiss Med Wkly. 2012;142:w13629.

95. U.S. Department of Health and Human Services, National Institute of Health, National Institute of Allergy and Infectious Diseases, AIDS. Do. Division of AIDS (DAIDS) Table for Grading the Severity of Adult and Pediatric Adverse Events, version 2.0. [November 2014]. 2014 [cited; Available from: http://rsc.tech-res.com/docs/default-source/safety/daids_ae_grading_table_v2_nov2014.pdf

96. Chow SC, Shao J, Wang H. Sample Size Calculations in Clinical Research, second edition. New-York; 2008.

97. Stellbrink HJ, Antinori A, Pozniak A, Flamm J, Bredeek F, Patel K, et al. Switch to Stribild versus continuation of NVP or RPV with FTC and TDF in virologically suppressed HIV adults: a STRATEGY-NNRTI subgroup analysis. J Int AIDS Soc. 2014;17(4 Suppl 3):19793.

98. Lennox JL, Dejesus E, Berger DS, Lazzarin A, Pollard RB, Ramalho Madruga JV, et al. Raltegravir versus Efavirenz regimens in treatment-naive HIV-1-infected patients: 96-week efficacy, durability, subgroup, safety, and metabolic analyses. J Acquir Immune Defic Syndr. 2010 Sep;55(1):39-48.

99. Eron JJ, Young B, Cooper DA, Youle M, Dejesus E, Andrade-Villanueva J, et al. Switch to a raltegravir-based regimen versus continuation of a lopinavir-ritonavir-based regimen in stable HIV-infected patients with suppressed viraemia (SWITCHMRK 1 and 2): two multicentre, double-blind, randomised controlled trials. Lancet. 2010 Jan 30;375(9712):396-407.

100. Bonnet M, Bhatt N, Baudin E, Silva C, Michon C, Taburet AM, et al. Nevirapine versus efavirenz for patients co-infected with HIV and tuberculosis: a randomised non-inferiority trial. Lancet Infect Dis. 2013 Apr;13(4):303-12.

101. Arribas J, Rizzardini G, Arasteh K, Zurawski C, Dietz C, Pontani D, et al. Simplification to Stribild vs continuation of RTV-boosted DRV with FTC and TDF in virologically suppressed HIV adults: a STRATEGY-PI subgroup analysis. J Int AIDS Soc. 2014;17(4 Suppl 3):19805.

102. Arribas JR, Girard PM, Landman R, Pich J, Mallolas J, Martinez-Rebollar M, et al. Dual treatment with lopinavir-ritonavir plus lamivudine versus triple treatment with lopinavir-ritonavir plus lamivudine or emtricitabine and a second nucleos(t)ide reverse transcriptase inhibitor for maintenance of HIV-1 viral suppression (OLE): a randomised, open-label, non-inferiority trial. Lancet Infect Dis. 2015 Jul;15(7):785-92.

103. Spagnuolo V, Galli L, Bigoloni A, Nozza S, Monforte A, Antinori A, et al. Atazanavir/ritonavir monotherapy as maintenance strategy in HIV-1 treated subjects with viral suppression: 96-week analysis results of the MODAT study. J Int AIDS Soc. 2014;17(4 Suppl 3):19806.

104. Pinnetti C, Lorenzini P, Cozzi-Lepri A, Sandrine O, Tommasi C, Zaccarelli M, et al. Randomized trial of DRV/r or LPV/r QD monotherapy vs maintaining a PI/r-based antiretroviral regimen in persons with suppressed HIV replication. J Int AIDS Soc. 2014;17(4 Suppl 3):19809.

105. Hintze J. PASS 13. 2014 [cited 12/10/2015]; Available from: www.ncss.com

106. Piaggio G, Elbourne DR, Pocock SJ, Evans SJ, Altman DG. Reporting of noninferiority and equivalence randomized trials: extension of the CONSORT 2010 statement. JAMA. 2012 Dec 26;308(24):2594-604.

107. Klingenberg B. A new and improved confidence interval for the Mantel-Haenszel risk difference. Stat Med. 2014 Jul 30;33(17):2968-83.

19. APPENDIX

1. Patient information and consent form
2. Protocol flyer
3. Case report form : worksheets and logs
4. Patient logbook: patient diary
5. List of interaction with dolutegravir
6. Adverse Event list
7. Information letter of 25.May.2018 to the Ethics Committee
8. Communication of 25.May.2018 to the DSMB

20. SUPPLEMENTARY FILES

SF1: Summary of presented or published studies on DTG monotherapy

Study	Details	Results	Comments
Retrospective analysis of treatment **experienced** patients who switched to DTG monoT (**ROJAS**)	N = 33; HIV duration, 19 years (IQR: 17–23); HIV suppression, 8 years (IQR: 4–13); 40% with history of AIDS	32/33 <37 copies/mL at W24; no change in viral dynamics at levels <37 copies/mL	1 case of viral rebound in complex patient with INSTI-experience and poor adherence; included INSTI mutation 118R at week 24
Single-arm observational pilot switch in treatment- **experienced** patients (**Katlama**)	N = 28; HIV duration, 20 years; HIV suppression, 6 years (IQR: 3–8)	25/28 <50 copies/mL at W24; 24/25 <20 copies/mL	3 cases of viral rebound in patients with prior INSTI experience, but with good adherence. All <50 cp/mL with triple therapy
Retrospective data from case notes in treatment- **experienced** patients switched to DTG monoT (**Gubavu**)	N = 52 (N = 21 monotherapy); follow-up, W27 (IQR: 24–40)	21/21 remained <50 cp/mL	No cases of viral rebound
Single-arm observational pilot study (**pilot for DOMDONO**)	N = 5 **experienced** pts	4/5 remained undetectable	Viral rebound included possible drug interaction with multivitamins.
RCT, 48W switch study in **experienced** pts (**DOMONO**)	N = 104 pts on cART for 40 months with CD4 nadir of 340; randomized to immediate switch to DTG monotherapy or deferred switch after 24W	**Confidential** (see e-mail by Bart Rijnders 21.1.20): when 77/96 on DTG monoT pts had reached W48 of monotherapy, **VF had developed in 8 (2 before W24, 6 after W24).** This led to the premature study discontinuation. Although DTG monoT was non-inferior to cART at W24, **VF continued to occur after W24 and led to DTG resistance in 3.** Final results at CROI 2017	
RCT in **experienced** pts with VL <50 cp/mL on current ART (**DOLAM**)	N = 450; randomized 1:1:1 to DTG, DTG + 3TC, or current treatment (control). Follow-up at BL, 4, 12, 24, 36, 48W.<br>Pts on stable triT with <50 cp/mL in ≥2 consecutive determinations for ≥12 months, no prior VF or resistance mutations to 3TC/FTC or INSTI, nadir CD4 >200cells/mm^3.	**Confidential** (see e-mail by Esteban Martinez on 9.2.2017): 2 VF in monoT arm at the end of phase A (90 pts, 24W follow-up). According to DSMB advise (called according to protocol safety rules "DSMB will review the data if the proportion of confirmed virological failure in any of the experimental arms reaches ≥5%"), the Steering Commitee has decided to interrupt the DTG arm and to continue the DOLAM study with two arms: CONTROL and DTG+3TC (no safety issues in dualtherapy arm). Results expected after October 2017	
Single-arm, open-label switch study in	N = 8 **experienced** pts, fully enrolled.	**Confidential**: 4 pts already at W24,	Final results expected in June 2017

patients with VL <50 copies/mL (**MONODO**)	Follow-up 24W	1pt at W20, 3 pts at ≤W12, no VF	
RCT in pts with suppressed VL on DTG/abacavir/3TC) (**MONCAY**)	N = 160, 48W follow-up Suppressed HIV pts with CD4 nadir ≥100 on DTG/ABC/3TC	Enrolling in France	Final results expected after April 2018
Retrospective observation 24 Week duration study (**Oldenbuettel**)	N=31 **experienced** pts suppressed ≥24W with no failure or resistance	1 discontinuation for adverse event (ongoing cough)<br>1 virological failure	1 VF at W24 with Q148H and G140S as INSTI mutations
RCT in **naive** patients treated for primary HIV infection with VL <50 cp/mL for at least W48 (**Early Simplified**)	N = 138; randomize 2:1 to DTG mono vs. current treatment (control), 192W follow-up	**Confidential** (see Grindelwald meeting 28.1.2017): 100 pts included, no VF up to now	Results expected after November 2019
Single-arm, open label, retrospective case notes (**Lanzafame**)	N = 9; **naive**; BL load (VL)<br>16,000–90,000 cp/mL; duration of infection 8 years	All VL <50 by W4 and<br><20 cp/mL by W24	Patient group: refused ART and only started because of the simplicity of DTG monoT

SF2: Summary of presented or published using dolutegravir dual therapy HIV-positive patients

Study	Details	Results	Comments
DTG + FTC: none			
DTG + 3TC			
56-week ANRS switch study in **experienced** virologically suppressed pts (**LAMIDOL**)	DTG + 3TC, N = 110 Includes semen substudy.	All patients at W40 of dual therapy. 101/104 (=97%) are in therapeutic success.<br>Last visit of the last patient is planned for March 2017.	- French National Institute for Health and Medical Research<br>- French ANRS<br>Collaborator: ViiV Healthcare
RCT, 48-week switchto open-label dual therapy vs. current ART (**ASPIRE**)	DTG + 3TC, N = 90 **Experienced**, suppressed pts	Currently enrolling in U.S.	Sponsored by ViiV
RCT in **experienced** pts with VL <50 cp/mL on current ART (**DOLAM**)	N = 450; randomized 1:1:1 to DTG, DTG + 3TC, or current treatment (control). Follow-up at BL, 4, 12, 24, 36, 48W.	**Confidential** (see e-mail by Esteban Martinez on 9.2.2017): 2 VF in monoT arm at the end of phase A (90 pts, 24W follow-up). According to DSMB advise (called according to protocol safety rules "DSMB will review the data if the proportion of confirmed virological failure in any of the experimental arms	

	Pts on stable triT with <50 cp/mL in ≥2 consecutive determinations for ≥12 months, no prior VF or resistance mutations to 3TC/FTC or INSTI, nadir CD4 >200cells/mm^3.	reaches ≥5%"), the Steering Commitee has decided to interrupt the DTG arm and to continue the DOLAM study with two arms: CONTROL and DTG+3TC (no safety issues in dualtherapy arm). Results expected after October 2017	
Observational, cohort DTG+3TC	N= 36 Heavly **experienced** pts	No viral failure	Italian cohort
48W follow-up single arm, pilot study (**NCT02211482**)	DTG + 3TC, **Naive** HIV pts, HIV-RNA >5000 and ≤10000, CD4 ≥200	Expected 02.2017	Sponsored by The Huesped Foundation, Viiv Healthcare as collaborators
24W follow-up single arm, pilot study (**NCT02582684**)	DTG + 3TC, **Naive** HIV pts,	Expected 01.2017	Sponsored by AIDS Clinical Trials Group, NIAID
RCT, 48 to 148 W follow-up in **naive** pts (Gemini 1)	DTG + 3TC vs. DTG + TDF/FTC, Naive pts	Expected 02.2024	Sponsored by ViiV and GSK
RCT, 48 to 148 W follow-up in **naive** pts (Gemini 2)	DTG + 3TC vs. DTG + TDF/FTC, Naive pts	Expected 02.2024	Sponsored by ViiV
Open-label, single-arm 48-week ACTG study in **naive** patients (**Roy Gulick: mail sent 7.2.16**)	DTG + 3TC, N = 120	Currently enrolling in U.S.	Results expected after November 2016
Single-arm, open-label, 96-week study in treatment-**naive** patients with VL <100,000 cp/mL (**PADDLE**)	DTG + 3CT N = 20; BL VL was >100,000 copies/mL in four patients	Interim results: 20/20 <400 cp/mL by W3 and 20/20 <50 cp/mL by W8, sustained to W24	Study ongoing for longer follow-up to 96 weeks
DTG + TDF: none			
DTG + NVP: none			
DTG + RIL:			
RCT, early switch phase D1 to W52 & late switch phase W52 to W148 (**SWORD-1: NCT 02429791; SWORD-2: NCT02422797**)	N= 1024 DTG + RPV vs. cART	DTG+RPV is non inferior to cART (primary endpoint: % of viral load <50 cp/ml at W48) Final results expected 08..2021	Sponsored by ViiV
RCT (DORISS)	N= 80 (planned) DTG + rilpivirine vs cART	Expected by 10.2017, but no patient currently enrolled	Sponsored by Nantes University Hospital

	48W follow up		
Observational Italian cohort (Capetti AF)	N= 132, 50 pts reached W48 of follow-up	At W48, 1 pts had low-level, 1 intermediate and 4 hig-level resistance to RPV. None failed.	No sponsor
DTG + DRV:			
Dualis	N = 320 (planned) DTG + DRV/r vs. DRV/r + TDF/FTC or ABC/3TC	Expected by March 2018	Technische Universität München
deNUC Study	DTG+DRV/c (immediate switch) vs. DTG+DRV/c (late switch at W24). Withdrawn prior to enrollment	Stanford University	
D²EFT	N = 610 (planned) DTG+DRV/r vs. cART	Not yet recruiting	Kirby Institute
Observational Italian cohort (Capetti AF)	N= 113, 48 pts reached W48 of follow-up	No virological failure, Italian cohort	No sponsor
Mixed			
48-W switch study (**DOLBI**)	DTG dual therapy (+RPV or +DRV/r or 3TC).<br>**Confidential**: > 150 pts currently included.	**Confidential**: excluding cases of non adherence, only 2 severely pre-treated pts switched to DTG+RPV had virological failure, in 1 case without emergence of resistances (the other not amplified). Final results expected by October 2017.	Asociacion para el Estudio de las Enfermedades Infecciosas
