# Supplementary material for: Efficacy and safety of dolutegravir plus emtricitabine versus standard ART for the maintenance of HIV-1 suppression: 48-week results of the factorial, randomized, non-inferiority SIMPL’HIV trial
Source: PLoS Med. 2020 Nov 10;17(11):e1003421. doi: 10.1371/journal.pmed.1003421 (PMC7654764; doi:10.1371/journal.pmed.1003421)
Supplement: Attachment — Submitted filename: Responses_to_reviewers.docx [file pmed.1003421.s008.docx]

**Manuscript entitled: “Efficacy and safety of dolutegravir plus emtricitabine versus standard ART for the maintenance of HIV-1 suppression: 48-week results of the factorial, randomized, non-inferiority SIMPL-HIV trial” by Sculier D, et al (revised version)**

I. **Requests from the editors:**
1. *Please quote the dates of start and end of study recruitment in the abstract, and participants' median age.*

As requested, this information is now added in the abstract as follows:

“Participants were enrolled between 12 May 2017 and 30 May 2018” (page 4, lines 79-80).

“(…. ) median age was 48 years” (page 4, line 94).

2. *Please add a new final sentence to the "Methods and findings" subsection of the abstract, quoting 2-3 of the study's main limitations.*

As requested, we have now added the following sentence at the end of the “Methods and findings” subsection of the abstract:

“The study’s main limitations included a rather small proportion of women included, the open label design, and a short 48 weeks duration.” (page 5, lines 109-111).

3. *At line 102, please start the sentence "In this study ..."*

As requested, “In this study” is now added in the conclusion subsection of the revised abstract (page 5, line 113).

4. After your abstract, we will need to ask you to add a new and accessible "author summary" section in non-identical prose. You may find it helpful to consult one or two recent research papers published in PLOS Medicine to get a sense of the preferred style.

As requested, we have added an author summary section (pages 6-7, from line 129 to line 167).

5. *At line 299 and any other instances, please use "White" rather than "Caucasian".*

As requested, we now used “White” instead of “Caucasian” (page 16, line 422; table 1, page 16, line 429).

6. *Please avoid claims of "the first", e.g. at line 408, and where needed add "to our knowledge" or similar.*

As requested, we have removed the claim of “the first” where mentioned (page 22, line 552; page 24, line 622).

7. *Throughout the text, please quote p values alongside 95% CI, where available. To clarify presentation of 95% CI, we suggest using "to" to indicate the range, rather than an em rule (e.g., "-7.8 to 5.6").*

As requested, we have clarified the presentation of the 95% CI where needed. We have also added the p-value where available (i.e. when pre-specified on our statistical analysis plan). This has now been reflected in the abstract and throughout the manuscript.

8. *Please convert reference call-outs to the following style throughout the text: "... maintenance therapy [1,2]." (noting the missing full point at line 113).*

As request, we have changed the style of the reference call-outs and added the missing full point (page 8, line 182).

9. *The details for some references seem incomplete, e.g., reference 16.*

We have now corrected the incomplete references on page 26-29.

10. *Please remove all instances of "[Internet]" from the reference list, and convert French text to English.*

We have now removed all instances of “[internet]” where present on pages 26-29 and converted the French text to English on pages 26-29.

11. *Please include a completed CONSORT checklist as a supplementary file, referred to in the Methods section (e.g., "See S1_CONSORT_Checklist"). In the checklist, please refer to individual items by section (e.g., "Methods") and paragraph number rather than by line or page numbers, as the latter generally change in the event of publication.*

As requested, we have included the CONSORT checklist as a supplementary file and now referred to in the Methods section (page 15, lines 399-400).

II. **Comments from the reviewers**

We would like to thank the reviewers for their detailed comments and constructive criticism, which has certainly helped to strengthen our manuscript.

Please see below our point-by-point response to each comment.

***** Reviewer #1: Statistical review**

1. *Abstract "patients were randomized 1:1:1:1…" I would recommend rewriting this sentence to emphasise that patients were randomised 1:1 between continuing cART and switching, and 1:1 between PCM and SM.*

We have now rephrased the sentence as follows:

“Patients virologically suppressed for at least 24 weeks on standard cART were randomized 1:1 to switching to DTG+ FTC or to continuing cART, and 1:1 to patient-centered simplified monitoring (PCM) or standard monitoring (SM)” (page 4, lines 83-85).

We have also included this change in the “Randomization” subsection of the Methods in the main text (page 10, lines 269-270).

2. *Abstract line 92/93 - I would recommend putting the non-inferiority margin earlier in the abstract and giving a p-value for non-inferiority in addition to the CI for risk difference.*

As pre-specified in our statistical analysis plan, we did not calculate a p-value for non-inferiority, but used the 95% CI to assess non-inferiority (ref: *Why the P-value is bad and confidence intervals a better alternative*, Ranstam J, Osteoarthritis and Cartilage 20 (2012) 805-808).

We have now included the other suggestion in the following sentence in the abstract:

“Non-inferiority of DTG+FTC vs. cART on viral suppression was assessed using a stratified Mantel-Haenszel risk difference and a 95% confidence interval, with a margin of -12%.” (page 4, line 88-90).

3. *Methods - there is no mention of blinding. the study was not double-blinded, but was there any blinding (e.g. rater-blinding?).*

We mentioned in the abstract that the study was open-label, i.e. there was no blinding at all, and emphasized the open-label characteristic of the study in the Methods section in the main text (page 9, line 231).

*4.        Methods, outcomes - I would recommend all secondary outcomes listed on the clinicaltrials.gov registration are mentioned in the main body of the paper (even if they are not reported in the paper).*

As suggested, we have now listed all the primary, secondary and additional endpoints listed on the clincatrials.gov registration in the Methods section (page 13, lines 335-342; 346-349; 353-357).

*5.        Methods, sample size calculation - "We primarily analyzed the trial as a stratified two-arm trial" - I would mention the stratification factor in this sentence (i.e. the monitoring type).*

We have modified the sentence as follows:

“We primarily analyzed the trial as a stratified two-arm trial comparing DTG+FTC with cART, monitoring type being the stratification factor” (page 14, line 375-376).

*6.        Methods, sample size calculation - was the sample size chosen to find a difference in monitoring types, if it existed? I see in the protocol this is based on cost differences, which are not reported in this paper - I would suggest it might be informative to add this to the sample size calculation and say that these outcomes are not reported in this paper.*

The sample size was determined for the primary comparison of DTG+FTC vs. cART in terms of viral suppression. For the secondary comparison of costs between monitoring types, we only performed power calculations.

As suggested, we have now stated that costs outcomes are not reported at the end of the Introduction section (page 9, lines 224-225), as well as in the outcomes subsection of the Methods section (pages 13, lines 357-359).

*7.        Results line 287 - I didn't follow what a randomisation occurring by mistake meant - was the patient ineligible?*

Indeed the patient was ineligible because of hepatitis B. This information was properly documented in the paper CRF, but the study nurse did not report this exclusion criteria in the electronic CRF and, therefore, the patient appeared to be eligible when (s)he was not. We have now added a brief explanation in parentheses in the text, i.e. “(ineligible patient from the start and mistakenly included)” (page 15, line 406-407).

*8.        Results - I would recommend all secondary endpoints are reported in a table in the main body of the paper, as opposed to in supplementary material.*

As suggested, we have added a table in the main body of the paper with secondary endpoints related to the primary comparison between DTG+FTC and cART arms. We have added the following sentence:

“Changes between week 48 and baseline in safety endpoints are presented in Table 4.” (page 20, lines 522-523; Table 4, page 20, line 524).

The co-primary and the secondary outcomes related to the comparison between the PCM and SM arms are not reported in this paper, but will be included in another manuscript in preparation. We would be pleased to submit it to PLOS Medicine once completed.

Of note, the outcome related to the changes in HIV-DNA will not be reported as the results were not ready when submitting our first manuscript in February 2020. Unfortunately, the analysis has been further delayed due to the COVID-19 situation. When available, these results may be the subject of a short communication at a future date.

*9.        Line 394 - I recommend adding 'significant' between 'no' and 'difference'*.

As suggested, this has now been added (page 21, line 537).

***** Reviewer #2:**

*1. More needs to be explicitly said regarding the relatively low number of female participants recruited to the study, in comparison to the gender proportions of the Swiss HIV cohort as a whole.*

We were indeed surprised not to achieve a similar proportion of female participants in our study as in the Swiss HIV Cohort Study as a whole.

At the present time, we do not have any clear explanation, but we may assume some hypotheses, including that there are some gender biais in research, both from the researcher side, as well as from the participant side. Therefore, to further explore this issue, we have designed a qualitative study to better understand barriers to female participation in clinical trials in Switzerland and we have now elaborated on this point in the Discussion section (page 24, lines 625-629).

*2. In general, the authors can consider making more explicit the rationale for the entire study - it is not immediately clear why the combination of DTG/FTC is required given that the evidence for DTG/3TC is fairly well-established, and FTC and 3TC are essentially clinically equivalent. A statement on this would clarify their position, as well as situate the study in the context of antiretroviral choices available to patients and prescribers.*

At the time of the writing of our protocol in 2015-2016, neither the study protocol (first posted on Clinicaltrial.gov on February 27, 2018, *NCT03446573)* nor the study results of the TANGO study (published on January 2020, *Clinical Infectious Diseases*, ciz1243, <https://doi.org/10.1093/cid/ciz1243>) nor its study protocol on *Clinicaltrials.gov* (first posted on February 27, 2018, *NCT03446573*) were available.

The rationale driving our study was as follows:

- An academic evaluation of a DTG-based dual combination
- If clinically equivalent (*Ford et al, Plos One 2013*) to 3TC, FTC at the dose of 200 mg once daily seems to have a greater intracellular activity and shows an in vitro greater potency as demonstrated in a randomized controlled trial (*Rousseau et al, JID 2003*). These advantages potentially led to the selection of FTC for subsequent triple ART fixed-dose combinations released later on the market (TDF/FTC/EFV, TD(A)F/FTC/RIL, TDF/FTC/DOR, TAF/FTC/BIC).
- To test the association of DTG+FTC among a study population with fewer eligibility constraints, in particular no nadir CD4 count or zenith HIV viral load restrictions, thus reflecting routine medical activity when caring for patients with HIV who are interested to reduce the number of active molecules to absorb and eager to reduce the cost burden to the health system.

Our trial was an academic-driven study. We therefore chose the association that we found the most appropriate at the time of elaborating the protocol and not based on commercial potential benefits.

We have therefore modified our paragraphs in the Introduction and Discussion sections to better reflect this:

- Page 8, lines 199-210 and page 9, lines 183-190:

“We aimed to investigate the efficacy and safety of DTG plus emtricitabine (DTG+FTC) dual therapy as this has not yet been explored to our knowledge”. FTC is similar to 3TC with respect to the convenience, safety and resistance profile, but it has a longer intracellular half-life activity and shows greater in vitro potency results that have potentially led to the selection of FTC for subsequently developed triple antiretroviral fixed-dose combinations” (11). Furthermore, “We aimed to examine efficacy and safety of dual therapy in a nationally-representative sample of PWHIV that includes a substantial percentage of women. We did not use any CD4 nadir or HIV-1 RNA zenith restrictions, thus mirroring routine care while reducing the number of drugs and costs to the health system”.

Page 25, line 491-492 and line 496:

In conclusion, this study provides evidence that the association of DTG+FTC expands the offer of two-drug simplification in maintenance treatment, especially as both drugs are available as generic formulation. The combination demonstrated non-inferiority in efficacy and safety, as well as a higher improvement in QoL over time compared to standard regimens. An ongoing assessment of data from the SIMPL’HIV study regarding health care-related costs and patient choices and satisfaction will provide additional insights into potential advantages of dual therapy and PCM.

*3. It is somewhat surprising that there was no significant difference in the QOL indices between participants undergoing SM and PCM. What are some of the postulated reasons for this?*

We were also concerned by these results. Enrolled participants had been living with HIV for a decade and were on treatment for the past 7 to 8 years. The most common patient-centered monitoring option chosen by participants was the telephone call instead of a physical health visit to the hospital.

In Switzerland, a small country, there is very little difference between a telephone call and a physical visit as patients do not need to travel a long distance to come to the hospital. We also believe that participants are already well satisfied with a high quality of life and have a high level of trust in the Swiss health system, which is easily accessed, and the regular monitoring in place with their HIV doctors. Thus, our postulate that treatment optimization should also include simplification of HIV monitoring appears not to be valid for Switzerland. We have now added a sentence to discuss this point on page 22, line 562-564:

“However, we did not observe changes between the monitoring groups, probably reflecting the high level of trust in the health care system and facilitated access in Switzerland.”

***** Reviewer #3:**

*1. Global Recommendations:*

*1.1. Instead of "HIV-1 infected adults" use "people living with HIV (PLWH)"*

As suggested, we have removed the term “HIV-infected”, which is not appropriate and have replaced it with “people with HIV (PWHIV)”. Indeed, the “L” is redundant and many guidelines, such the DHHS and IAS-USA, have moved away from PLHIV and use either PWH or PWHIV. This has now been reflected in the abstract and throughout the manuscript.

*2. Abstract:*

*2.1. On page 4, they define virologic suppression as having < 100 copies/mL. Later on page 7, under the methods section, they state that they "define virological suppression as HIV-11 RNA of < 50 copies/mL"*

On page 4, we defined our primary endpoint in the Methods subsection of the abstract, i.e. the proportion of patients achieving viral suppression, defined as a viral load <100 copies/mL through 48 weeks.

On page 9-10, in the Methods section of the main text, we defined our main eligibility criteria: to be eligible, participants must have a viral load suppression <50 copies for 6 months, although one blip was allowed. For a better understanding by the reader, we have rephrased the sentence as:

“Patients were eligible if they were on any of the EACS-recommended cART (13) and virologically suppressed for at least 24 weeks prior to enrolment with virological suppression defined as HIV-1 RNA of <50 copies/ml, although we allowed a single blip of <200 copies/ml in the past six months.” (page 9 lines 232-254).

*3. Introduction:*

*3.1. Need more convincing of why this research was conducted? The pill burden would be increasing for the patient which could lead to decreased adherence and worse clinical outcomes. Why would you ever make this switch in clinical practice when there is dolutegravir/lamivudine?*

This point was also addressed by reviewer #2.

At the time of the writing of our protocol in 2015-2016, neither the study protocol (first posted on Clinicaltrial.gov on February 27, 2018, *NCT03446573)* nor the study results of the TANGO study (published on January 2020, *Clinical Infectious Diseases*, ciz1243, <https://doi.org/10.1093/cid/ciz1243>) nor its study protocol on *Clinicaltrials.gov* (first posted on February 27, 2018, *NCT03446573*) were available.

The rationale driving our study was as follows:

- An academic evaluation of a DTG-based dual combination
- If clinically equivalent (*Ford et al, Plos One 2013*) to 3TC, FTC at the dose of 200 mg once daily has a greater intracellular activity and shows an in vitro greater potency as demonstrated in a randomized controlled trial (*Rousseau et al, JID 2003*). These advantages potentially led to the selection of FTC for subsequent triple ART fixed-dose combinations released later on the market (TDF/FTC/EFV, TD(A)F/FTC/RIL, TDF/FTC/DOR, TAF/FTC/BIC).
- To test the association of DTG+FTC among a study population with fewer eligibility constraints, in particular no nadir CD4 count or zenith HIV viral load restrictions, thus reflecting routine medical activity when caring for patients with HIV who are interested to reduce the number of active molecules to absorb and eager to reduce the cost burden to the health system.

Our trial was an academic-driven study. We therefore chose the association that we found the most appropriate at the time of elaborating the protocol and not based on commercial potential benefits.

We have therefore modified our paragraphs in the Introduction and Discussion sections to better reflect this:

- Page 8, lines 199-182 and page 9, lines 183-190:

“We aimed to investigate” the efficacy and safety of DTG plus emtricitabine (DTG+FTC) dual therapy which has not yet been examined to our knowledge. FTC is similar to 3TC with respect to the convenience, safety and resistance profile, but it has a longer intracellular half-life activity “and shows greater in vitro potency results that have potentially led to the selection of FTC for subsequently developed triple antiretroviral fixed-dose combinations” (11). Furthermore, “we aimed to” examine efficacy and safety of dual therapy in a nationally-representative sample of PWHIV that includes a substantial percentage of women. We did not use any CD4 nadir or HIV-1 RNA zenith restrictions, thus mirroring routine care while reducing the number of drugs and costs to the health system.”

Page 25, line 667-674:

In conclusion, this study provides evidence that the association of DTG+FTC expands the offer of two-drug simplification in maintenance treatment, especially as both drugs are available as generic formulation. The combination demonstrated non-inferiority in efficacy and safety, as well as a higher improvement in QoL over time compared to standard regimens. An ongoing assessment of data from the SIMPL’HIV study regarding health care-related costs and patient choices and satisfaction will provide additional insights into potential advantages of dual therapy and PCM.

*3.2. On page 7, in the introduction section, there is a statement that says "As a second primary objective, the study aimed to compare the costs of the patient-centered monitoring (PCM) approach with standard 3-monthly routine surveillance" - within the primary outcome section, there is no mention of the results of this objective. It is never addressed.*

Indeed, this objective is not addressed as we stated that “Data presented here are primarily restricted to the comparison between DTG+FTC and cART in terms of efficacy, safety and QoL”.

As this comment was also raised by reviewer #1, we have now added the following sentence: “cost outcomes are not reported in this manuscript” (page 9, lines 224-225).

*4. Methods:*

*4.1. On page 4, they define virologic suppression as having < 100 copies/mL. Later on page 7, under the methods section, they state that they "define virological suppression as HIV-11 RNA of < 50 copies/mL".*

On page 4, we defined our primary endpoint in the Methods subsection of the abstract, i.e. the proportion of patients achieving viral suppression, defined as a viral load <100 copies/mL through 48 weeks.

On page 9-10, in the Methods section of the main text, we defined our main eligibility criteria: to be eligible, participants must have a viral load suppression <50 copies for 6 months, although one single blip episode (below 200 copies/mL) was allowed. For a better understanding by the reader, we have rephrased the sentence as:

“Patients were eligible if they were on any of the EACS-recommended cART (13) and virologically suppressed for at least 24 weeks prior to enrolment with virological suppression defined as HIV-1 RNA of <50 copies/ml, although we allowed a single blip of <200 copies/ml in the past six months.” (page 9-10 lines 232-254).

*5. Results:*

*5.1. Table 1, the demographic between the study group and they cART group. Based on the patient information on the table, the DTG+FTC group appears to be more compromised than the cART group, The DTG+FTC patient's median CD4 baseline is low, while the medical viral load was higher. Additionally, the DTG+FTC group had been receiving ART longer than the cART group, on average.*

Indeed, the DTG+FTC group seems to be more fragile than the cART group, but differences were not statistically significant as the interquartile ranges overlap quite widely. If the difference was statistically significant, we would have emphasized this point as it is an additional argument for the efficacy and we use of DTG maintenance dual therapy. However, it reflects the “real-life” HIV population that we see in clinics and less in industry-sponsored trials.

*5.2. An aspect I found interesting was that if you look at table 3 on page 17, there appear to be substantially more serious adverse effects from the cART group compared to the DTG+FTC group. This was not highlighted in the article.*

We have mentioned this difference regarding more frequent adverse events in the cART group compared to the DTG+FTC group in the Results section, page 19 of the first version submitted, lines 498-500. Similarly, we mentioned it in the Discussion section, page 24, lines 630-634, of the first version submitted, explaining that this difference may be due to the open-label design and bias induced among physicians (and probably participants) in reporting adverse events, especially as all participants were stable on standard cART before the start of the study and indeed, such a difference was not expected.

*5.3. On page 17, they point out that the CrCl for the DTG+FTC group is significantly lower than the cART patients. The authors do not offer any explanation of why they believe this to be happening.*

We discussed this finding in the Discussion section, page 23, lines 594-597, of the first submitted version. DTG is known to decrease the tubular secretion of creatinine and thus induce changes in the serum creatinine (which increases) and creatinine clearance (which decreases).

It is true that we did not mention it clearly and thus we have now added this sentence in the Discussion section (page 23, lines 594-597):

# “We observed a significantly lower estimated creatinine clearance in the DTG+FTC compared to the cART arm. DTG is known to increase serum creatinine and reduce creatinine clearance by inhibiting the tubular secretion of creatinine”.

# However, we did not measure the cystatin C level and therefore cannot judge if this finding is clinically relevant.
