# Supplementary material for: Efficacy and safety of dolutegravir plus emtricitabine versus standard ART for the maintenance of HIV-1 suppression: 48-week results of the factorial, randomized, non-inferiority SIMPL’HIV trial
Source: PLoS Med. 2020 Nov 10;17(11):e1003421. doi: 10.1371/journal.pmed.1003421 (PMC7654764; doi:10.1371/journal.pmed.1003421)
Supplement: Attachment — Submitted filename: Response to the editors.doc [file pmed.1003421.s009.doc]

**Manuscript entitled: “Efficacy and safety of dolutegravir plus emtricitabine versus standard ART for the maintenance of HIV-1 suppression: 48-week results of the factorial, randomized, non-inferiority SIMPL-HIV trial” by Sculier D, et al (final version)**

**Response to Requests from Editors (email received on 26 September 2020):**

*1. We note that your data statement describes all relevant data as being available in the article and supplementary files. To comply with PLOS' data policy please include patient-level data in supplementary files, or arrange to make these available at a public repository, say (*[*https://journals.plos.org/plosmedicine/s/data-availability*](https://journals.plos.org/plosmedicine/s/data-availability)*).*

We have updated the Data Availability Statement in the submission form with the following sentence: Data underlying the reported findings are provided upon request. Instructions with contact information as well as further relevant documents are available at the Bern Open Repository (BORIS): <https://boris.unibe.ch/id/eprint/146808>

*2. In your abstract, rather than "Noninferiority margin ... was -12%", please adapt the explanation to match that around line 330, i.e., "noninferiority was declared if the lower bound of the 95% confidence interval was greater than -12%" or similar.*

As requested, we adapted the sentence in the abstract section to match with the explanation in the Methods section, on page 4, lines 72-73.

*3. Please mention both ITT and per protocol analyses in the abstract, e.g., by specifying that the primary analysis used ITT (assuming this is correct) and noting that the results of a per protocol analysis were similar.*

As requested, we mentioned both ITT and PP analysis in the abstract, by adding the sentence “Per-protocol analysis showed similar results with viral suppression of 96.5% in both arms (risk difference: 0.0%; 95% CI -5.6 to 5.5%)” on page 4, lines 79-81.

*4. Please add a sentence, say, to the abstract to provide additional information on adverse events. We would suggest quoting the higher incidence of serious adverse events in the cART arm, and noting that events were not judged to be related to study medication.*

As requested, we provided additional information on adverse events in the abstract and added the following sentence: “The proportion of serious adverse events was higher in the cART arm (16%) compared to the DTG+FTC arm (6.5%) (*p*=0.041) but none was considered to be related to the study medication” on oage 4, lines 87-89.

*5. Please reformat the author summary with bullet points, aiming for around 3 points in each of the three subsections. Please trim the individual points, which should consist of no more than 1-2 short sentences each*.

As requested we modified the author summary section with changes seen on pages 6 and 7, from line 105 to line 143.

*6. Please correct "jeopardizing" in the author summary.*

This was corrected as requested on page 7, line 130.

*7. In the methods section, we notice that the noninferiority criterion is discussed around lines 313 and 330. We suggest avoiding repetition.*

According to our statisticians, we have to mention the non-inferiority margin both in the sample size calculation and in the analysis. We however tried to shorten the sentence on page 15, lines 319-320.

*8. Please change "gender" to "sex" where appropriate, e.g., at line 536.*

This was done as requested, on page 24, line 528.

*9. Throughout the text, please format reference call-outs as follows: "... clinical trials [9,10].".*

As requested, this was done throughout the manuscript.

*10. Please review the reference list carefully. In some cases the journal names need to be adapted, e.g., "Lancet" will suffice for reference 35; and all italics should be removed, e.g., from reference 36.
11. Noting reference 8, please ensure that all references contain full access information.
12. Please remove all elements of French spelling, e.g. "Jan" rather than "janv" for reference 31.
13. Please remove all examples of "[Internet]", e.g., from reference 30. Please also spell out the institutional author name for this reference.*

We reviewed the full list of references and used another program for citing references. Therefore, they are all modified. Pages 27 to 32, lines 603 to 741.

*14. Please remove the page numbers from the attached CONSORT checklist - individual items should be referred to by article section, as at present, and paragraph number.*

As requested, this was done in the Supplementary file: S1_Consort_Checklist.doc

**Response to Production Issues (email received on 29 September 2020):**

FIGURES and GRAPHICS:

*1. Please provide all main figures in .tif or .eps format.
2. Please ensure that all main figures are referenced in the manuscript as Fig 1, Fig 2, etc. (including capitalization), rather than Figure 1, Figure 2, etc. Please note, however, that the file names themselves must not include the space, i.e. Fig1.tif, Fig2.tif. Please ensure that all main figure files are clearly labeled as such in the File Inventory.*

All figures have been uploaded in .tif format, referenced in the manuscript as requested (page 10, line 215; page 15, line 339; page 16, line 348; page 17, line 370; page 18, line 396; page 19, line 414), and clearly labeled in the File Inventory.

METADATA:

*1. Please accurately complete the 'Competing Interests' section on our submission form, including any COIs declared by your co-authors. If you have no competing interests to declare, please add "The authors have declared that no competing interests exist". Otherwise please declare all competing interests beginning with the statement "I have read the journal's policy and the authors of this manuscript have the following competing interests:" As your authors have declared COI, please use the latter sentence.

2. Your co-author, Matthias Egger, serves on our Editorial Board. Please include the following sentence as part of your competing interest statement: "ME is a member of the Editorial Board of PLOS Medicine".*

We have completed the “Competing Interests” section has requested in the submission form with adding the sentences: "I have read the journal's policy and the authors of this manuscript have the following competing interests:" and "ME is a member of the Editorial Board of PLOS Medicine".

3. In accordance with PLOS' data policy, please ensure that your Data Availability Statement in the submission form clearly identifies how readers can access your data. Note that it will be typeset as it is written so please ensure it is in complete sentences and appears as you would like it to in the published version.

We have updated the Data Availability Statement in the submission form. Our datasets are available as an open access item with a Creative Commons Attribution license (CC-BY) at the Bern Open Repository (BORIS).

ARTICLE FILE:

*1. Please indicate the corresponding author on the title page with an asterisk (*) and include the corresponding author's email address. Only an asterisk and the email address itself are required. Please format as follows, omitting all other name, address and contact information:
** [*delphine.sculier@hin.ch*](mailto:delphine.sculier@hin.ch)

We have made the change as requested, page 1, line 7 and page 2, line 41

*2. Please ensure that all author names are spelled correctly in the manuscript file and the submission form, including the use of a middle name/initials, and any special characters (accents, umlauts, etc.). Please place first/given names before last/family names. We ask that you ensure they match verbatim. Please be aware that correct spelling and formatting of author names in the article file is vital for accurate indexing in PubMed.
The following author name does not match verbatim across the article file and the submission form: Laurent Decosterd (Laurent A. Decosterd)*

We have reviewed all author names and spelling in the manuscript file and submission form and only corrected Laurent A. Decosterd in the manuscript file, page 1, line 9.

*3. As your paper involves contributions from Swiss HIV Cohort Study, please format your paper as follows:
1) Please list Swiss HIV Cohort Study as an author in the byline.
2) If you wish to include the names of individual group members in the manuscript, you may list them in the Acknowledgments section.
Please be aware that group author members are no longer indexed in PubMed as collaborators.*

The Swiss HIV Cohort Study was already listed as an author. We added membership of the SHCS in the Acknowledgments section.

4. Please ensure that your Ethics Statement is available in the Methods section of your manuscript in its entirety, under the subheading "Ethics Statement".

We have added the Ethics Statement subheading in the Methods section, on page 15, line 332.

*5. We have noted that you have the following present in your manuscript: Abbreviations list/* *List of keywords/ Author Contributions
We have removed these section from your manuscript file as this information is automatically pulled from the submission form. Please make sure that the information in the submission form is correct.*

We have checked that the information related to List of keywords and Author Contributions in the submission form are correct. However, we did not find the Abbreviations list in the submission form so we could not checked it.  *6. You refer to "Appendix S1" and "Appendix S2" on page 15 of your manuscript. PLOS does not publish Appendices in the main manuscript. Please incorporate them into the main manuscript, or upload each of your Appendices as a Supporting Information file in the form "S1 Text", “S2 Text” etc, and refer to it throughout the manuscript as such.*

We have incorporated our Appendix S1 and S2 as Supporting Information and named them accordingly in the manuscript, on page 15, lines 330 and 335.

SUPPORTING INFORMATION:

*1. Please ensure that all supporting information files that are intended for publication have the file type 'Supporting Information' in the File Inventory.*

We have ensured that all supporting information files have the correct file type in the File Inventory.

*2. Your CONSORT checklist will need to be published with your article. Please therefore ensure that it has the file type 'Supporting Information' rather than file type ‘Other’ in the File Inventory and ensure it adheres to our naming conventions.*

We have included the CONSORT Checklist as a supporting information file.

*3. Please replace the page numbers in your CONSORT statement with paragraph numbers per section (e.g. "Methods, paragraph 1") since the page numbers of the final published paper may be different from the page numbers in the current manuscript.*

We have made the changes as requested in the CONSORT Checklist file.

*4. Please upload your Supporting Information items individually, rather than in a single file, as you refer to them individually in the text. If you would like to retain the single file please rename internal items (e.g. tables) as A, B, C etc. Please also provide a legend for each under the heading Supporting Information. Supporting Information files should be less than 10 MB each. Please edit any citation of internal items within your Supporting Information files to using letters rather than numbers, e.g. “Table A in S1 Text”. Please ensure these changes are made throughout the manuscript and within the Supporting Information files themselves.*

We have uploaded the Supporting Information files individually as requested in the File Inventory.

5. Please ensure that all Supporting Information files use the following format verbatim throughout the manuscript (including legends and all in-text citations): S1 Fig, S1 Table, S1 Text, S1 Data, etc. Each file type should start at 1 and increase numerically. Please note, however, that the file names themselves must use an underscore rather than a space, i.e. S1_Fig.tif, S1_Table.xlsx, S1_Text.doc, S1_Data.xlsx, S1_PRISMA_Checklist.docx, etc.

We have referred to the Supporting Information files throughout the manuscript and have named the files themselves as requested.

*6. Please ensure that each supporting information file has a legend at the end of the manuscript file, after the Reference list.*

We have added the Supporting Information Legend at the end of the manuscript after the References section, on page 33, from line 750 to 764.

*7. Please note that your submission cannot contain any references to generic "supporting information" files and instead must refer to specific files using our naming convention (S1 Fig, S1 Table, S1 Text, S1 Data, etc.)*

As requested, we have used your naming convention.
